# Synthesis and conformational analysis of linear homo- and heterooligomers from novel 2-*C*-branched sugar amino acids (SAAs)

**DOI:** 10.1038/s41598-018-24927-6

**Published:** 2018-04-26

**Authors:** Guang-Zong Tian, Jing Hu, Heng-Xi Zhang, Christoph Rademacher, Xiao-Peng Zou, Hong-Ning Zheng, Fei Xu, Xiao-Li Wang, Torsten Linker, Jian Yin

**Affiliations:** 1 Key Laboratory of Carbohydrate Chemistry and Biotechnology, Ministry of Education, School of Biotechnology, Jiangnan University, Lihu Avenue 1800, Wuxi, Jiangsu 214122 P.R. China; 2 Wuxi School of Medicine, Jiangnan University, Lihu Avenue 1800, Wuxi, Jiangsu 214122 P.R. China; 3 Department of Biomolecular Systems, Max Planck Institute of Colloids and Interfaces, Am Mühlenberg 1, Potsdam, 14476 Germany; 4 Department of Chemistry, University of Potsdam, Karl-Liebknecht-Str. 24-25, Potsdam, 14476 Germany

## Abstract

Sugar amino acids (SAAs), as biologically interesting structures bearing both amino and carboxylic acid functional groups represent an important class of multifunctional building blocks. In this study, we develop an easy access to novel SAAs in only three steps starting from nitro compounds in high yields in analytically pure form, easily available by ceric (IV) mediated radical additions. Such novel SAAs have been applied in the assembly of total nine carbopeptoids with the form of linear homo- and heterooligomers for the structural investigations employing circular dichroism (CD) spectroscopy, which suggest that the carbopeptoids emerge a well-extended, left (or right)-handed conformation similar to polyproline II (PPII) helices. NMR studies also clearly demonstrated the presence of ordered secondary structural elements. 2D-ROESY spectra were acquired to identify ^**i+1**^**NH ↔ **^**i**^**C**_**1**_**H**, ^**i**^**C**_**2**_**H** correlations which support the conformational analysis of tetramers by CD spectroscopy. These findings provide interesting information of SAAs and their oligomers as potential scaffolds for discovering new drugs and materials.

## Introduction

**Figure 1:**
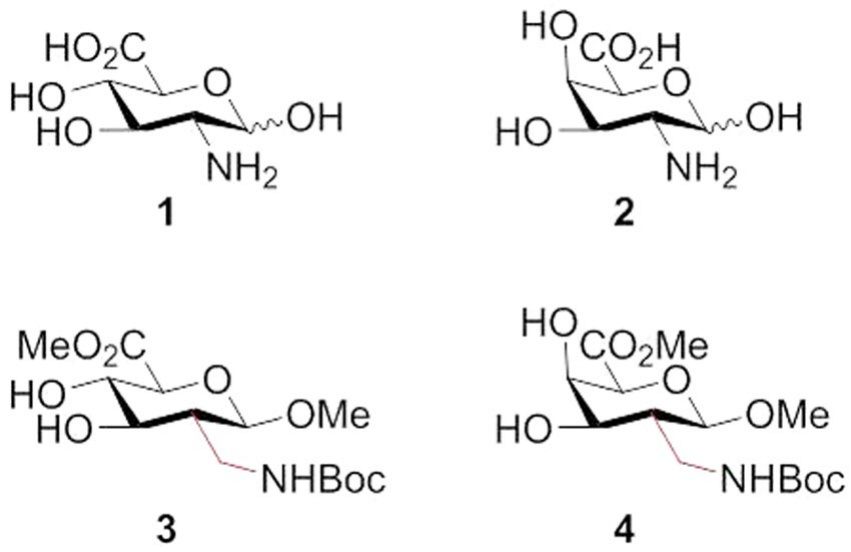
Glucosaminuronic acid **1** & galactosaminuronic acid **2** and **2**- *C*-branched glucosaminuronic acid **3** & galactosaminuronic acid **4**.

Sugar amino acids, as carbohydrate derivatives bearing both amino and carboxylic acid functional groups, represent an important class of multifunctional building blocks for discovering new drugs and materials^1–15^. A plenty diversity of SAAs have been designed and synthesized by many research groups in the past decades, because they are amenable to serve as glycomimetics or peptidomimetics with well-defined structures and distinct biological properties^16–36^. Glucosaminuronic acid **1** and galactosaminuronic acid **2** (Fig. 1), as constituents of cell walls of many bacteria, are important biomolecules, which have been discussed as potential vaccine antigens for the prevention of influenza virus and *Staphylococcus aureus*^37,38^. Recently, glycosaminuronic acid analogues have been investigated as promising candidates for therapeutic agents^39,40^. Attractive structures are 2-*C*-branched aminuronic acids **3** and **4** (Fig. 1), which represent as biologically potential glycomimetics.

## Results

### The synthesis of novel 2-*C*-branched sugar amino acids

During the course of our investigations on transition metal mediated radical reactions, we have established ceric (IV) mediated radical additions in carbohydrate chemistry for two decades^41,42^. Starting from easily available glycals and various CH-acidic precursors the reactions proceed in only one step with high selectivities in good yields. The products allow various transformations, which offer a general entry to diverse carbohydrate 2-*C*-analogues and demonstrate the power of radical reactions in natural product chemistry^43–53^.

**Figure 2:**
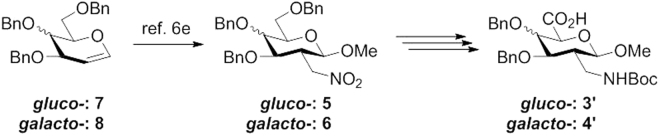
Proposed synthesis of SAAs **3′** and **4′**. Reagents and conditions: (**a**) H2, 10% Pd/C, MeOH; Boc2O, NaOH, MeOH/H2O (3:1), 64%; (**b**) TIPSCl, imidazole, DMF, 89%; (**c**) BnBr, 18-crown-6, KOH, THF, 71%; (**d**) TBAF, THF, 98%; (**e**) TEMPO, NaBr, TBABr, NaOCl; quan.; (**f**) ZnCl2, Ac2O/AcOH (2:1), 85%; (**g**) LiAlH4, THF; Boc2O, NaOH, MeOH/H2O (3:1), 64%; (**e**’) TEMPO, NaBr, TBABr, NaOCl; quan.; (**f**’) ZnCl2, Ac2O/AcOH (2:1), 77%; (**g**’) LiAlH4, THF; Boc2O, NaOH, MeOH/H2O (3:1), 46%.

**Figure 3:**
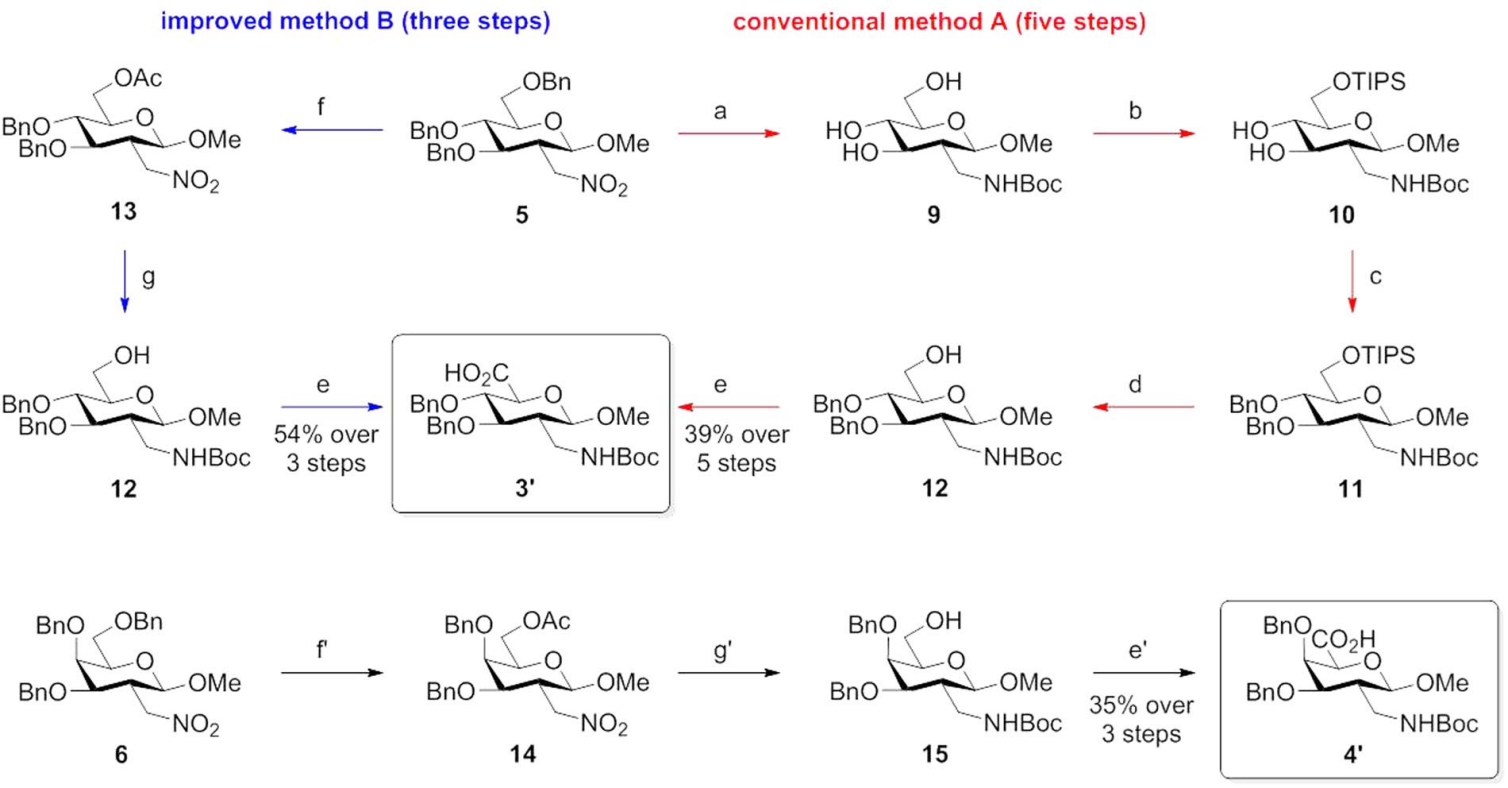
A convenient method to access novel 2-*C*-branched sugar amino acids *gluco*-**3′** and *galacto*-**4′**.

Herein we describe a convenient entry (Fig. 2) to access novel 2-*C*-branched sugar amino acids (**3′** and **4′**) starting from 2-deoxy-2-*C*-nitromethylpyranosides (**5** and **6**), which are easily available on a large scale via radical addition of nitromethane to tri-*O*-benzyl-D-glucal **7** and tri-*O*-benzyl-D-galactal **8**^48^. At first, radical addition product **5** was applied for the tentative synthesis of SAA **3′**. Initial transformation was conducted in the presence of hydrogen gas and catalytic Pd/C to remove three benzyl protecting groups, and to afford the primary amino group simultaneously, followed with the introduction of di-*tert*-butyl dicarbonate to lead to polar compound **9** in 64% yield. The primary hydroxyl group at 6-position of **9** was protected selectively by using triisopropylsilyl chloride in high yield. The subsequent benzylation and removal of TIPS protecting groups gave the intermediate **12** smoothly. Final oxidation was performed employing TEMPO/NaOCl, which completed the transformation from hydroxyl group to carboxylic acid quantitatively. As a targeted sugar amino acid, 2-*C*-branched glucosaminuronic acid **3′** was obtained in five steps in overall 39% yield by using the conventional method A. To meet the demand of facial synthesis of such interesting molecules, a superior synthetic route was needed to replace the routine entry. Reexamining method A, selectively exposing the hydroxyl group at 6-position was considered as the key issue to shorten the number of steps and possibly increase the overall yield. Since selective 6-*O*-debenzylation of methyl glycoside derivatives is a useful procedure for the synthesis of 1,6-linked oligosaccharides, various strategies have been developed^54–57^, for example, by using FeCl_3_/Ac_2_O, ZnI_2_/Ac_2_O, ZnCl_2_/Ac_2_O, TMSOTf/Ac_2_O or Iodotrimethylsilane. However, the effectively direct 6-*O*-debenzylation of addition product **5** was only realized in the presence of ZnCl_2_/Ac_2_O/HOAc in 85% yield developed by the Kong group^58^ rather than other catalysts, which unfortunately gave the desired product alongside many unexpected masses. Furthermore, the important intermediate **13** was transferred to compound **12** employing lithium aluminium hydride to reveal hydroxyl and amino groups simultaneously, followed with the introduction of di-*tert*-butyl dicarbonate to protect free amino group. Final oxidation by using TEMPO/NaOCl yielded targeted SAA **3′** quantitatively, which was obtained in only three steps in overall 54% yield starting from addition product **5**. With the improved method B (Fig. 3) in hand, 2-*C*-branched galactosaminuronic acid **4′** was synthesized in 3 steps in overall 35% yield starting from radical addition product **6** (Fig. 3). The final debenzylated compounds **3** and **4** were obtained after simple transformations of **3′** and **4′**. Thus, we have developed an easy entry to interesting glycosaminuronic acid analogues in high yields.

### The assembly of oligomers from sugar amino acids

**Figure 4:**
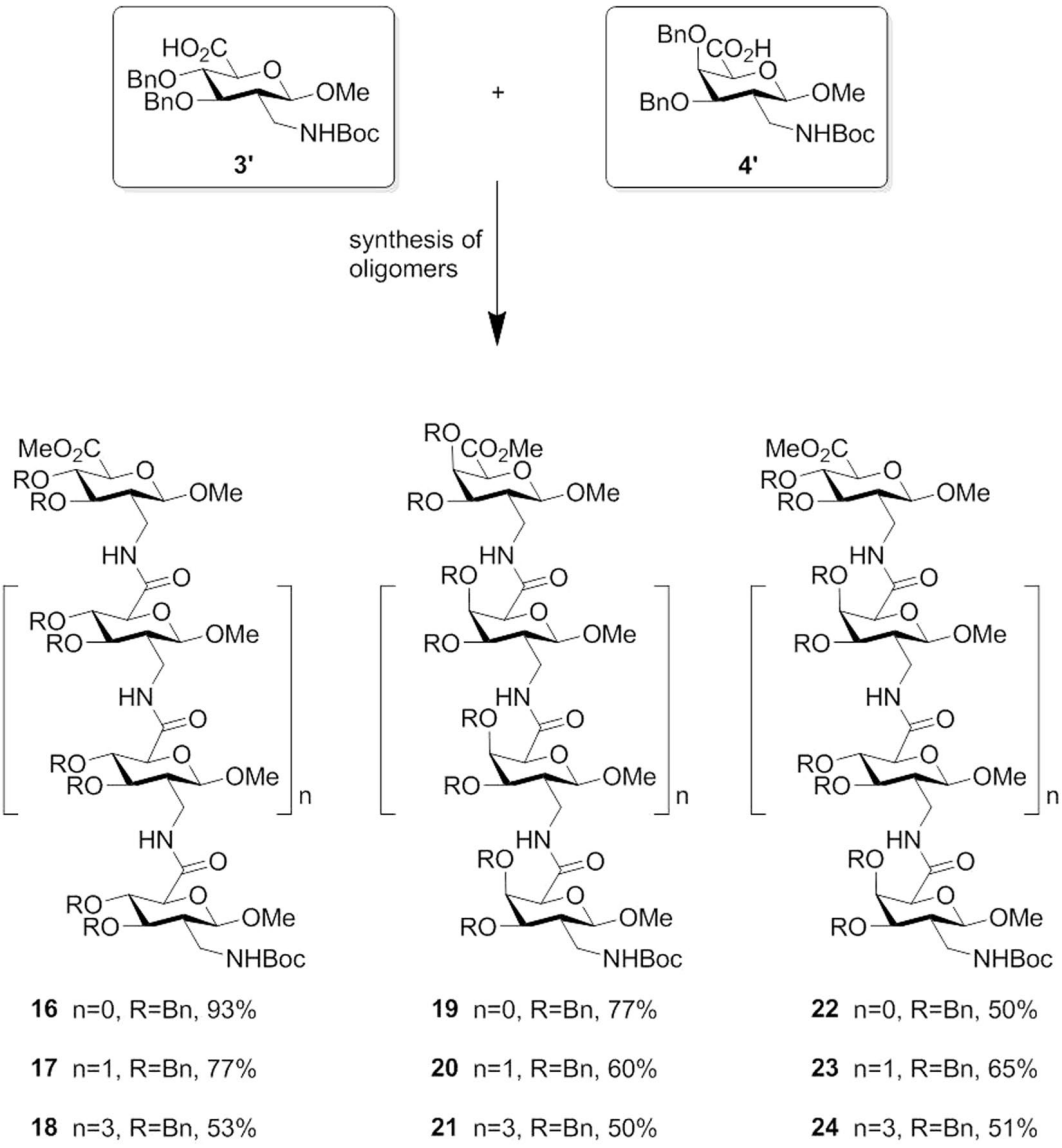
The synthetic linear homooligomers **16–21** and heterooligomers **22–24** with standard coupling method.

Sugar amino acids and their assembled oligomers, known as carbopeptoids, are commonly studied as foldamers to discover the structural consequences^16–36^. Accordingly, glycosaminuronic acid analogues **3′** and **4′** served as versatile scaffolds in construction of di-, tetra- and octamers. The free amino group of SAAs building blocks were exposed by using trifluoroacetic acid. Dimers (**16**, **19** and **22**) (Fig. 4) were synthesized based on aminuronic acids **3′** and **4′**, and tetramers (**17**, **20** and **23**) (Fig. 4) were achieved with coupling of dimers. Finally, octamers (**18**, **21** and **24**) (Fig. 4) were assembled by using tetramers. The standard coupling reaction procedure, achieved in the presence of diphenylphosphoryl azide (detailed synthesis see Electronic Supplementary Information), afforded nine oligomers in moderate to good yields (disaccharides 50–93%; tetrasaccharides 60–77%, octasaccharides 51–53%, see Electronic Supplementary Information). Obviously, the coupling efficiency varied with the prolongation of oligomers length as usually, while the assembly of homooligomers was superior to heterooligomers.

### Conformational analysis of oligo-SAAs by circular dichroism (CD) spectroscopy

In the ordinary way, study of the conformational preference of oligo-SAAs is often challenging with nuclear magnetic resonance (NMR) and IR Spectroscopy technologies. Thus, the known circular dichroism (CD) spectroscopy technology was taken to carry out the conformational discussion, because CD spectra can offer direct information on the similarity of conformational preference of the peptidomimetic backbone of different carbopeptoids owing to the phenomenon of exciton coupling, as evidenced for α-peptides and proteins^59–61^.

**Figure 5:**
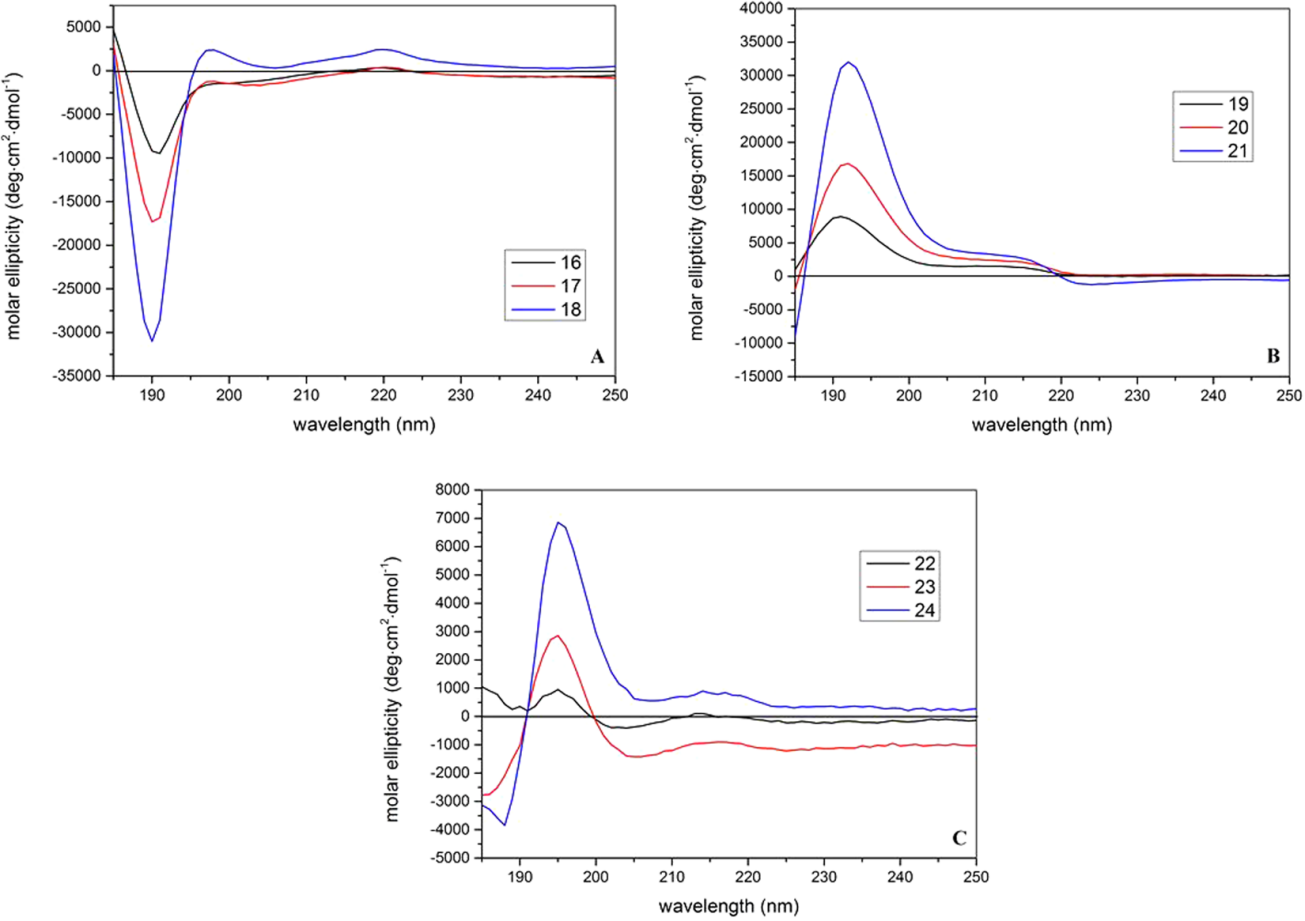
Normalized circular dichroism (CD) spectra of nine oligosaccharides (**16**–**24**) recorded in trifluoroethanol solution.

*Gluco*-homooligomers, *galacto*-homooligomers and *gluco*-*galacto*-heterooligomers form polyproline II (PPII) conformations but with opposite handedness. CD experiments were carried out to characterize the conformations of nine oligosaccharides (**16**–**24**). The magnitude of CD signals increase with the increase of oligomerization unit numbers. There are a large negative peak at 190 nm and two small positive ones at 197 nm and 220 nm respectively. The spectrum shapes resemble those of the poly-proline II (PPII) helices expect with slightly shifted peaks (large negative one at 195 nm and small positive one at 215 nm)^62^. These observations suggest that the *gluco*-homooligomers (Fig. 5A) might have a well-extended, left-handed conformation similar to PPII helices. The curve shapes of *galacto*-homooligomers (Fig. 5B) and *gluco*-*galacto*-heterooligomers (Fig. 5C) CD spectra are similar to those of gluco-form with the total opposite signs, which suggests a right-handed PPII conformation.

### Conformational analysis of oligo-SAAs by NMR spectroscopy

**Figure 6:**
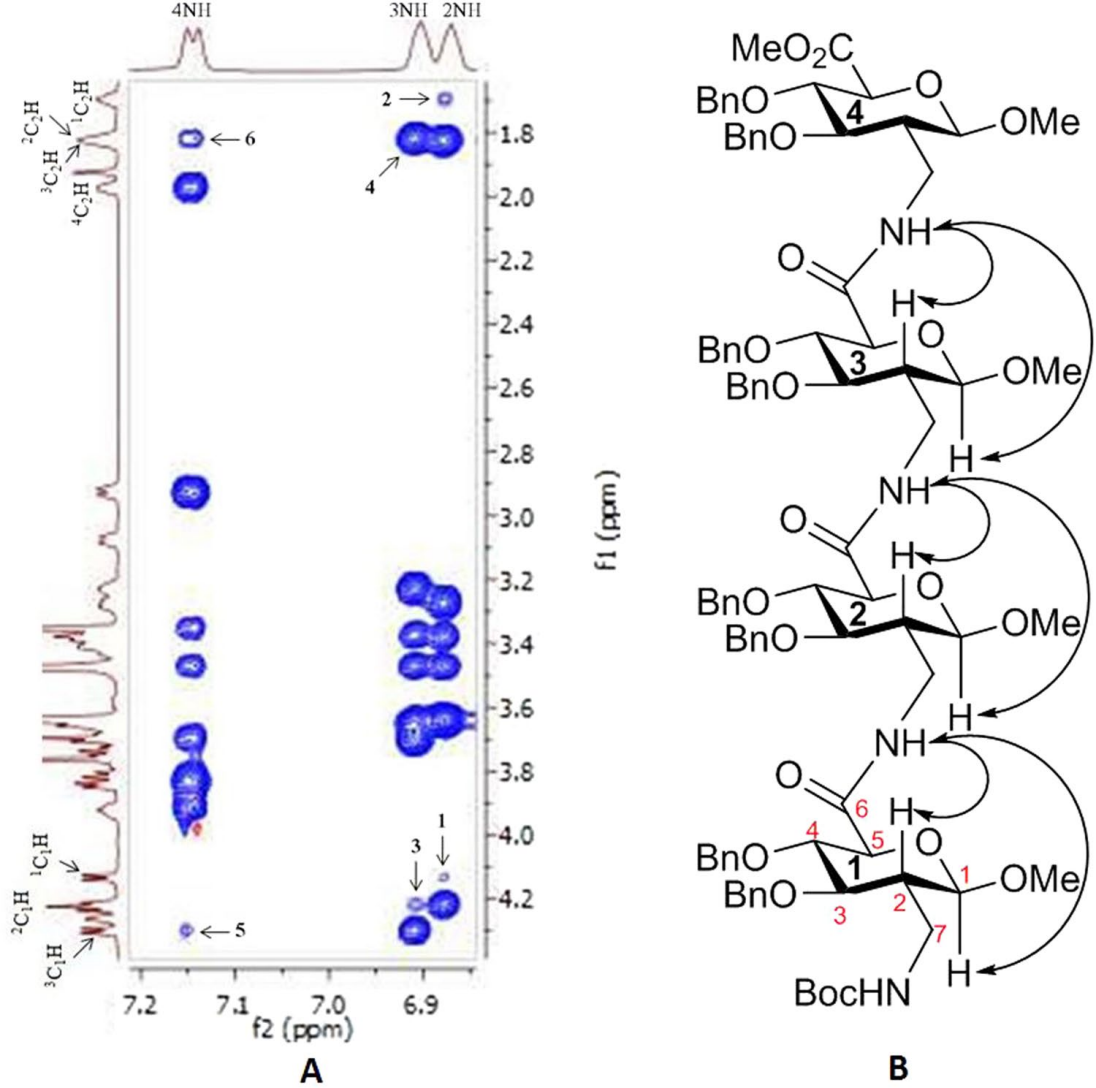
(**A**) Expanded ROESY spectrum of **17** in CDCl_3_ (ca. 10 mM, 263 K). The nOes ^2^NH ↔ ^1^C_1_H, ^1^C_2_H, ^3^NH ↔ ^2^C_1_H, ^2^C_2_H and ^4^NH ↔ ^3^C_1_H, ^3^C_2_H are marked as **1–6**; (**B**). Characteristic nOes of compound **17** in CDCl_3_.

**Figure 7:**
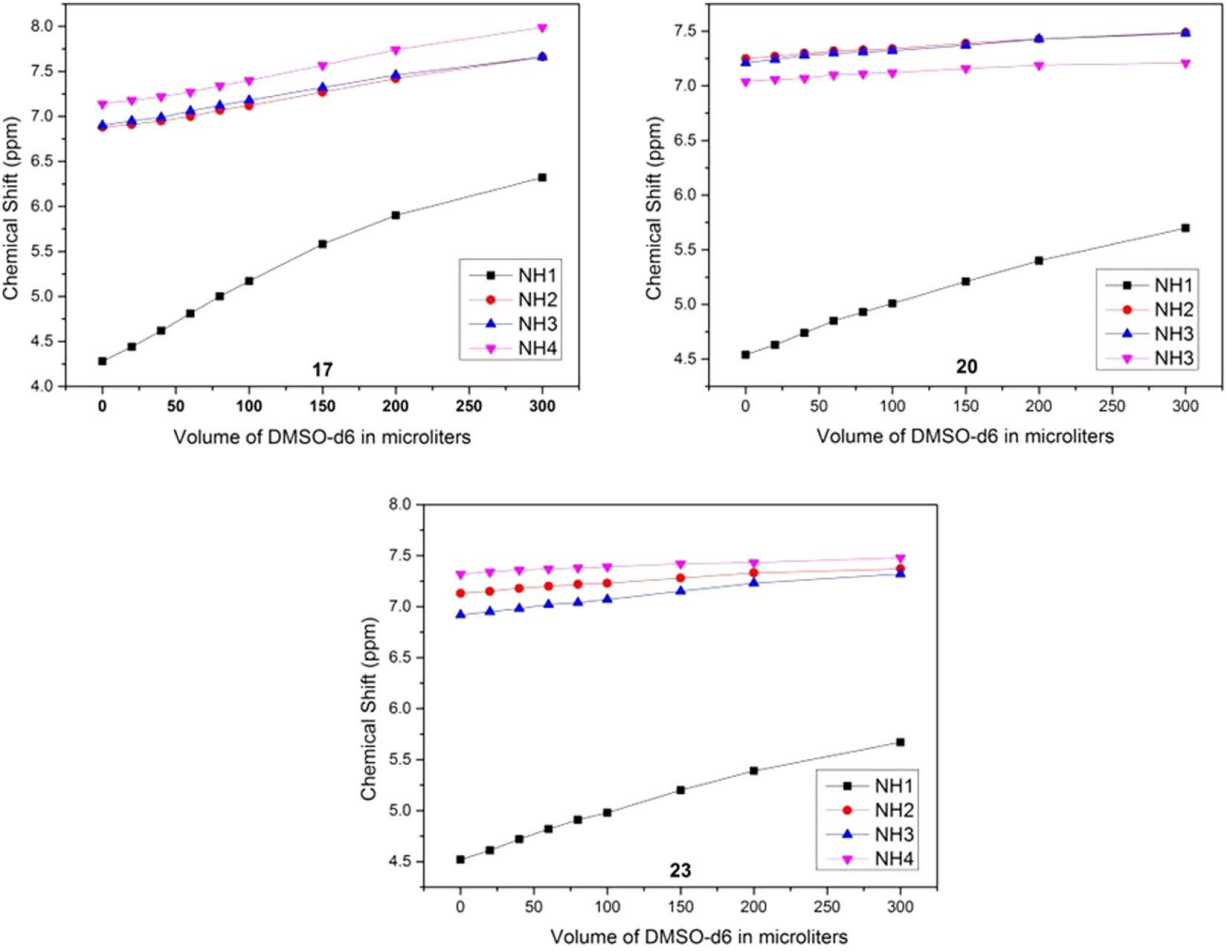
Solvent Titration plot of **17**, **20 and 23**.

To further support our conformational analysis, we chose three exemplary structures **17**, **20** and **23** and recorded NMR data to expand our insights with atomic resolution. Regular two-dimensional experiments (COSY, HSQC and TOCSY) were acquired for full resonance assignment in CDCl_3_ using TMS as a reference. The chemical shift dispersion of the amide resonances indicates the presence of ordered secondary structural elements. Next, 2D-ROESY spectra were acquired to identify ^**i+1**^**NH ↔ **^**i**^**C**_**1**_**H**, ^**i**^**C**_**2**_**H** correlations (Fig. 6 take compound **17** for example) which support our conformational analysis using CD spectroscopy for **17**, **20** and **23** (see supplementary information). Moreover, DMSO-d_6_ solvent titration experiments indicate the involvement of the amide protons NH2 to NH4 in hydrogen bond formation in three oligo-SAAs, while the solvent effect is stronger for NH1 suggesting the absence of hydrogen bonds in this position (Fig. 7). Taken together, NMR data acquired for compounds **17**, **20** and **23** clearly support our notion on the conformational preference derived from CD spectroscopy.

## Discussion

In conclusion, we have developed a rapid three-step entry to high yield novel *C*-branched sugar amino acids in analytically pure form from nitro compounds. For the first time, such 2-*C*-branched aminuronic acids, representing unnatural sialic acids, were obtained as biologically interesting structures. The novel sugar amino acids have been applied in assembly of nine carbopeptoids for the conformational elucidation by using CD experiments, which has also been further supported by the data from NMR spectroscopy. Much spectroscopic evidence has been recorded for this family of carbopeptoid, and computational studies will be performed for further investigations. The SAAs and their oligomers can be potential candidate scaffolds for discovering new drugs and materials.

## Methods

### General procedures for ZnCl_2_-mediated effectively direct 6-O-debenzylation

To a solution of 2-deoxy-2-*C*-nitromethyl-pyranoside (3.0 g, 6.0 mmol) in Ac_2_O/HOAc (2:1) (30 ml) was added a solution of freshly fused zinc chloride (ZnCl_2_) (7.5–10.0 eq) in Ac_2_O/HOAc (2:1) (30 ml), the mixture was stirred at room temperature for 2 h, TLC indicated that the reaction was complete. Water was added, and the mixture was extracted with DCM three times, washed with saturated NaHCO_3_, then water, dried over Na_2_SO_4_, and concentrated in vacuo. The crude mixture was purified by silica gel column chromatography to afford the pure product.

### General procedures (B) for assembly of sugar amino acids

To a stirred solution of **SAA** (1.0 equiv.) in dry DCM (reaction concentration is 0.13 M) at 0 °C was added trifluoroacetic acid (TFA) (1/3 V_DCM_) and the mixture was stirred until conversion of the starting material (monitored by TLC) at room temperature. The reaction mixture was then concentrated in *vacuo* to obtain the trifluoroacetate salt **SAA-a**.

A stirring solution of **SAA** (320 mg, 0.36 mmol) in THF/MeOH/H_2_O (2.1/0.7/0.7 mL) at 0 °C was added lithium hydroxide (LiOH·H_2_O) (50 mg, 1.06 mmol) and the mixture was stirred at room temperature for 1 h. The reaction mixture was then acidified to pH 2 with 1 N HCl. The reaction mixture was extracted with EtOAc (2 × 20 mL). The combined organic extracts were washed with water, brine, dried over Na_2_SO_4_, filtered and concentrated in vacuo to obtain the crude acid **SAA-b**, which was used for the next reaction without further purification.

To a stirring solution of the crude acid **SAA-b** in dry DMF (2/3 V, reaction concentration is 0.1 M) at 0 °C were sequentially added triethylamine (Et_3_N) (3.0 equiv.) and diphenyl azidophosphate (DPPA) (1.5 equiv.). After 10 min, the above prepared trifluoroacetate salt **SAA-a** was dissolved in DMF (1/3 V) and added to the reaction mixture. After stirring for 15 h at room temperature, the reaction mixture was diluted with DCM, washed with 1 N HCl solution, saturated NaHCO_3_ solution, water, brine, dried over Na_2_SO_4_, filtered and concentrated in *vacuo*. Purification by silica gel column chromatography afforded assembled oligomers.

### Circular Dichroism spectroscopy

CD spectra were recorded on Chirascan spectrometer at 25 °C in trifluoroethanol, using 10 mm path length CD cell. All spectra represented the average of 5 scans. They were all background corrected. The concentration of peptides used was 0.07 mM, Scan Range: 185–250 nm: band width: 2 nm.

## Electronic supplementary material

Supplementary Information
