# Supplementary material for: Synthesis and conformational analysis of linear homo- and heterooligomers from novel 2-C-branched sugar amino acids (SAAs)
Source: Sci Rep. 2018 Apr 26;8:6625. doi: 10.1038/s41598-018-24927-6 (PMC5919921; doi:10.1038/s41598-018-24927-6)
Supplement: Supplementary file 1 — Supplementary Information [file 41598_2018_24927_MOESM1_ESM.doc]

Electronic Supplementary Information

**Synthesis and conformational analysis of linear homo- and heterooligomers from novel 2-*C*-branched**

**sugar amino acids (SAAs)**

Guang-Zong Tian^a^, Jing Hu^*b^, Heng-Xi Zhang^c^, Rademacher Christoph^c^, Xiao-Peng Zou^a^, Hong-Ning Zheng^a^, Fei Xu^a^, Xiao-Li Wang^a^, Torsten Linker^d^ and Jian Yin^*a^

*^a^G Tian, X. Zou, Dr. H. Zheng, Prof. F. Xu, Dr. X. Wang, Prof. J. Yin*

*Key Laboratory of Carbohydrate Chemistry and Biotechnology, Ministry of Education, School of Biotechnology, Jiangnan University, Lihu Avenue 1800, Wuxi, Jiangsu, 214122 (P.R. China)*

*E-mail: jianyin@jiangnan.edu.cn*

*^b^Dr. J. Hu,*

*Wuxi School of Medicine, Jiangnan University Lihu Avenue 1800, Wuxi, Jiangsu, 214122 (P.R. China), E-mail:* [*hujing@jiangnan.edu.cn*](mailto:hujing@jiangnan.edu.cn)

*^c^H. Zhang, Dr. R. Christoph*

*Department of Biomolecular Systems, Max Planck Institute of Colloids and Interfaces, Am Mühlenberg 1,*

*14476, Germany*

*^d^Prof. T. Linker,*

*Department of Chemistry, University of Potsdam, Karl-Liebknecht-Str. 24–25, 14476 Potsdam (Germany)*

***Contents***

**1. General information S1**

**2. Experimental details and characterization data S1−S20**

**3. Conformational analysis of oligo-SAAs by NMR spectroscopy S21−S30**

**4. NMR spectra S31−S52**

**5. MALDI-TOF mass spectra S53**

**1. General information**

All the starting materials were purchased as reagent grade and used without further purification except where noted. Solvents were dried and redistilled prior to use in the usual way. All reactions were conducted in oven-dried glassware with magnetic stirring, under an argon atmosphere if need. Analytical thin layer chromatography (TLC) was performed on GF_254_ [aluminium](javascript:void(0);) plates precoated with a thickness of silica gel. The TLC plates were visualized with UV light and by staining with Hanessian solution (ceric sulfate and ammonium molybdate in aqueous sulfuric acid) or 5% sulfuric acid-ethanol solution. Column chromatography was performed on silica gel (200–300 mesh). Optical rotations were measured with an AUTOPOL IVS2 & PLUS & VI at a concentration (c) expressed in g/100 mL. IR spectra were taken with a NICOLET IS5 FT-IR spectrometer. ^1^H and ^13^C NMR, ^1^H-^1^H COSY spectra were measured with Bruker AVANCE III 400 MHz , Bruker AVANCE III 600 MHz and AVANCE III 700 MHz. Chemical shifts are in ppm from Me_4_Si, generated from the CDCl_3_ lock signal at 7.26, CD_3_OD lock signal at 3.31. Multiplicities are quoted as singlet (s), broad singlet (br s), doublet (d), doublet of doublets (dd), triplet (t), quartet (q) or multiplet (m). All NMR chemical shifts (δ) were recorded in ppm and coupling constants (J) were reported in Hz. High-resolution mass spectra (HRMS) were recorded with an FT-ICR-MS. MALDI-TOF spectra were recorded on a Bruker Daltonics ultrafleXtreme MALDI TOF/TOF, using NaCl/2,5-dihydroxy benzoic acid (DHB) as the matrix.

**2. Experimental details and characterization data**

**2.1 Synthesis of monomer building blocks**

**Methyl 2-deoxy-2-*C*-(*t*-butoxycarbonyl)amino-β-D-glucopyranoside (9)**

The 2-deoxy-2-*C*-nitromethyl-pyranoside **5** (4.10 g, 8 mmol) was dissolved in MeOH (50 mL) and palladium on carbon (10%) (0.96 g) was added. The reaction mixture was stirred in an autoclave under H_2_ atmosphere for 2 h and TLC showed complete conversion. The mixture was filtered over celite and concentrated to syrup. The crude product was dissolved in MeOH/H_2_O (3/1) (120 mL) and sodium hydroxide (0.64 g, 16 mmol) was added. To the solution was given di-tert-butyl dicarbonate (2.62 g, 12 mmol) at 0 ºC. The solution was stirred overnight at room temperature and TLC showed complete conversion. All solvent was evaporated and extracted with DCM (5×30 mL). The organic phase was washed with saturated brine, dried with anhydrous sodium sulfate (Na_2_SO_4_), and concentrated in vacuo to a residue. The crude product was purified by column chromatography ([dichloromethane](javascript:void(0);) / methanol = 20:1). Yield: (1.56 g, 64% over two steps).

R_f_ = 0.24 (dichloromethane /methanol = 10:1); [α]_D_^25^ = - 54.6 (c = 1.0 in CHCl_3_); ^1^H NMR (400 MHz, Chloroform-*d*) δ = 4.97 (t, *J* = 6.6 Hz, 1H, NH) , 4.23 (d, *J* = 8.7 Hz, 1H, 1-H), 3.91 (dd, *J*=12.4, 6.3 Hz, 1H, 6’-H), 3.84 (dd, *J* = 12.4, 3.4 Hz, 1H, 6-H), 3.55 (t, *J* = 9.8 Hz, 1H, 4-H), 3.51 (s, 3H, OMe), 3.52 – 3.44 (m, 1H, 7’-H), 3.42 – 3.34 (m, 2H, 5-H/3-H), 3.34 – 3.28 (m, 1H, 7-H), 1.64 – 1.52 (m, 1H, 2-H), 1.45 (s, 9H, Boc). ^13^C-NMR (101 MHz, Chloroform-*d*): δ = 157.9 (CO^Boc^), 102.4 (C-1), 80.5 (C- ^t^Bu), 75.6 (C-5), 72.4 (C-4), 71.4 (C-3), 62.9 (C-6), 57.0 (OMe), 48.7 (C-2), 37.1 (C-7), 28.5 (^t^Bu). IR (film): ν = 3388, 2977, 2933, 1694, 1514, 1367, 1251, 1171, 1079, 610 cm^-1^; HRMS (ESI) m/z calcd for C_13_H_25_O_7_NNa [M + Na]^+^ 330.1529, found 330.1528.

**Methyl 2-deoxy-2-*C*-(*t*-butoxycarbonyl)amino-6-*O*-TIPS-β-D-gluco-pyranoside (10)**

A mixture of compound **9** (0.80 g, 2.6 mmol), triisopropylchlorosilane (0.83 mL, 3.9 mmol), and imidazole (0.35 g, 5.2 mmol) in DMF (10 mL) was stirred at room temperature for 10 h under argon atmosphere. The mixture was poured into water and extracted twice with DCM. The organic phase was washed twice with 5% HCl, dried with anhydrous sodium sulfate (Na_2_SO_4_), and concentrated in vacuo to a residue and the crude product was purified by column chromatography ([petroleum](javascript:void(0);) [ether](javascript:void(0);) /ethyl acetate = 4:1). Yield: (1.07 g, 89%).

R_f_ = 0.30 (petroleum ether /ethyl acetate = 2:1); [α]_D_^25^ = - 20.9 (c = 1.0 in CHCl_3_); ^1^H NMR (400 MHz, Chloroform-*d*) δ = 4.90 (t, *J* = 6.6 Hz, 1H, NH), 4.19 (d, *J* = 8.6 Hz, 1H, 1-H), 3.94 – 3.82 (m, 2H, 6-H), 3.55 (dd, *J* = 9.2 Hz, 1H, 4-H), 3.57 – 3.49 (m, 1H, 7’-H), 3.48 (s, 3H, OMe), 3.42 (dd, *J* = 9.9 Hz, 1H, 3-H), 3.35 (dt, *J* = 8.9, 5.4 Hz, 1H, 5-H), 3.37 – 3.28 (m, 1H, 7-H), 1.64 – 1.52 (m, 1H, 2-H), 1.45 (s, 9 H, Boc), 1.09 – 0.94 (m, 21H, TIPS); ^13^C-NMR (101 MHz, Chloroform-*d*): δ = 157.5 (CO^Boc^), 102.0 (C-1), 80.1 (C-^t^Bu), 74.8 (C-5), 73.3 (C-4), 72.5 (C-3), 65.2 (C-6), 56.6 (OMe), 48.1 (C-2), 37.1 (C-7), 28.4 (^t^Bu), 17.9 (CH_3_^TIPS^), 11.8 (C-Si^TIPS^); IR (film): ν = 3437, 2925, 2865, 1690, 1510, 1462, 1367, 1250, 1168, 1073, 1011, 883, 794, 683 cm^-1^; HRMS (ESI) m/z calcd for C_22_H_45_O_7_NSiNa [M + Na]^+^ 486.2863, found 486.2860.

**Methyl 3,4-di-*O*-benzyl-2-deoxy-2-*C*-(*t*-butoxycarbonyl)amino-6-*O*-TIPS-β-D-gluco-**

**pyranoside (11)**

Compound **10** (1.03 g, 2.2 mmol) was solved in absolute THF (8.5 mL) under argon atmosphere, 18-crown-6 (160 mg, 0.60 mmol), potassium hydroxide (0.50 g, 8.9 mmol) and after 15 min, benzylbromide (0.83 ml, 6.7 mmol) was added and the white suspension was stirred over 5 h under argon at room temperature. THF was evaporated, the residue was solved in DCM, which was washed with water and dried with anhydrous sodium sulfate (Na_2_SO_4_) and evaporated. The crude product was purified with column chromatography ([petroleum](javascript:void(0);) [ether](javascript:void(0);) /ethyl acetate = 18:1). Yield: (1.01 g, 71%).

R_f_ = 0.32 (petroleum ether /ethyl acetate = 9:1); [α]_D_^25^ = + 14.2 (c = 1.0 in CHCl_3_); ^1^H NMR (400 MHz, Chloroform-*d*) δ = 7.42 – 7.26 (m, 10H, arom. H), 4.90 (d, *J* = 10.69 Hz, 1H, CH_2_-Ph), 4.85 (d, *J* = 10.9 Hz, 1H, CH_2_-Ph), 4.81 (t, *J* = 6.6 Hz, 1H, NH), 4.78 (d, *J* = 10.8 Hz, 1H, CH_2_-Ph), 4.70 (d, *J* = 10.6 Hz, 1H, CH_2_-Ph), 4.13 (d, *J* = 8.6 Hz, 1H, 1-H), 3.98 (d, *J* = 2.8 Hz, 2H, 6-H), 3.74 (dd, *J* = 9.2 Hz, 1H, 4-H), 3.51 (dd, *J* = 13.4, 6.2 Hz, 1H, 7’-H), 3.47 (s, 3H, OMe), 3.41 (dd, *J* = 9.9 Hz, 1H, 3-H), 3.26 (dt, *J* = 9.6, 2.9 Hz, 1H, 5-H), 3.19 (dt, *J* = 13.0, 5.5 Hz, 1H, 7-H), 1.74 (tdd, *J* = 10.1, 6.2, 4.1 Hz, 1H, 2-H), 1.42 (s, 9 H, Boc), 1.20 – 0.90 (m, 21H, TIPS); ^13^C-NMR (101 MHz, Chloroform-*d*): δ = 155.8 (CO^Boc^), 138.3, 137.9 (arom. C-CH_2_O), 128.6, 128.5, 128.5, 128.0, 127.8, 127.8 (arom. C-H), 103.3 (C-1), 80.5 (C-4), 79.3 (C-5), 78.8 (C-^t^Bu), 76.1 (CH_2_-Ph), 75.0 (CH_2_-Ph), 74.7 (C-3), 62.4 (C-6), 56.4 (OMe), 47.6 (C-2), 38.4 (C-7), 28.4 (^t^Bu), 18.0 (CH_3_^TIPS^), 12.0 (C-Si^TIPS^); IR (film): ν = 2942, 2866, 1719, 1499, 1365, 1167, 1099, 698 cm^-1^; HRMS (ESI) m/z calcd for C_36_H_57_O_7_NSiNa [M + Na]^+^ 666.3802, found 666.3804.

**Methyl 3,4-di-*O*-benzyl-2-deoxy-2-*C*-(*t*-butoxycarbonyl)amino-β-D-glucopyranoside** **(12)**

Compound **11** (0.91 g, 1.4 mmol) was dissolved in THF (13.4 mL) and cooled to 0 °C. Tetrabutylammonium fluoride (TBAF) (3.4 mL, 3.4 mmol) was added to the substrate at 0 °C. The reaction was stirred at room termperature overnight. THF was evaporated, the residue was solved in DCM, which was washed with water and dried with anhydrous sodium sulfate (Na_2_SO_4_) and concentrated. The residue was purified by column chromatography ([petroleum](javascript:void(0);) [ether](javascript:void(0);) /ethyl acetate = 5:1-2:1). Yield: (0.67 g, 98%).

R_f_ = 0.33 (petroleum ether /ethyl acetate = 1:1); [α]_D_^25^ = + 12.3 (c = 1.0 in CHCl_3_); ^1^H NMR (400 MHz, Chloroform-*d*) δ = 7.46 – 7.42 (m, 10H, arom. H), 4.90 (d, *J* = 11.0 Hz, 1H, CH_2_-Ph), 4.86 (d, *J* = 11.0 Hz, 1H, CH_2_-Ph), 4.72 (d, *J* = 11.4 Hz, 1H, CH_2_-Ph), 4.70 (d, *J* = 11.0 Hz, 1H, CH_2_-Ph), 4.57 (s, 1H, NH), 4.18 (d, *J* = 8.7 Hz, 1H, 1-H), 3.89 (dd, *J* = 12.0, 2.6 Hz, 1H, 6’-H), 3.76 (dd, *J* = 11.9, 4.2 Hz, 1H, 6-H), 3.63 (dd, *J* = 9.2 Hz, 1H, 4-H), 3.52 – 3.42 (m, 1H, 7’-H), 3.51 (s, 3H, OMe), 3.45 (t, *J* = 9.8 Hz, 1H, 3-H), 3.37 – 3.29 (m, 1H, 5-H), 3.24 – 3.12 (m, 1H, 7-H), 1.98 (s, 1H, 6-OH), 1.72 (tt, *J* = 9.5, 5.0 Hz, 1H, 2-H), 1.41 (s, 9H, Boc); ^13^C-NMR (101 MHz, Chloroform-*d*): δ = 155.9 (CO^Boc^), 138.0, 137.9 (arom. C-CH_2_O), 128.8, 128.7, 128.6, 128.3, 128.1, 128.0 (arom. C-H), 103.6 (C-1), 79.9 (C-4), 79.6 (C-5), 79.1 (C- ^t^Bu), 75.29 (CH_2_-Ph), 75.0 (CH_2_-Ph), 74.9 (C-3), 62.0 (C-6), 57.0 (OMe), 47.9 (C-2), 37.7 (C-7), 28.6 (^t^Bu); IR (film): ν = 3429, 2976, 2932, 1694, 1509, 1454, 1367, 1251, 1169, 1092, 1027, 698 cm^-1^; HRMS (ESI) m/z calcd for C_27_H_37_O_7_NNa [M + Na]^+^ 510.2468, found 510.2464.

**Methyl 3,4-di-*O*-benzyl-2-deoxy-2-*C*-(*t*-butoxycarbonyl)amino-β-D-glucuronic acid (3’)**

Compound **12** (0.67 g, 1.4 mmol) was solved in DCM (5.0 mL) and cooled in an ice bath. 2,2,6,6-tetramethylpiperidine 1-oxyl radical (TEMPO, 48 mg, 0.30 mmol) was added and afterwards a mixed solution of saturated solution NaHCO_3_ in water, sodium bromide (NaBr, 50 mg, 0.34 mmol), tetrabutylammonium bromide (TBABr, 63 mg, 0.23 mmol). While cooling, a solution of sodium hypochlorite (13% NaClO, 2.0 mL), saturated solution of NaHCO_3_ (1.3 mL), and brine (2.7 mL) was added so that the pH value never exceeds pH 10. After 75 min, the reaction was finished (TLC control). The solution was diluted with dichloromethane and water for better separation of the layers. The aqueous layer was acidified with Amberlite IR 120, extracted several times with ethylacetate. The ethylacetate layer was combined with the DCM layer, dried with with anhydrous sodium sulfate (Na_2_SO_4_), evaporated and the residue purified by column chromatography ([dichloromethane](javascript:void(0);) /methanol = 100:1). Yield: (0.67 g, quan.).

R_f_ = 0.25 (dichloromethane /methanol = 10:1); [α]_D_^25^ = + 11.6 (c = 1.0 in CHCl_3_); ^1^H NMR (400 MHz, Chloroform-*d*) δ = 9.58 (br s, 1H, COOH), 7.44 – 7.17 (m, 10H, arom. H), 4.81 (d, *J* = 10.7 Hz, 1H, Ph-CH_2_), 4.78 (d, *J* = 10.3 Hz, 1H, Ph-CH_2_), 4.67 (d, *J* = 10.8 Hz, 1H, Ph-CH_2_), 4.62 (d, *J* = 11.0 Hz, 1H, Ph-CH_2_), 4.51 (t, *J* = 6.2 Hz, 1H, NH), 4.21 (d, *J* = 8.6 Hz, 1H, 1-H), 3.92 (d, *J* = 8.7 Hz, 1H, 5-H), 3.84 (t, *J* = 8.4 Hz, 1H, 4-H), 3.47 (s, 3H, OMe), 3.50 – 3.37 (m, 2H, 3-H/7’-H), 3.27 – 3.08 (m, 1H, 7-H), 1.75 (tt, *J* = 9.7, 4.9 Hz, 1H, 2-H), 1.41 (s, 9H, Boc); ^13^C-NMR (101 MHz, Chloroform-*d*) δ = 173.0 (C-6), 155.9 (CO^Boc^), 138.0 , 137.9 (arom. C-CH_2_O), 128.6, 128.4, 128.4, 128.1, 128.0, 127.7 (arom. C-H), 103.4 (C-1), 81.3 (C-4), 79.0 (CH_2_-Ph), 78.7 (C-^t^Bu), 76.0 (C-5), 74.3 (CH_2_-Ph), 74.1 (C-3), 57.2 (OMe), 47.2 (C-2), 34.5 (C-7), 28.4 (^t^Bu); IR (film): ν = 3430, 2926, 1716, 1499, 1454, 1392, 1365, 1247, 1212, 1171, 1072, 1029, 752, 699 cm^-1^; HRMS (ESI) m/z calcd for C_27_H_35_O_8_NNa [M + Na]^+^ 524.2260, found 524.2270.

**Methyl 3,4-di-*O*-benzyl-2-deoxy-2-*C*-(*t*-butoxycarbonyl)amino-β-D-glucuronic acid methyl ester (3a)**

Compound **3’** (280 mg, 0.57 mmol) was solved in DMF (2 mL) and sodium bicarbonate (NaHCO_3_) (72 mg, 0.86 mmol) was added and afterwards methyl iodide (CH_3_I) (70 uL, 1.14 mmol). DMF was evaporated, the residue was solved in DCM, which was washed with water, brine and dried with anhydrous sodium sulfate (Na_2_SO_4_) and concentrated. The residue was purified by column chromatography ([petroleum](javascript:void(0);) [ether](javascript:void(0);) /ethyl acetate = 6:1). Yield: (290 mg, quan.).

R_f_ = 0.28 (petroleum ether /ethyl acetate = 3:1); [α]_D_^25^ = + 20.6 (c = 1.0 in CHCl_3_); ^1^H NMR (400 MHz, Chloroform-*d*) δ = 7.22 – 7.41 (m, 10H, arom. H), 4.87 (d, *J* = 11.0 Hz, 1H, CH_2_-Ph), 4.77 (d, *J* = 10.8 Hz, 1H, CH_2_-Ph), 4.70 (d, *J* = 11.0 Hz, 1H, CH_2_-Ph), 4.62 (d, *J* = 10.8 Hz, 1H, CH_2_-Ph), 4.44 (s, 1H, NH), 4.20 (d, *J* = 8.5 Hz, 1H, 1-H), 3.91 – 3.84 (m, 2H, 5-H/4-H), 3.75 (s, 3H, COOMe), 3.50 (s, 3H, OMe), 3.48 – 3.39 (m, 2H, 7’-H/3-H), 3.15 (dd, *J* = 13.7, 6.8 Hz, 1H, 7-H), 1.85 – 1.76 (m, 1H, 2-H), 1.41 (s, 9H, Boc); ^13^C-NMR (101 MHz, Chloroform-*d*): δ = 169.5 (C-6), 155.9 (CO^Boc^), 137.8, 137.7 (arom. C-CH_2_O), 128.8, 128.7, 128.7, 128.3, 128.1 (arom. C-H), 103.9 (C-1), 81.3 (C-4), 79.9 (CH_2_-Ph), 79.2 (C-^t^Bu), 78.9 (C-5), 74.8 (CH_2_-Ph), 74.7 (C-3), 57.4 (OMe), 52.7 (COOMe), 47.4 (C-2), 39.0 (C-7), 28.5 (^t^Bu); IR (film): ν = 3396, 2923, 2853, 1742, 1713, 1527, 1454, 1366, 1248, 1156, 1083, 1070, 735, 695 cm^-1^; HRMS (ESI) m/z calcd for C_28_H_37_O_8_NNa [M + Na]^+^ 538.2417, found 538.2411.

**Methyl 2-deoxy-2-*C*-(*t*-butoxycarbonyl)amino-β-D-glucuronic acid methyl ester (3)**

Compound **3a** (100 mg, 0.19 mmol) was dissolved in MeOH (2 mL) and palladium on carbon (10%) (50 mg) was added. The reaction mixture was stirred under H_2_ atmosphere for over night and TLC showed complete conversion. The mixture was filtered over celite and concentrated to syrup. The residue was purified by column chromatography (dichloromethane /methano = 40:1). Yield: (63 mg, quan.).

R_f_ = 0.36 (dichloromethane /methanol = 20:1); [α]_D_^25^ = -42.9 (c = 1.0 in CHCl_3_); ^1^H NMR (400 MHz, Chloroform-*d*) δ = 4.91 – 4.84 (m, 1H, NH), 4.24 (d, *J* = 8.8 Hz, 1H, 1-H), 3.84 (s, 3H, COOMe), 3.83 (d, *J* = 9.6 Hz, 1H, 5-H), 3.76 (t, *J* = 9.1 Hz, 1H, 4-H), 3.58 – 3.52 (m, 1H, 7’-H), 3.52 (s, 3H, OMe), 3.43 (dd, *J* = 10.8, 8.5 Hz, 1H, 3-H), 3.31 (ddd, *J* = 14.6, 6.0, 2.9 Hz, 1H, 7-H), 1.71 – 1.64 (m, 1H, 2-H), 1.45 (s, 9H, Boc); ^13^C NMR (101 MHz, Chloroform-*d*) δ = 169.8, 157.7, 102.6, 80.4, 74.8, 71.9, 71.7, 57.1, 52.7, 48.1, 36.9, 28.3; IR (film): ν = 3383, 2919, 1747, 1689, 1517, 1250, 1171, 1060 cm^-1^; HRMS (ESI) m/z calcd for C_14_H_25_NO_8_Na [M + Na]^+^ 358.1478, found 358.1476.

**Methyl 3,4-di-*O*-benzyl-6-*O*-acetyl-2-deoxy-2-*C*-nitromethyl-β-D-glucopyranoside (13)**

To a solution of 2-deoxy-2-*C*-nitromethyl-pyranoside **5** (3.0 g, 6.0 mmol) in Ac_2_O/HOAc (2:1) (30 ml) was added a solution of freshly fused zinc chloride (ZnCl_2_) (7.5-10.0 eq) in Ac_2_O/HOAc (2:1) (30 ml), the mixture was stirred at room temperature for 2h, TLC indicated that the reaction was complete. Water was added, and the mixture was extracted with DCM three times, washed with saturated sodium carbonate, then water, dried with Na_2_SO_4_, and concentrated to give syrup. Purification of the syrup by column chromatography (petroleum ether /ethyl acetate = 10:1-8:1-6:1). Yield: (2.3 g, 85%).

R_f_ = 0.25 (petroleum ether /ethyl acetate = 4:1); [α]_D_^25^ = + 11.9 (c = 0.1 in CHCl_3_); ^1^H NMR (400 MHz, Chloroform-*d*) δ = 7.39 – 7.27 (m, 10H, arom. H), 4.94 (d, *J* = 10.9 Hz, 1H, CH_2_-Ph), 4.85 (d, *J* = 10.8 Hz, 1H, CH_2_-Ph), 4.66 (d, *J* = 11.0 Hz, 1H, CH_2_-Ph), 4.61 (d, *J* = 10.9 Hz, 1H, CH_2_-Ph), 4.57 (d, *J* = 4.4 Hz, 2H, 7-H), 4.42 (d, *J* = 8.6 Hz, 1H, 1-H), 4.39 (dd, *J* = 12.0, 2.2 Hz, 1H, 6’-H), 4.26 (dd, *J* = 12.0, 4.6 Hz, 1H, 6-H), 3.69 (dd, *J* = 11.0, 8.4 Hz, 1H, 3-H), 3.61 (t, *J* = 8.9 Hz, 1H, 4-H), 3.53 (ddd, *J* = 9.6, 4.7, 2.3 Hz, 1H, 5-H), 2.51 (s, 3H, OMe), 2.26 (ddt, *J* = 11.0, 8.7, 4.3 Hz, 1H, 2-H), 2.07 (s, 3H, OAc); ^13^C NMR (101 MHz, Chloroform-*d*) δ = 170.7 (CO), 137.5, 137.3 (arom. C-CH_2_O), 128.6, 128.2, 128.1, 128.0, 127.9 (arom. C-H), 101.4 (C-1), 79.8 (C-4), 79.2(C-5), 75.3, 75.0 (CH_2_-Ph), 73.1 (C-3), 71.9 (CH_2_-NO_2_), 62.9 (C-6), 57.3 (OMe), 46.6 (C-2), 20.9 (Ac); IR (film): ν = 3028, 2938, 1731, 1567, 1387, 1244, 1149, 1103, 1067, 1034, 737, 696 cm^-1^; HRMS (ESI) m/z calcd for C_24_H_29_NO_8_Na [M + Na]^+^ 482.1791, found 482.1785.

**New procedure for compound (12)**

Compound **13** (2.3 g, 5.1 mmol) was dissolved in THF (36 mL) and lithium aluminum hydride (LiAlH_4_, 770 mg, 20.4 mmol) was added to the solution under an argon atmosphere. The mixture was heated under reflux for 2h until TLC showed complete convertion. The mixture was filtered over celite and concentrated to syrup. The crude product was dissolved in MeOH/H_2_O (3/1) (80 mL) and sodium hydroxide (NaOH) (0.41 g, 10.2 mmol) was added. To the solution was given di-tert-butyl dicarbonate (Boc_2_O) (1.67 g, 7.7 mmol) at 0 ºC. The solution was stirred overnight at room temperature and TLC showed complete conversion. All solvent was evaporated and the crude product was purified by column chromatography (petroleum ether /ethyl acetate = 2:1). Yield: (1.6 g, 64% over two steps).

**Methyl 3,4-di-*O*-benzyl-6-*O*-acetyl-2-deoxy-2-*C*-nitromethyl-β-D-galactopyranoside (14)**

Compound **14** was synthesized following same procedure as described for the synthesis of **13**.

Yield: (3.7 g, 77%); R_f_ = 0.28 (petroleum ether /ethyl acetate = 3:1); [α]_D_^25^ = - 4.9 (c = 1.0 in CHCl_3_); ^1^H NMR (400 MHz, Chloroform-*d*) δ = 7.42 – 7.24 (m, 10H, arom. H), 4.90 (d, *J* = 11.6 Hz, 1H, CH_2_-Ph), 4.76 (d, *J* = 10.9 Hz, 1H, CH_2_-Ph), 4.72 (dd, *J* = 12.7, 4.3 Hz, 1H, 7’-H), 4.69 (dd, *J* = 12.7, 3.9 Hz, 1H, 7-H), 4.60 (d, *J* = 11.5 Hz, 1H, CH_2_-Ph), 4.50 (d, *J* = 10.9 Hz, 1H, CH_2_-Ph), 4.42 (d, *J* = 8.7 Hz, 1H, 1-H), 4.26 (dd, *J* = 11.2, 6.5 Hz, 1H, 6’-H), 4.14 (dd, *J* = 11.2, 6.3 Hz, 1H, 6-H), 3.90 – 3.87 (m, 1H, 4-H), 3.64 (dd, *J* = 11.5, 2.5 Hz, 1H, 3-H), 3.56 (td, *J* = 6.4, 1.1 Hz, 1H, 5-H), 3.50 (s, 3H, OMe), 2.73 (dddd, *J* = 12.7, 8.6, 4.3 Hz, 1H, 2-H), 2.00 (s, 3H, OAc); ^13^C NMR (100 MHz, Chloroform-d) δ = 170.5 (CO), 137.9, 137.0 (arom. C-CH_2_O), 128.7, 128.4, 128.2, 128.1, 127.9 (arom. C-H), 101.4 (C-1), 78.1 (C-4), 74.3 (C-5), 72.2, 72.1 (CH_2_-Ph), 72.0 (C-3), 70.0 (CH_2_-NO_2_), 63.1 (C-6), 57.0 (OMe), 41.8 (C-2), 20.8 (Ac); IR (film): ν = 3032, 2942, 2906, 2881, 1738, 1567, 1389, 1248, 1092, 1052, 736, 696 cm^-1^; HRMS (ESI) m/z calcd for C_24_H_29_NO_8_Na [M + Na]^+^ 482.1791, found 482.1780.

**Methyl 3,4-di-*O*-benzyl-2-deoxy-2-*C*-(*t*-butoxycarbonyl)amino-β-D-galactopyranoside (15)**

Yield: (1.44 g, 46%); R_f_ = 0.31 (petroleum ether /ethyl acetate = 1:2); [α]_D_^25^ = + 12.6 (c = 1.0 in CHCl_3_); ^1^H NMR (400 MHz, Chloroform-*d*) δ = 7.44 – 7.27 (m, 10H, arom. H), 4.92 (d, *J* = 11.8 Hz, 1H, CH_2_-Ph), 4.75 (d, *J* = 11.5 Hz, 1H, CH_2_-Ph), 4.74 (m, 1H, NH), 4.49 (d, *J* = 11.4 Hz, 1H, CH_2_-Ph), 4.61 (d, *J* = 11.9 Hz, 1H, CH_2_-Ph), 4.11 (d, *J* = 8.7 Hz, 1H, 1-H), 3.86 – 3.84 (m, 1H, 4-H), 3.84 – 3.78 (m, 1H, 6’-H), 3.61 – 3.53 (m, 1H, 7’-H/6-H), 3.49 (s, 3H, OMe), 3.34 (ddd, *J* = 7.6, 5.8, 2.4 Hz, 1H, 5-H), 3.21 (dd, *J* = 11.3, 2.6 Hz, 1H, 3-H), 3.20 (dt, *J* = 12.7, 5.4 Hz, 1H), 7-H), 2.21 (dddd, *J* = 11.7, 5.5, 3.8 Hz, 1H, 2-H), 1.61 (dd, *J* = 8.6, 4.0 Hz, 1H, 6-OH), 1.42 (s, 9H, Boc); ^13^C-NMR (101 MHz, Chloroform-*d*): δ = 156.0 (CO^Boc^), 138.4, 137.3 (arom. C-CH_2_O), 128.9, 128.6, 128.6, 128.5, 128.4, 128.1 (arom. C-H), 104.0 (C-1), 79.2 (C-4), 78.9 (C-5), 74.7 (C- ^t^Bu), 74.0 (CH_2_-Ph), 71.5 (CH_2_-Ph), 69.5 (C-3), 62.4 (C-6), 57.0 (OMe), 42.7 (C-2), 38.3 (C-7), 28.6 (^t^Bu); IR (film): ν = 3506, 3345, 2976, 2924, 2887, 1691, 1537, 1366, 1251, 1158, 1094, 1065, 1052, 699 cm^-1^; HRMS (ESI) m/z calcd for C_27_H_37_O_7_NNa [M + Na]^+^ 510.2468, found 510.2459.

**Methyl 3,4-di-*O*-benzyl-2-deoxy-2-*C*-(*t*-butoxycarbonyl)amino-β-D-galacturonic acid (4’)**

Yield: (1.46 g, quan.); R_f_ = 0.30 (dichloromethane /methanol = 8:1); [α]_D_^25^ = + 57.5 (c = 1.0 in CHCl_3_); ^1^H NMR (400 MHz, Chloroform-*d*) δ = 8.49 (s, 1H, COOH), 7.47 – 7.07 (m, 10H, arom. H), 4.82 (d, *J* = 10.9 Hz, 1H, Ph-CH_2_), 4.70 (d, *J* = 10.9 Hz, 1H, Ph-CH_2_), 4.67 (d, *J* = 12.5 Hz, 1H, Ph-CH_2_), 4.52 (t, *J* = 6.2 Hz, 1H, NH), 4.49 (t, *J* = 1.8 Hz, 1H, 4-H), 4.38 (d, *J* = 11.7 Hz, 1H, Ph-CH_2_), 4.09 (d, *J* = 8.8 Hz, 1H, 1-H), 3.97 (s, 1H, 5-H), 3.53 – 3.43 (m, 1H, 7-H), 3.49 (s, 3H, OMe), 3.34 (dd, *J* = 11.3, 2.7 Hz, 1H, 3-H), 3.15 (dt, *J* = 11.4, 4.9 Hz, 1H, 7-H’), 2.07 (ddt, *J* = 12.7, 9.3, 4.6 Hz, 1H, 2-H), 1.41 (s, 9H, Boc); ^13^C NMR (101 MHz, Chloroform-*d*) δ = 172.1 (C-6), 155.9 (CO^Boc^), 138.3, 137.2 (arom. C-CH_2_O), 128.7, 128.4, 128.3, 128.2, 128.2, 127.6 (arom. C-H), 103.6 (C-1), 78.9 (C-4), 77.6 (C- ^t^Bu), 74.7 (C-5), 74.4 (CH_2_-Ph), 72.5 (CH_2_-Ph), 70.7 (C-3), 57.4 (OMe), 42.2 (C-2), 34.5 (C-7), 28.4 (^t^Bu); IR (film): ν = 3430, 2976, 2932, 2869, 1716, 1499, 1366, 1246, 1167, 1093, 1042, 737, 699 cm^-1^; HRMS (ESI) m/z calcd for C_27_H_35_O_8_NNa [M + Na]^+^ 524.2260, found 524.2272.

**Methyl 3,4-di-*O*-benzyl-2-deoxy-2-*C*-(*t*-butoxycarbonyl)amino-β-D-galaturonic acid methyl ester (4a)**

Yield: (600 mg, 86%); R_f_ = 0.43 (petroleum ether /ethyl acetate = 1:1); [α]_D_^25^ = + 33.1 (c = 0.1 in CHCl_3_); ^1^H NMR (400 MHz, Chloroform-*d*) δ = 7.51 – 7.18 (m, 10H, arom. H), 4.8 (d, *J* = 11.74 Hz, 1 H, CH_2_-Ph), 4.72 (d, *J* = 11.5 Hz, 1H, CH_2_-Ph), 4.64 (s, 1H, NH), 4.63 (d, *J* = 11.8 Hz, 1 H, CH_2_-Ph), 4.45 (d, *J* = 11.5 Hz, 1H, CH_2_-Ph), 4.30 (dd, *J* = 2.6, 1.3 Hz, 1H, 4-H), 4.10 (d, *J* = 8.7 Hz, 1H, 1-H), 3.98 (d, *J* =1.3 Hz, 1H, 5-H), 3.70 (s, 3H, COOMe), 3.54 (s, 3H, OMe), 3.60 – 3.49 (m, 1H, 7’-H), 3.35 (dd, *J* = 11.4, 2.5 Hz, 1H, 3-H), 3.27 – 3.07 (m, 1H, 7-H), 2.26 (dddd, *J* = 12.2, 9.2, 5.6, 3.8 Hz, 1H, 2-H), 1.42 (s, 9H, Boc); ^13^C-NMR (101 MHz, Chloroform-*d*): δ = 169.2 (C-6), 156.0 (CO^Boc^), 138.2, 137.2 (arom. C-CH_2_O), 128.9, 128.5, 128.5, 128.3, 128.1, 127.8 (arom. C-H), 103.8 (C-1), 79.1 (C-4), 78.2 (C- ^t^Bu), 74.3 (C-5), 74.2 (CH_2_-Ph), 71.7 (CH_2_-Ph), 71.3 (C-3), 57.3 (OMe), 52.6 (COOMe), 42.1 (C-2), 38.1 (C-7), 28.5 (^t^Bu); IR (film): ν = 3370, 2973, 2949, 2921, 1766, 1708, 1533, 1249, 1207, 1160, 1105, 1048, 1031, 744, 697 cm^-1^; HRMS (ESI) m/z calcd for C_28_H_37_O_8_NNa [M + Na]^+^ 538.2417, found 538.2418.

**Methyl 2-deoxy-2-*C*-(*t*-butoxycarbonyl)amino-β-D-galaturonic acid methyl ester (4)**

R_f_ = 0.31 (dichloromethane /methanol = 20:1); [α]_D_^25^ = -57.5 (c = 1.0 in CHCl_3_); ^1^H NMR (400 MHz, Chloroform-*d*) δ = 5.08 (t, *J* = 6.1 Hz, 1H, NH), 4.75 (d, *J* = 2.9 Hz, 1H, 4-H), 4.42 (s, 1H, 5-H), 4.22 (d, *J* = 8.7 Hz, 1H), 4.17 – 4.10 (m, 1H, 3-H), 3.83 (s, 3H, COOMe), 3.52 – 3.40 (m, 2H), 3.34 (s, 3H, OMe), 3.33 – 3.19 (m, 2H, 7-H), 2.31 – 2.22 (m, 1H, 2-H), 1.4 (s, 9H, Boc); ^13^C NMR (101 MHz, Chloroform-*d*) δ = 173.1, 156.6, 107.0, 84.3, 79.8, 73.6, 69.4, 55.3, 54.3, 52.7, 39.9, 28.4; IR (film): ν = 3370, 2929, 1744, 1689, 1526, 1367, 1276, 1252, 1168 cm^-1^; HRMS (ESI) m/z calcd for C_14_H_25_NO_8_Na [M + Na]^+^ 358.1478, found 358.1479.

**2.2 Synthesis of oligosaccharide mimetics**

**Synthesis of compound (16)**

To a stirred solution of **3a** (380 mg, 0.74 mmol) in dry DCM (6 mL) at 0 °C was added trifluoroacetic acid (TFA) (1.8 mL) and the mixture was stirred for 2 h at room temperature. The mixture was quenched with triethylamine (Et_3_N) and then concentrated in vacuo to obtain the trifluoroacetate salt **3b**.

A stirring solution of **3’** (425 mg, 0.85 mmol) in dry DMF (4 mL) at 0°C were sequentially added triethylamine (Et_3_N) (310 μL, 2.21 mmol) and diphenyl azidophosphate (DPPA) (240 μL, 1.11 mmol). After 10 min, the above prepared trifluoroacetate salt **3b** was dissolved in DMF (3 mL) and added to the reaction mixture. After stirring for 12 h at room temperature, the reaction mixture was diluted with DCM, washed with 1 N HCl solution, saturated NaHCO_3_ solution, water, brine, dried (Na_2_SO_4_), filtered and concentrated in vacuo. Purification by silica gel column chromatography ([petroleum](javascript:void(0);) [ether](javascript:void(0);) /ethyl acetate = 5:1-3:1-2:1) afforded compound **16**. Yield: (614 mg, 93%).

R_f_ = 0.43 (petroleum ether /ethyl acetate = 1:1); [α]_D_^25^ = + 11.2 (c = 1.0 in CHCl_3_); ^1^H NMR (600 MHz, Chloroform-*d*) δ = 7.48 – 7.25 (m, 20H, arom. H), 6.90 (t, *J* = 5.56 Hz, 1H, NHCO), 4.92 (d, *J* = 10.89 Hz, 1H, Ph-CH_2_), 4.84 (d, *J* = 11.25 Hz, 1H, Ph-CH_2_), 4.77 (d, *J* = 10.86 Hz, 1H, Ph-CH_2_), 4.74 (d, *J* = 11.01 Hz, 1H, Ph-CH_2_), 4.70 (d, *J* = 10.50 Hz, 1H, Ph-CH_2_ ), 4.66 (d, *J* = 10.36 Hz, 1H, Ph-CH_2_), 4.62 (d, *J* = 10.82 Hz, 1H, Ph-CH_2_), 4.46 (s, 1H, NHBoc), 4.26 (d, *J* = 8.88 Hz, 1H, 1-H, Res-I), 4.24 (d, *J* = 8.87 Hz, 1H, 1-H, Res-II), 3.83-3.95 (m, 1H, 4-H, Res-I), 3.88 (d, *J* = 7.82 Hz, 1H, 5-H, Res-I), 3.80 (d, *J* = 7.89 Hz, 1H, 5-H, Res-II), 3.77 (s, 3H, COOMe), 3.72 (t, *J* = 7.61 Hz, 1H, 4-H, Res-II), 3.74 – 3.66 (m, 1H, 7-H, Res-I), 3.51 (s, 3H, OMe), 3.50 (s, 3H, OMe), 3.46 (dd, *J* = 10.91, 8.39 Hz, 2H, 3-H, Res-I/Res-II), 3.49 – 3.38 (m, 1H, 7-H, Res-II), 3.35 – 3.28 (m, 1H, 7-H, Res-I), 3.14 (d, *J* = 13.60 Hz, 1H, 7-H, Res-II), 1.91 (dtd, *J* = 11.23, 7.53, 3.87 Hz, 1H, 2-H, Res-I), 1.63-1.81 (m, 1H, 2-H, Res-II), 1.44 (s, 9H, Boc); ^13^C NMR (151 MHz, Chloroform-*d*) δ = 169.3, 169.0, 155.9, 137.8, 137.8, 137.7, 137.6, 128.7, 128.7, 128.6, 128.6, 128.6, 128.2, 128.1, 128.1, 128.1, 128.0, 128.0, 104.3, 102.9, 81.4, 80.9, 80.0, 78.3, 75.7, 75.1, 74.9, 74.7, 74.4, 74.1, 57.3, 57.2, 52.7, 47.0, 46.8, 38.3, 37.3, 28.5; IR (film): ν = 3367, 2932, 1747, 1688, 1660, 1534, 1108, 1074, 749, 696 cm^-1^; HRMS (ESI) m/z calcd for C_50_H_62_N_2_O_13_Na [M + Na]^+^ 921.4150, found 921.4145.

**Synthesis of compound (17)**

Compound **16** (293 mg, 0.33 mmol) was dissolved in dry DCM (2.5 mL) at 0 °C，followed by addition of trifluoroacetic acid (TFA) (0.8 mL) and the mixture was stirred for 2 h at room temperature. The mixture was quenched with triethylamine (Et_3_N) and then concentrated in vacuo to obtain the crude trifluoroacetate salt **16a**.

A stirring solution of **16** (320 mg, 0.36 mmol) in THF/MeOH/H_2_O (2.1/0.7/0.7 mL) at 0 °C was added lithium hydroxide (LiOH·H_2_O) (50 mg, 1.06 mmol) and the mixture was stirred at room temperature for 1 h. The reaction mixture was then acidified to pH 2 with 1 N HCl. The reaction mixture was extracted with EtOAc (2×20 mL). The combined organic extracts were washed with water, brine, dried (Na_2_SO_4_), filtered and concentrated in vacuo to obtain the crude acid **16b**, which was used for the next reaction without further purification.

To a stirring solution of the crude acid **16b** in dry DMF (2 mL) at 0 °C were sequentially added triethylamine (Et_3_N) (140 μL, 0.98 mmol) and diphenyl azidophosphate (DPPA) (105 μL, 0.489 mmol). After 10 min, the above prepared trifluoroacetate salt **16a** was dissolved in DMF (1 mL) and added to the reaction mixture. After stirring for 15 h at room temperature, the reaction mixture was diluted with DCM, washed with 1 N HCl solution, saturated NaHCO_3_ solution, water, brine, dried (Na_2_SO_4_), filtered and concentrated in vacuo. Purification by silica gel column chromatography (dichloromethane /methanol = 200:1-100:1-50:1) afforded compound **17**. Yield: (316 mg, 77%).

R_f_ = 0.22 (dichloromethane /methanol = 50:1); [α]_D_^25^ = + 12.8 (c = 1.0 in CHCl_3_); ^1^H NMR (600 MHz, Chloroform-*d*) δ = 7.25 – 7.43 (m, 40H, arom. H), 6.88 (d, *J* = 6.3 Hz, 2H, NHCO), 7.08 (dd, *J* = 8.5, 2.8 Hz, 1H, NHCO) , 4.85 (d, *J* = 11.0 Hz, 1H, Ph-CH_2_), 4.84 (d, *J* = 11.0 Hz, 1H, Ph-CH_2_), 4.77 (d, *J* = 11.0 Hz, 1H, Ph-CH_2_), 4.75 (d, *J* = 11.0 Hz, 1H, Ph-CH_2_), 4.75 (d, *J* = 11.0 Hz, 1H, Ph-CH_2_), 4. 73 (d, *J* = 9.1, 2.5 Hz, 2H, Ph-CH_2_), 4.69 (d, *J* = 10.6 Hz, 1H, Ph-CH_2_), 4.68 (d, *J* = 11.2 Hz, 1H, Ph-CH_2_), 4.65 (d, *J* = 11.2 Hz, 1H, Ph-CH_2_), 4.64 (d, *J* = 11.0 Hz, 1H, Ph-CH_2_), 4.63 (d, *J* = 10.4 Hz, 1H, Ph-CH_2_), 4.62 (d, *J* = 10.7 Hz, 1H, Ph-CH_2_), 4.61 (d, *J* = 11.0 Hz, 1H, Ph-CH_2_), 4.59 (d, *J* = 10.9 Hz, 1H, Ph-CH_2_), 4.56 (d, *J* = 11.0 Hz, 1H, Ph-CH_2_), 4.45 (s, 1H, NHBoc), 4.34 (d, *J* = 8.5 Hz, 1H, 1-H), 4.28 (d, *J* = 8.6 Hz, 1H, 1-H), 4.23 (d, *J* = 9.6 Hz, 1H, 1-H), 4.21 (d, *J* = 9.2 Hz, 1H, 1-H), 3.90 – 3.69 (m, 13H), 3.62 (dt, *J* = 13.6, 5.3 Hz, 3H), 3.48 (m, 9H, OMe) , 3.38 – 3.46 (m, 5H, 7-H/3-H ), 3.37 (s, 3H, OMe), 3.29 (ddt, *J* = 21.6, 13.4, 4.7 Hz, 2H, 7-H), 3.14 (m, 1H, 7-H), 3.00 (ddd, *J* = 14.0, 5.1, 2.9 Hz, 1H, 7-H), 1.85 (tt, *J* = 12.7, 6.2 Hz, 3H, 2-H), 1.76 (t, *J* = 4.7 Hz, 1H, 2-H), 1.44 (s, 9H, Boc); ^13^C NMR (151 MHz, Chloroform-*d*) δ = 170.1, 168.9, 168.7, 168.6, 155.8, 137.8, 137.7, 137.7, 137.7, 137.6, 137.5, 137.5, 128.6, 128.6, 128.7, 128.5, 128.5, 128.5, 128.4, 128.3, 128.1, 128.0, 128.0, 127.9, 127.9, 103.2, 102.9, 100.8, 81.0, 80.7, 79.7, 79.3, 79.0, 78.5, 78.1, 75.8, 75.7, 75.5, 75.1, 74.9, 74.3, 74.3, 74.2, 74.1, 73.9, 73.8, 70.9, 57.1, 57.0, 56.9, 55.4, 52.5, 47.00, 46.3, 46.2, 45.1, 38.2, 37.8, 37.0, 28.4; IR (film): ν = 3296, 2931, 1659, 1104, 1071, 1028, 736, 697 cm^-1^; HRMS (ESI) m/z calcd for C_94_H_112_N_4_O_23_Na [M + Na]^+^ 1687.7615, found 1687.7638.

**Synthesis of compound (18)**

To a stirred solution of **17** (150 mg, 0.09 mmol) in dry DCM (0.6 mL) at 0 °C was added trifluoroacetic acid (TFA) (0.2 mL) and the mixture was stirred for 2h at room temperature. The reaction mixture was then concentrated in vacuo to obtain the trifluoroacetate salt **17a**.

To a stirring solution of **17** (150 mg, 0.09 mmol) in THF/MeOH/H_2_O (0.6/0.2/0.2 mL) at 0 °C was added lithium hydroxide (LiOH·H_2_O) (11 mg, 0.27 mmol) and the mixture was stirred at room temperature for 1 h. The reaction mixture was then acidified to pH 2 with 1 N HCl. The reaction mixture was extracted with EtOAc (2×10 mL). The combined organic extracts were washed with water, brine, dried (Na_2_SO_4_), filtered and concentrated in vacuo to obtain the crude acid **17b**, which was used for the next reaction without further purification.

To a stirring solution of the crude acid **17b** in dry DMF (0.5 mL) at 0 °C were sequentially added Et_3_N (38 μL, 0.27 mmol) and DPPA (30 μL, 0.135 mmol). After 10 min, the above prepared trifluoroacetate salt **17a** was dissolved in DMF (0.4 mL) and added to the reaction mixture. After stirring for 15 h at room temperature, the reaction mixture was diluted with CH_2_Cl_2_, washed with 1 N HCl solution, saturated NaHCO_3_ solution, water, brine, dried (Na_2_SO_4_), filtered and concentrated in vacuo. Purification by silica gel column chromatography (dichloromethane /methanol = 100:1-75:1-50:1) afforded compound **18**. Yield: (152 mg, 53%).

R_f_ = 0.31 (dichloromethane /methanol = 40:1); [α]_D_^25^ = + 21.1 (c = 1.0 in CHCl_3_); ^1^H NMR (600 MHz, Chloroform-*d*) δ = 7.84 – 7.09 (m, 80H, arom. H,), 7.01 (t, *J* = 6.5 Hz, 2H, NHCO), 6.83 (q, *J* = 4.6, 3.9 Hz, 2H, NHCO), 6.78 – 6.72 (m, 1H, NHCO) 6.35 (t, *J* = 5.5 Hz, 1H, NHCO), 6.27 (q, *J* = 5.8 Hz, 1H, NHCO), 5.01 – 4.47 (m, 32H, Ph-CH_2_), 4.44 (s, 1H, NHBoc), 4.34 – 4.15 (m, 8H, 1-H), 3.98 – 3.89 (m, 2H), 3.90 – 3.78 (m, 7H), 3.75 (s, 3H, COOMe), 3.78 – 3.65 (m, 11H), 3.59 (ddd, *J* = 14.6, 11.2, 6.0 Hz, 2H), 3.53 – 3.21 (m, 23H), 3.46 (s, 3H, OMe), 3.45 (s, 6H, OMe), 3.44 (s, 3H, OMe), 3.42 (s, 3H, OMe), 3.37 (s, 3H, OMe), 3.27 (s, 3H, OMe), 3.23 (s, 3H, OMe), 3.16 – 3.09 (m, 1H, 7-H), 3.05 (dtd, *J* = 14.2, 6.9, 2.5 Hz, 1H, 7-H), 2.98 – 2.92 (m, 1H, 7-H), 2.87 (dt, *J* = 12.7, 6.1 Hz, 1H, 7-H), 2.04 – 1.62 (m, 8H, 2-H), 1.41 (s, 9H, Boc); ^13^C NMR (151 MHz, Chloroform-*d*) δ = 170.1, 169.0, 168.9, 168.7, 168.6, 155.8, 138.0, 137.8, 137.8, 137.6, 137.5, 137.4, 128.7, 128.6, 128.6, 128.5, 128.5, 128.5, 128.4, 128.3, 128.2, 128.2, 128.1, 128.0, 127.9, 127.8, 127.8, 127.7, 127.7, 103.2, 103.0, 102.9, 100.8, 100.7, 100.5, 100.4, 81.9, 81.6, 81.2, 81.0, 80.6, 80.2, 80.0, 79.7, 79.3, 79.1, 78.7, 78.2, 75.9, 75.8, 75.7, 75.6, 75.2, 75.0, 74.9, 74.6, 74.2, 74.2, 74.1, 74.0, 73.9, 73.8, 71.6, 71.6, 70.9, 57.1, 57.0, 56.9, 55.5, 55.5, 55.3, 52.5, 47.0, 46.3, 46.2, 45.2, 45.2, 45.0, 38.2, 37.8, 37.4, 29.7, 28.4; IR (film): ν = 3306, 2925, 1660, 1557, 1453, 1363, 1269, 1067, 1027, 735, 696 cm^-1^; MALDI-TOF-MS m/z calcd for C_182_H_212_N_8_O_43_Na [M + Na]^+^ 3220.455, found 3220.983.

**Compound 19, 20, 21, 22, 23 and 24 were synthesized following same procedure as described for the synthesis of 16, 17 and 18.**

**Compound (19)**

Yield: (341 mg, 77%); R_f_ = 0.45 (dichloromethane /methanol = 60:1); [α]_D_^25^ = + 37.9 (c = 1.0 in CHCl_3_); ^1^H NMR (600 MHz, Chloroform-*d*) δ = 7.18 – 7.43 (m, 20H, arom. H), 7.13 (t, *J* = 6.10 Hz, 1H, NHCO), 4.94 (d, *J* = 11.22 Hz, 1H, Ph-CH_2_), 4.77 (d, *J* = 11.68 Hz, 1H, Ph-CH_2_), 4.74 (d, *J* = 11.69 Hz, 1H, Ph-CH_2_), 4.66 (d, *J* = 11.22 Hz, 1H, Ph-CH_2_), 4.64 (d, *J* = 11.30 Hz, 1H, Ph-CH_2_), 4.56 (d, *J* = 11.62 Hz, 1H, Ph-CH_2_), 4.55 (s, 1H, NHBoc), 4.49 – 4.53 (m, 1H, 4-H, Res-II), 4.46 (d, *J* = 11.42 Hz, 1H, Ph-CH_2_), 4.44 (d, *J* = 12.16 Hz, 1H, Ph-CH_2_), 4.15 (d, *J* = 8.71 Hz, 2H, 1-H(Res-II), 4-H(Res-I)), 4.11 (d, *J* = 8.74 Hz, 1H, 1-H, Res-I), 3.88 (s, 1H, 5-H, Res-II), 3.71 (s, 3H, COOMe), 3.65 (ddd, *J* = 13.92, 6.14, 3.98 Hz, 1H, 7-H, Res-I), 3.59 (s, 3H, OMe), 3.52 – 3.57 (m, 2H, 7-H, Res-I/Res-II), 3.52 (s, 1H, 5-H, Res-I), 3.43 (s, 3H, OMe), 3.38 (dd, *J* = 11.18, 2.37 Hz, 1H, Res-II), 3.33 (dd, *J* = 11.39, 2.48 Hz, 1H, Res-I), 3.19 (s, 1H, 7-H, Res-II), 2.30 – 2.39 (m, 1H, 2-H, Res-I ), 2.23 (tt, *J* = 9.32, 4.53 Hz, 1H, 2-H, Res-II), 1.44 (s, 9H, Boc); ^13^C NMR (151 MHz, Chloroform-*d*) δ = 168.9, 168.6, 155.9, 138.5, 138.1, 137.1, 137.1, 128.8, 128.6, 128.4, 128.3, 128.2, 128.1, 128.0, 127.9, 127.8, 127.6, 103.5, 103.2, 78.9, 77.5, 74.7, 74.4, 74.1, 73.8, 71.8, 71.3, 71.3, 70.7, 57.2, 56.9, 52.3, 42.1, 41.7, 37.8, 36.4, 28.4; IR (film): ν = 3422, 2923, 2851, 1713, 1677, 1529, 1497, 1454, 1364, 1163, 1073, 1028, 735, 697 cm^-1^; HRMS (ESI) m/z calcd for C_50_H_62_N_2_O_13_Na [M + Na]^+^ 921.4150, found 921.4154.

**Compound (20)**

Yield: (180 mg, 60%); R_f_ = 0.39 (dichloromethane /methanol = 40:1); [α]_D_^25^ = + 42.1 (c = 1.0 in CHCl_3_); ^1^H NMR (600 MHz, Chloroform-*d*) δ = 7.41 – 7.17 (m, 40H, arom. H), 7.15 (m, 1H, NHCO), 7.12 (t, *J* = 7.4, 7.4 Hz, 1H, NHCO), 7.05 (t, *J* = 6.1, 6.1 Hz, 1H, NHCO), 4.86 (d, *J* = 11.2 Hz, 1H, Ph-CH_2_), 4.84 (d, *J* = 11.3 Hz, 1H, Ph-CH_2_), 4.81 (d, *J* = 11.2 Hz, 2H, Ph-CH_2_), 4.79 (d, *J* = 11.1 Hz, 1H, Ph-CH_2_), 4.78 (d, *J* = 11.2 Hz, 1H, Ph-CH_2_), 4.74 (d, *J* = 11.6 Hz, 1H, Ph-CH_2_), 4.72 (d, *J* = 11.3 Hz, 1H, Ph-CH_2_), 4.70 (d, *J* = 11.4 Hz, 1H, Ph-CH_2_), 4.69 (d, *J* = 11.7 Hz, 1H, Ph-CH_2_), 4.62 (d, *J* = 11.4 Hz, 4H, Ph-CH_2_), 4.58 (d, *J* = 11.3 Hz, 1H, Ph-CH_2_), 4.55 (d, *J* = 11.2 Hz, 1H, Ph-CH_2_), 4.53 (s, 1H, NHBoc), 4.40 (d, *J* = 11.3 Hz, 1H, Ph-CH_2_), 4.49 – 4.40 (m, 2H, 4-H), 4.38 (d, *J* = 11.3 Hz, 1H, Ph-CH_2_), 4.39 – 4.36 (m, 1H, 4-H), 4.36 (d, *J* = 11.3 Hz, 1H, Ph-CH_2_), 4.34 (d, *J* = 11.3 Hz, 1H, Ph-CH_2_), 4.11 (d, *J* = 9.1 Hz, 1H, 1-H), 4.12 – 4.09 (m, 1H, 4-H), 4.09 (d, *J* = 9.2 Hz, 1H, 1-H), 4.07 (d, *J* = 9.0 Hz, 1H, 1-H), 4.06 (d, *J* = 9.1 Hz, 1H, 1-H), 3.93 (ddd, *J* = 14.0, 9.2, 4.7 Hz, 1H, 7-H), 3.85 – 3.83 (m, 2H, 5-H), 3.82 (dd, *J* = 11.4, 2.6 Hz, 1H), 3.70 (s, 3H, COOMe), 3.69 – 3.67 (m, 1H, 5-H), 3.64 – 3.57 (m, 4H), 3.56 (s, 3H, OMe), 3.55 – 3.42 (m, 6H), 3.39 (s, 3H, OMe), 3.34 (s, 3H, OMe), 3.33 (s, 3H, OMe), 3.32 – 3.26 (m, 3H), 3.21 – 3.10 (m, 2H, 3-H ), 3.00 – 2.84 (m, 1H, 7-H), 2.57 – 2.47 (m, 1H, 7-H), 2.35 – 2.23 (m, 2H, 2-H), 2.23 – 2.14 (m, 2H, 2-H), 1.41 (s, 9H, Boc); ^13^C NMR (151 MHz, Chloroform-*d*) δ = 169.6, 168.9, 168.5, 168.3, 168.1, 155.9, 138.7, 138.4, 138.2, 138.1, 137.1, 137.0, 136.9, 136.7, 128.7, 128.7, 128.6, 128.6, 128.4, 128.3, 128.2, 128.2, 128.1, 128.1, 128.0, 128.0, 127.9, 127.9, 127.8, 127.8, 127.8, 127.7, 127.7, 127.7, 127.6, 127.6, 127.6, 127.5, 127.5, 127.4, 127.4, 103.4, 103.2, 103.1, 101.1, 79.3, 79.1, 78.9, 78.9, 78.9, 77.5, 74.8, 74.7, 74.6, 74.6, 74.4, 74.4, 74.0, 73.7, 73.3, 72.0, 71.8, 71.7, 71.7, 71.6, 71.4, 71.4, 71.3, 71.1, 71.1, 70.9, 70.7, 57.2, 56.7, 56.6, 56.3, 56.0, 52.4, 52.3, 42.1, 41.7, 41.6, 41.5, 39.7, 37.7, 37.2, 37.0, 36.5, 28.4; IR (film): ν = 3419, 2930, 2866, 1672, 1529, 1454, 1364, 1154, 1071, 1027, 736, 697 cm^-1^; HRMS (ESI) m/z calcd for C_94_H_112_N_4_O_23_Na [M + Na]^+^ 1687.7615, found 1687.7604.

**Compound (21)**

Yield: (40 mg, 52%); R_f_ = 0.23 (dichloromethane /methanol = 40:1); [α]_D_^25^ = + 50.5 (c = 1.0 in CHCl_3_); ^1^H NMR (400 MHz, Chloroform-*d*) δ = 7.64 – 7.07 (m, 86H, arom. H / NHCO), 7.08 – 6.94 (m, 1H, NHCO), 4.90 – 4.50 (m, 26H, Ph-CH_2_ / NHBoc), 4.47 – 4.27 (m, 18H, Ph-CH_2_ / 4-H / 5-H), 4.19 – 4.01 (m, 9H, 1-H, 4-H), 3.93 (ddd, *J* = 13.9, 9.1, 4.8 Hz, 1H, 7-H), 3.82 (s, 2H, 5-H), 3.81 – 3.78 (m, 1H, 7-H), 3.78 – 3.71 (m, 1H, 7-H), 3.69 (s, 3H, COOMe), 3.63 – 3.44 (m, 14H), 3.43 – 3.13 (m, 40H), 3.40 (s, 3H, OMe), 3.39 (s, 3H, OMe), 3.38 (s, 3H, OMe), 3.36 (s, 3H, OMe), 3.35 (s, 3H, OMe), 3.34 (s, 3H, OMe), 3.33 (s, 3H, OMe), 3.30 (s, 3H, OMe), 2.98 – 2.81 (m, 2H, 7-H), 2.51 (dq, *J* = 10.5, 5.2 Hz, 1H, 7-H), 2.43 – 2.08 (m, 8H, 2-H), 1.41 (s, 9H, Boc); ^13^C NMR (101 MHz, Chloroform-*d*) δ = 169.6, 169.1, 168.4, 168.2, 168.2, 168.1, 168.1, 168.0, 155.8, 138.7, 138.5, 138.4, 138.2, 137.2, 137.1, 137.0, 136.8, 128.7, 128.7, 128.6, 128.6, 128.5, 128.4, 128.2, 128.2, 128.2, 128.1, 128.0, 128.0, 128.0, 127.9, 127.8, 127.8, 127.7, 127.6, 127.6 127.6, 127.4, 127.4, 103.5, 103.4, 103.3, 103.3, 103.2, 101.1, 100.9, 79.2, 79.2, 79.0, 78.8, 77.7, 74.8, 74.7, 74.6, 74.4, 73.4, 73.1, 72.1, 71.9, 71.8, 71.7, 71.6, 71.5, 71.5, 71.2, 71.0, 70.8, 70.7, 56.8, 56.7, 56.6, 56.5, 56.3, 56.2, 55.9, 55.4, 52.3, 42.2, 41.7, 41.6, 41.4, 39.8, 39.8, 37.8, 37.7, 37.2, 37.0, 29.7, 28.4; IR (film): ν = 3413, 2931, 1672, 1529, 1454, 1149, 1071, 1027, 735, 697 cm^-1^; MALDI-TOF-MS m/z calcd for C_182_H_212_N_8_O_43_Na [M + Na]^+^ 3220.455, found 3220.947.

**Compound (22)**

Yield: (600 mg, 50%); R_f_ = 0.32 (petroleum ether /ethyl acetate = 1:1); [α]_D_^25^ = + 20.9 (c = 1.0 in CHCl_3_); ^1^H NMR (600 MHz, Chloroform-*d*) δ = 7.48 – 7.16 (m, 20H, arom. H), 7.10 (t, *J* = 5.8 Hz, 1H, NHCO), 4.82 (d, *J* = 10.9 Hz, 1H, Ph-CH_2_), 4.78 (d, *J* = 11.3 Hz, 1H, Ph-CH_2_), 4.72(d, *J* = 10.8 Hz, 1H, Ph-CH_2_), 4.70 (d, *J* = 11.0 Hz, 1H, Ph-CH_2_), 4.65 (d, *J* = 11.7 Hz, 1H, Ph-CH_2_), 4.61 (d, *J* = 11.3 Hz, 1H, Ph-CH_2_), 4.57 (d, *J* = 10.8 Hz, 1H, Ph-CH_2_), 4.54 (t, *J* = 6.0 Hz, 1H, NHBoc), 4.42 (dd, *J* = 2.8, 1.3 Hz, 1H, 4-H, Res-II), 4.35 (d, *J* = 11.7 Hz, 1H, Ph-CH_2_), 4.21 (d, *J* = 8.5 Hz, 1H, Res-I), 4.11 (d, *J* = 8.7 Hz, 1H, Res-II), 3.82 (dd, *J* = 4.0, 1.0 Hz, 3H, 4-H,5-H(Res-I)/5-H(Res-II)), 3.74 (s, 3H, COOMe), 3.57 – 3.48 (m, 3H, 7-H,7’-H(Res-I)/7-H(Res-II)), 3.47 (s, 3H, OMe), 3.46 (s, 3H, OMe), 3.41 (dt, *J* = 10.8, 4.3 Hz, 1H, 3-H, Res-I), 3.31 (dd, *J* = 11.3, 2.6 Hz, 1H, 3-H, Res-II), 3.18 - 3.12 (m, 1H, 7-H, Res-II), 2.15 (ddt, *J* = 13.6, 9.1, 4.6 Hz, 1H, 2-H, Res-II), 1.87 (dddd, *J* = 10.5, 8.5, 5.8, 4.5 Hz, 1H, 2-H, Res-I), 1.42 (s, 9H, Boc); ^13^C NMR (151 MHz, Chloroform-*d*) δ = 169.2, 168.6, 155.9, 138.3, 137.9, 137.6, 137.1, 128.7, 128.4, 128.4, 128.2, 128.2, 127.9, 127.9, 127.7, 127.5, 103.8, 103.5, 80.9, 79.8, 77.5, 74.8, 74.7, 74.6, 74.5, 71.5, 70.7, 57.0, 56.8, 52.5, 46.6, 42.0, 37.8, 36.7, 28.4; IR (film): ν = 3429, 2931, 1744, 1712, 1654, 1535, 1497, 1453, 1365, 1244, 1157, 1086, 1069, 1024, 733, 696 cm^-1^; HRMS (ESI) m/z calcd for C_50_H_62_N_2_O_13_Na [M + Na]^+^ 921.4150, found 921.4146.

**Compound (23)**

Yield: (281 mg, 65%); R_f_ = 0.39 (dichloromethane /methanol = 50:1); [α]_D_^25^ = + 28.0 (c = 1.0 in CHCl_3_); ^1^H NMR (600 MHz, Chloroform-*d*) δ = 7.60 – 7.16 (m, 40H, arom. H), 7.14 (t, *J* = 5.5 Hz, 1H, NHCO), 6.93 (t, *J* = 5.5 Hz, 1H, NHCO), 4.80 (d, *J* = 11.2 Hz, 1H Ph-CH_2_) 4.78 (d, *J* = 11.2 Hz, 1H, Ph-CH_2_), 4.76 (d, *J* = 11.4 Hz, 1H, Ph-CH_2_), 4.75 (d, *J* = 10.7 Hz, 2H, Ph-CH_2_), 4.73 (d, *J* = 10.8 Hz, 1H, Ph-CH_2_), 4.72 (d, *J* = 10.8 Hz, 1H, Ph-CH_2_), 4.69 (d, *J* = 11.1 Hz, 1H, Ph-CH_2_), 4.67 (d, *J* = 11.9 Hz, 1H, Ph-CH_2_), 4.65 (d, *J* = 11.6 Hz, 1H, Ph-CH_2_), 4.64 (d, *J* = 10.7 Hz, 4H, Ph-CH_2_), 4.63 (d, *J* = 11.0 Hz, 1H, Ph-CH_2_), 4.61 (d, *J* = 11.5 Hz, 1H, Ph-CH_2_), 4.58 (d, *J* = 10.9 Hz, 1H, Ph-CH_2_), 4.56 (d, *J* = 11.4 Hz, 1H, Ph-CH_2_), 4.54 (t, *J* = 6.02 Hz, 1H, NHBoc), 4.53 (d, *J* = 10.6 Hz, 1H, Ph-CH_2_), 4.45 – 4.38 (m, 3H, 4-H), 4.41 (d, *J* = 11.4 Hz, 1H, Ph-CH_2_), 4.21 (d, *J* = 9.2 Hz, 1H, 1-H), 4.20 (d, *J* = 9.1 Hz, 1H, 1-H), 4.15 (d, *J* = 8.6 Hz, 1H, 1-H), 4.11 (d, *J* = 8.7 Hz, 1H, 1-H), 4.00 (ddd, *J* = 14.4, 9.0, 5.6 Hz, 1H, 7-H), 3.83 – 3.76 (m, 4H, 4-H/5-H), 3.73 (s, 3H, COOMe), 3.73 – 3.68 (m, 1H,), 3.64 – 3.57 (m, 1H, 7-H), 3.60 (t, 1H, 3-H), 3.54 – 3.47 (m, 1H, 7-H), 3.56 – 3.27 (m, 8H), 3.47 (s, 3H, OMe), 3.45 (s, 3H, OMe), 3.40 (s, 3H, OMe), 3.34 (s, 3H, OMe), 3.21 – 3.09 (m, 1H, 7-H), 2.95 (dt, *J* = 14.2, 3.1 Hz, 1H, 7-H), 2.24 (td, *J* = 10.4, 9.7, 5.7 Hz, 1H), 2.15 (ddt, *J* = 15.0, 10.3, 5.3 Hz, 1H), 2.03 (h, *J* = 4.0 Hz, 1H), 1.77 (tt, *J* = 9.7, 5.5 Hz, 1H), 1.41 (s, 9H, Boc); ^13^C NMR (151 MHz, Chloroform-*d*) δ = 170.1, 168.6, 168.5, 168.3, 155.9, 138.5, 138.4, 137.9, 137.7, 137.7, 137.6, 137.2, 137.1, 128.7, 128.7, 128.5, 128.4, 128.3, 128.2, 128.2, 128.2, 128.1, 128.0, 128.0, 127.9, 127.9, 127.8, 127.7, 127.5, 127.5, 103.7, 103.5, 103.1, 101.1, 81.2, 81.0, 79.4, 79.0, 78.8, 77.5, 75.6, 75.2, 75.0, 74.9, 74.8, 74.6, 74.5, 74.3, 72.1, 71.6, 71.2, 70.9, 70.7, 56.8, 56.7, 56.7, 55.5, 52.5, 46.4, 45.1, 42.1, 41.7, 37.8, 37.5, 37.3, 36.4, 29.7; IR (film): ν = 3420, 2933, 1675, 1526, 1453, 1364, 1246, 1154, 1070, 1027, 735, 697 cm^-1^; HRMS (ESI) m/z calcd for C_94_H_112_N_4_O_23_Na [M + Na]^+^ 1687.7615, found 1687.7643.

**Compound (24)**

Yield: (75 mg, 51%); R_f_ = 0.30 (dichloromethane /methanol = 40:1); [α]_D_^25^ = + 27.1 (c = 1.0 in CHCl_3_); ^1^H NMR (600 MHz, Chloroform-*d*) δ = 7.45 – 7.16 (m, 81H, arom.H/NHCO), 7.12 (dt, *J* = 11.3, 5.6 Hz, 3H, NHCO), 6.91 (t, *J* = 5.6 Hz, 2H, NHCO), 6.30 (q, *J* = 5.7, 5.2 Hz, 1H), 4.89 – 4.47 (m, 30H, Ph-CH_2_), 4.48 – 4.30 (m, 10H, NHBoc/4-H/Ph-CH_2_), 4.25 – 4.06 (m, 9H, 1-H), 3.98 (dddd, *J* = 19.6, 14.0, 9.2, 5.6 Hz, 2H), 3.88 – 3.75 (m, 10H), 3.73 (s, 3H, COOMe), 3.73 – 3.66 (m, 5H), 3.65 – 3.52 (m, 3H), 3.53 – 3.21 (m, 45H), 3.46 (s, 3H, OMe), 3.44 (s, 6H, OMe), 3.42 (s, 3H, OMe),), 3.39 (s, 3H, OMe), 3.38 (s, 3H, OMe), 3.35 (s, 3H, OMe), 3.24 (s, 3H, OMe), 3.19 – 3.10 (m, 2H, 7-H), 2.95 (dt, *J* = 14.1, 3.2 Hz, 1H, 7-H), 2.91 – 2.83 (m, 2H, 7-H), 2.23 (dp, *J* = 11.3, 5.8 Hz, 3H, 2-H), 2.15 (tt, *J* = 9.3, 4.6 Hz, 1H, 2-H), 2.06 – 2.01 (m, 1H, 2-H), 1.86 – 1.80 (m, 1H, 2-H), 1.77 (dp, *J* = 9.9, 5.2 Hz, 2H, 2-H), 1.41 (s, 9H, Boc); ^13^C NMR (151 MHz, Chloroform-*d*) δ 170.1, 169.0, 169.0, 168.5,168.5, 168.3, 168.3, 155.9, 138.6, 138.6, 138.4, 138.2, 137.9, 137.9, 137.8, 137.8, 137.7, 137.7, 137.6, 137.6, 137.3, 137.2, 137.1, 137.1, 128.8, 128.7, 128.7, 128.5, 128.5, 128.4, 128.3, 128.3, 128.3, 128.2, 128.2, 128.1, 128.1, 128.0, 128.0, 128.0, 127.9, 127.8, 127.7, 127.6, 127.5, 127.5, 103.7, 103.7, 103.6, 103.6, 103.2, 103.1, 101.1, 100.8, 82.0, 81.2, 81.0, 79.5, 79.1, 79.1, 77.5, 77.5, 77.5, 75.6, 75.6, 75.2, 75.0, 74.9, 74.8, 74.8, 74.8, 74.6, 74.5, 74.3, 72.1, 72.1, 71.6, 71.4, 71.2, 71.2, 70.9, 70.7, 70.6, 56.7, 56.7, 56.7, 56.7, 55.4, 55.4, 52.5, 46.4, 45.1, 42.1, 41.9, 41.7, 37.6, 37.3, 36.7, 36.4, 29.7, 28.4; IR (film): ν = 3413, 2932, 1677, 1527, 1453, 1363, 1272, 1206, 1152, 1070, 1027, 735, 697 cm^-1^; MALDI-TOF-MS m/z calcd for C_182_H_212_N_8_O_43_Na [M + Na]^+^ 3220.455, found 3220.929.

**3. Conformational analysis of oligo-SAAs by NMR spectroscopy**

Solution NMR spectra were recorded on 700 MHz spectrometers at 263 K and 298K with 2-10 mM solutions in CDCl3 solvents using TMS as internal standard or the solvent signals as secondary standards and the chemical shifts (*δ*) are shown in scales. Multiplicities of NMR signals are designated as s (singlet), d (doublet), t (triplet), q (quartet), br (broad), td (triplet of a doublet), dt (doublet of a triplet) and m (multiplet, for unresolved lines). The proton resonance assignments were carried out by using ^1^H-^1^H Two dimensional total correlation spectroscopy (TOCSY), and Rotating frame nuclear Overhauser effect spectroscopy (ROESY). All the experiments were carried out in the phase sensitive mode. The spectra were acquired with 2048 × 256 or 4096 × 512 free induction decays (FID) containing 8-16 transients with relaxation delay 2 s. The TOCSY experiments were performed with mixing time of 0.08s, and spin-locking field of 10 kHz. The two dimensional data were processed with Gaussian apodization in both the dimensions. The ROESY experiments were performed with mixing times of 0.2 to 0.3s, and a spin locking field of about 2.0 kHz. Solvent titration studies were carried out by sequentially adding up to 33% (v/v) of DMSO-*d*6 (up to 300 *μ*L) to 600 *μ*L of CDCl_3_ solutions of the carbopeptoids. Small changes in amide proton chemical shifts (Δ*δ*) during solvent titrations have been used to indicate their participation in H-bonding.

**Table 1:** Chemical shift (δ in ppm) and coupling constants (*J* in Hz) for **17** in CDCl3 (700 MHz, 263K).

| Protons | Res-1 | Res-2 | Res-3 | Res-4 |
| --- | --- | --- | --- | --- |
| NH | 4.27 (1H, t, *J*_NH-H7(pro-S),H7(_*_pro-R_*) = 6.1 Hz) | 4.27 (1H, m) | 6.90 (1H, t, *J*_NH-H7(pro-S),H7(_*_pro-R_*) = 4.5 Hz) | 7.14 (1H, d, *J*_NH-H7(pro-S),H7(_*_pro-R_*) = 8.8 Hz) |
| H7(*_pro-S_*) | 3.08 (1H, dd, *J* = 13.2, 6.3 Hz) | 3.28 (1H, m) | 3.23 (1H, ddd, *J* = 12.4, 7.2, 3.4 Hz) | 2.93 (1H, dd, *J* = 14.2, 4.5 Hz) |
| H7(*_pro-R_*) | 3.42 (1H, m) | 3.64 (1H, m) | 3.67 (1H, m) | 3.92 (1H, m) |
| H2 | 1.70 (1H, tt, *J* = 8.9, 4.1 Hz) | 1.98 (1H, m) | 1.82 (1H, m) | 1.82 (1H, m) |
| H1 | 4.13 (1H, dd, *J* = 8.9, 2.3 Hz) | 4.22 (1H, d, *J* = 8.1 Hz) | 4.30 (1H, dd, *J* = 8.7, 2.3 Hz) | 4.71 (1H, m) |
| H3 | 3.40 (1H, m) | 3.38 (1H, m) | 3.38 (1H, m) | 3.82 (1H, m) |
| H4 | 3.64 (1H, m) | 3.64 (1H, m) | 3.65 (1H, m) | 3.74 (1H, m) |
| H5 | 3.70 (1H, m) | 3.70 (1H, m) | 3.84 (1H, m) | 4.23 (1H, m) |

**Others:** Aromatic proton = 7.47 – 7.23 (40H, m), Phenyl CH_2_ = 4.90 – 4.83 (3H, m), 4.79 – 4.73 (3H, m), 4.69 (1H, d, *J* = 10.8 Hz), 4.67 – 4.60 (m, 6H), 4.60 – 4.53 (m, 3H), COOCH_3_ = 3.76 (3H, s), OCH_3_ = 3.48 (9H, s), 3.35 (3H, s), Boc =1.41 (9H, s).


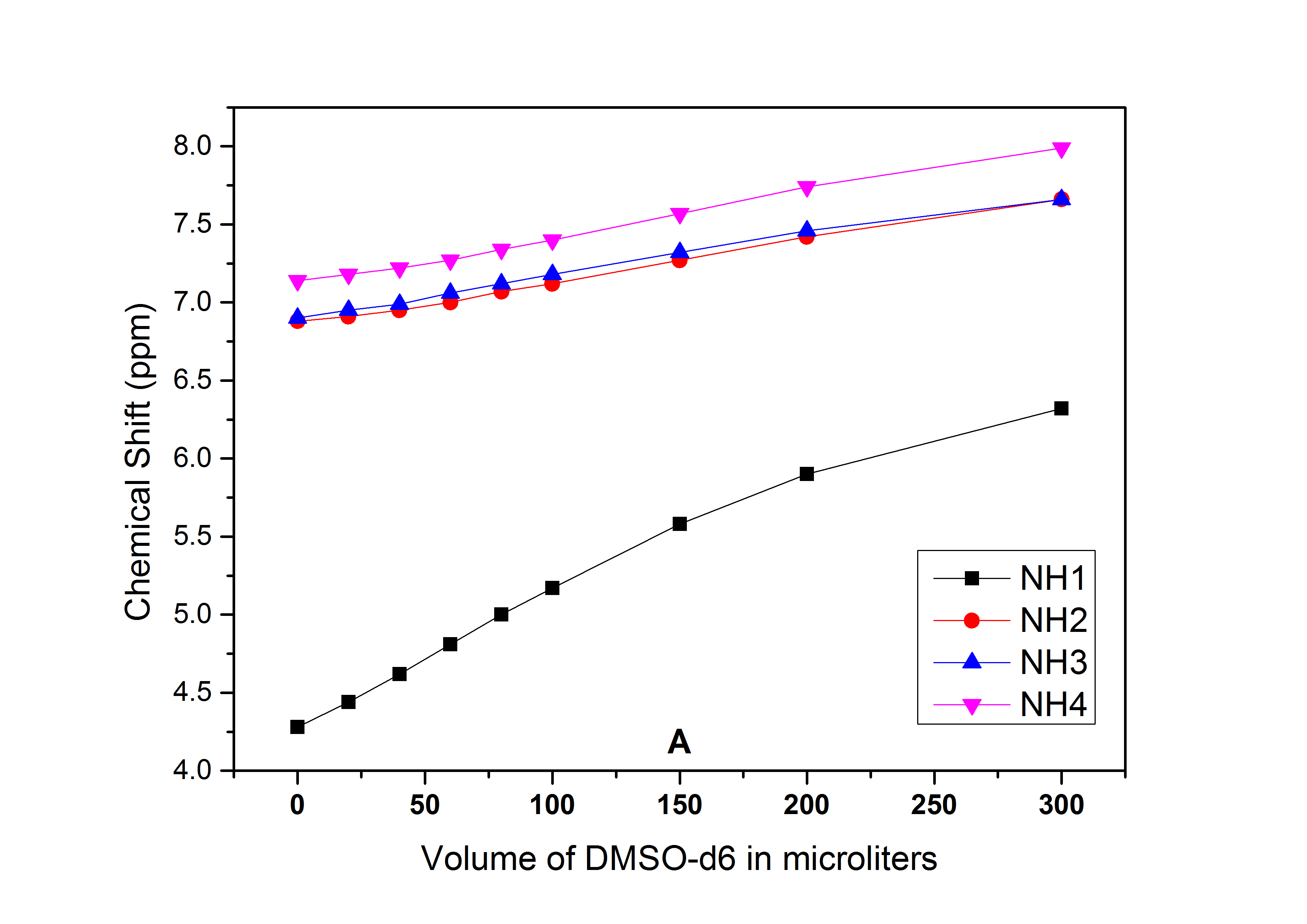


**Figure 1:** Solvent Titration plot of **17**

**^1^H NMR spectrum of 17 (700 MHz, CDCl_3_, 263K)**


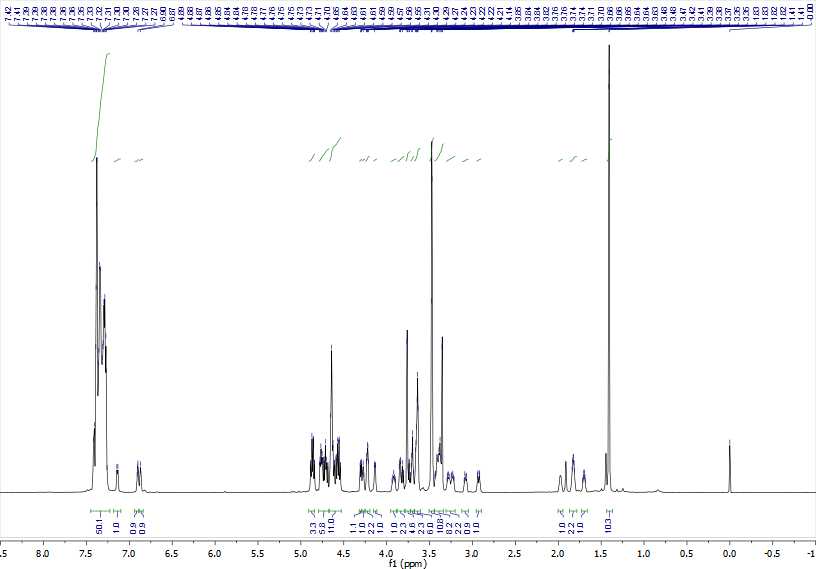


**TOCSY spectrum of 17 (700 MHz, CDCl_3_, 263K)**


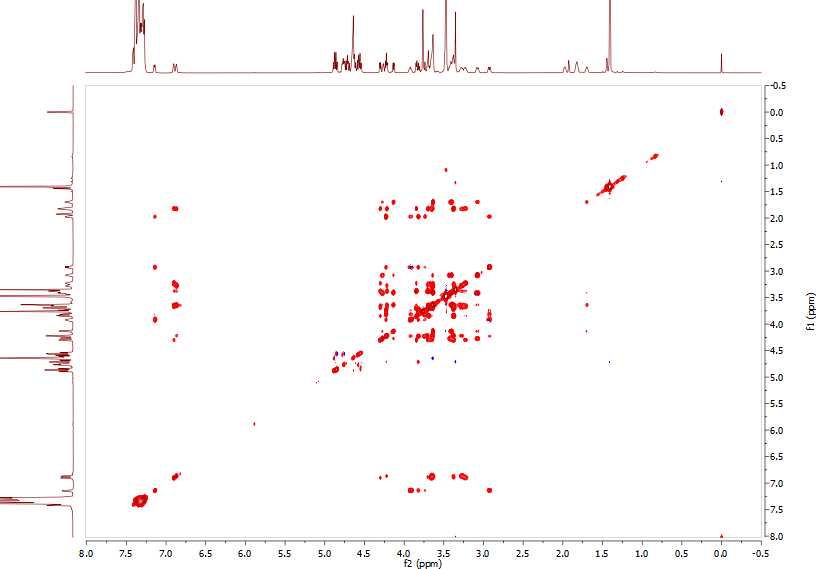


**ROESY spectrum of 17 (700 MHz, CDCl_3_, 263K)**


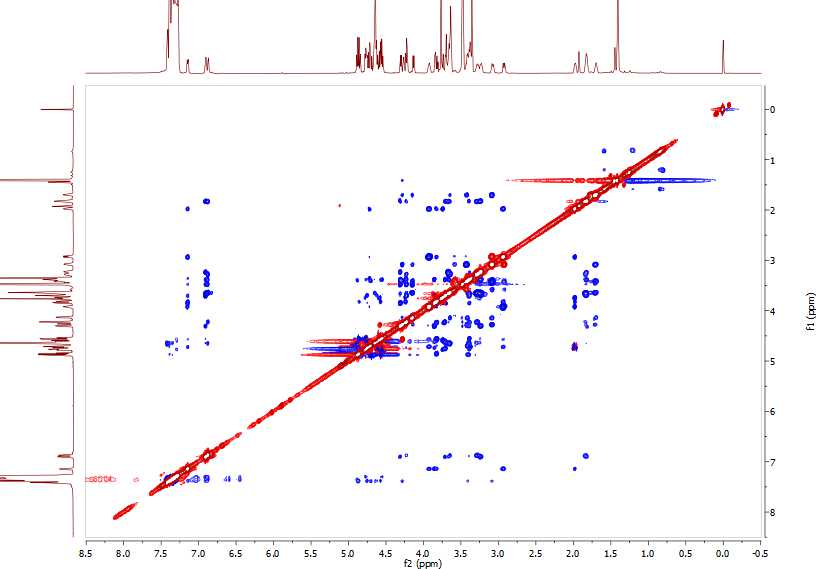


**Table 2:** Chemical shift (δ in ppm) and coupling constants (*J* in Hz) for **20** in CDCl3 (700 MHz, 298K).

| Protons | Res-1 | Res-2 | Res-3 | Res-4 |
| --- | --- | --- | --- | --- |
| NH | 4.54 (1H, m) | 7.25 (1H, m) | 7.21 (1H, m) | 7.04 (1H, d, *J*_NH-H7(pro-S),H7(_*_pro-R_*) = 6.1 Hz) |
| H7(*_pro-S_*) | 3.17 (1H, m) | 3.49 (1H, m) | 3.49 (1H, m) | 3.50 (1H, m) |
| H7(*_pro-R_*) | 3.49 (1H, m) | 3.55 (1H, m) | 3.55 (1H, m) | 3.62 (1H, m) |
| H2 | 2.18 (1H, m) | 2.27 (1H, m) | 2.27 (1H, m) | 2.30 (1H, m) |
| H1 | 4.11 (1H, m) | 4.10 (1H, m) | 4.10 (1H, m) | 4.06 (1H, dd, *J* = 8.8, 2.1 Hz) |
| H3 | 3.34 (1H, m) | 3.31 (1H, m) | 3.31 (1H, m) | 3.29 (1H, m) |
| H4 | 4.45 (1H, s) | 4.36 (1H, s) | 4.34 (1H, m) | 4.11 (1H, s) |
| H5 | 3.82 (1H, s) | 3.55 (1H, s) | 3.56 (1H, s) | 3.47 (1H, s) |

**Others:** Aromatic proton = 7.42 – 7.13 (40H, m), Phenyl CH_2_ = 4.85 (1H, d, *J* = 11.3 Hz), 4.83 (1H, d, *J* = 11.3 Hz), 4.79 (1H, d, *J* = 11.1 Hz), 4.73 (1H, d, *J* = 11.7), 4.69 (1H, d, *J* = 11.8 Hz), 4.65 – 4.60 (3H, m), 4.60 – 4.56 (2H, m), 4.53 (1H, d, *J* = 11.1 Hz), 4.43 – 4.37 (3H, m), COOCH_3_ = 3.68 (3H, s), OCH_3_ = 3.56 (3H, s), 3.39 (3H, s), 3.36(3H, s), 3.34 (3H, s), Boc =1.41 (9H, s).


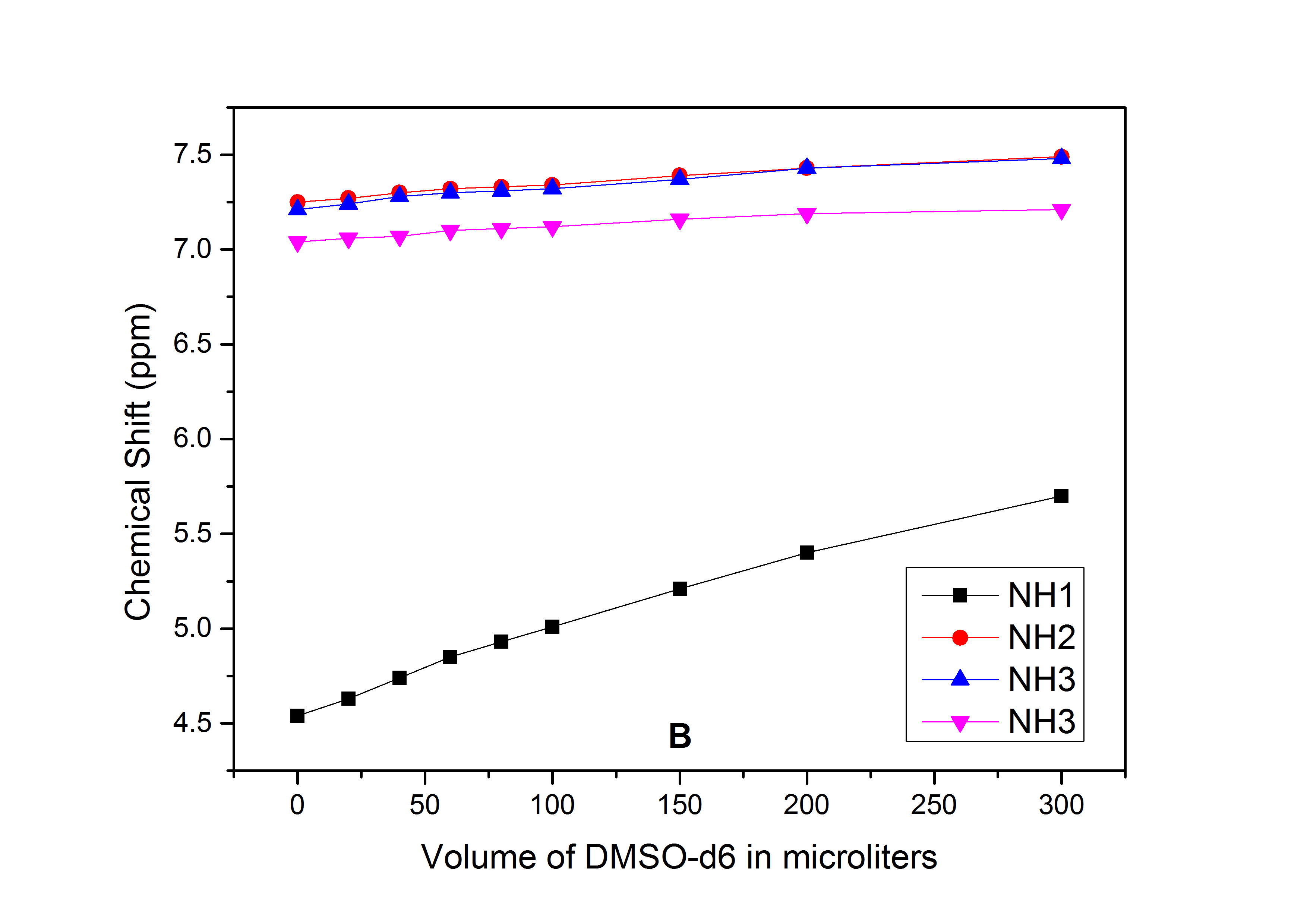


**Figure 2:** Solvent Titration plot of **20**

**^1^H NMR spectrum of 20 (700 MHz, CDCl_3_, 298K)**


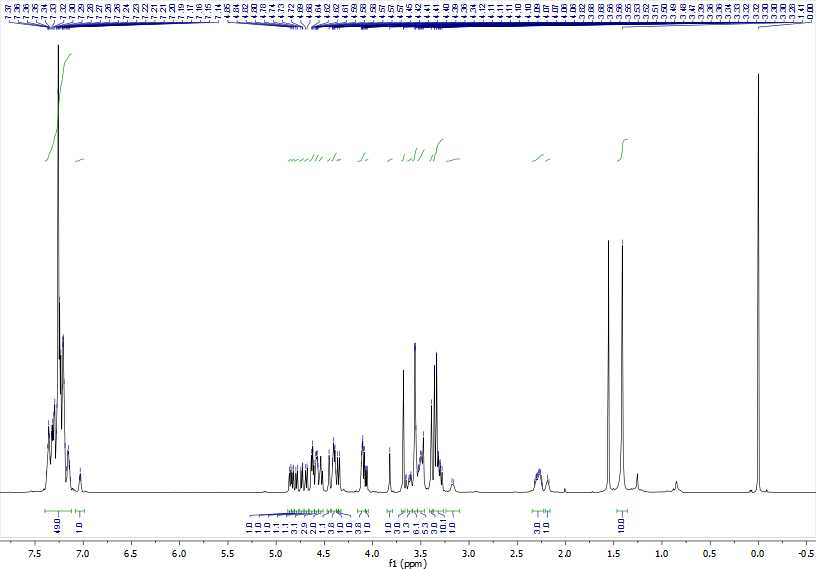


**TOCSY spectrum of 20 (700 MHz, CDCl_3_, 298K)**


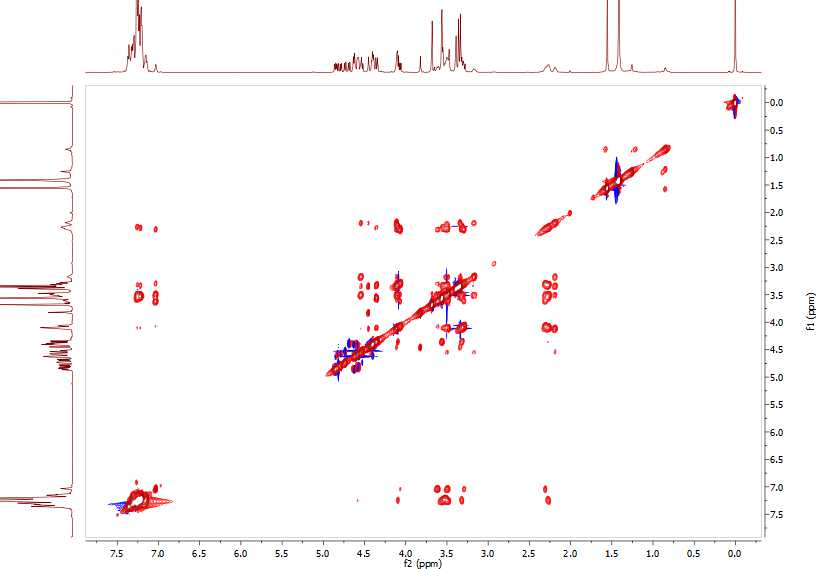


**ROESY spectrum of 20 (700 MHz, CDCl_3_, 298K)**


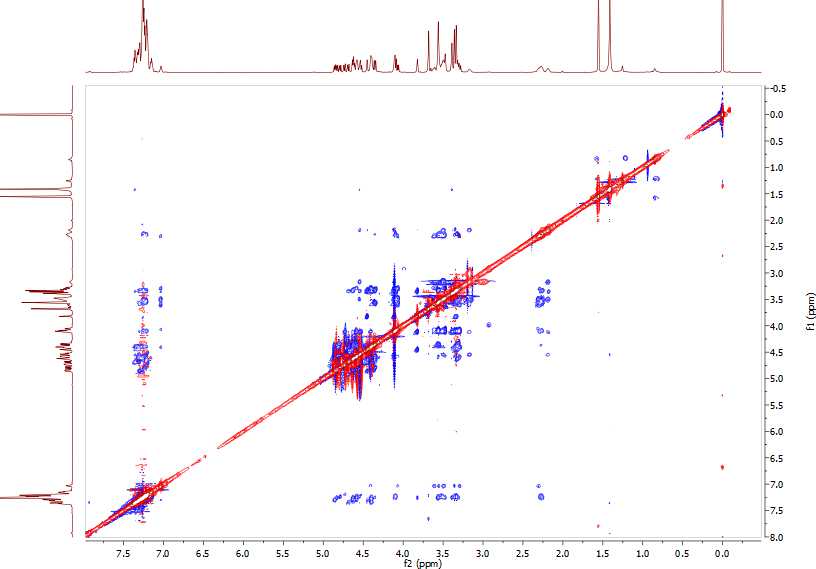


**Table 3:** Chemical shift (δ in ppm) and coupling constants (*J* in Hz) for **23** in CDCl3 (700 MHz, 298K).

| Protons | Res-1 | Res-2 | Res-3 | Res-4 |
| --- | --- | --- | --- | --- |
| NH | 4.52 (1H, m) | 7.13 (1H, t, *J* = 5.9 Hz) | 6.92 (1H, t, *J* = 5.7 Hz) | 7.32 (1H, m) |
| H7(*_pro-S_*) | 3.15 (1H, dd, *J* = 13.1, 6.6 Hz) | 3.45 (1H, m) | 3.38 (1H, m) | 2.95 (1H, dd, *J* = 14.1, 3.5 Hz) |
| H7(*_pro-R_*) | 3.49 (1H, m) | 3.49 (1H, m) | 3.59 (1H, m) | 4.00 (1H, dt, *J* = 14.3, 7.3 Hz) |
| H2 | 2.15 (1H, tt, *J* = 10.7, 4.7 Hz) | 1.78 (1H, td, *J* = 9.5, 9.0, 4.3 Hz) | 2.23 (1H, m) | 2.03 (1H, dt, *J* = 10.0, 4.8 Hz) |
| H1 | 4.11 (1H, dd, *J* = 8.8, 2.0 Hz) | 4.20 (1H, d, *J* = 8.4 Hz) | 4.15 (1H, dd, *J* = 8.7, 2.1 Hz) | 4.71 (1H, m) |
| H3 | 3.31 (1H, m) | 3.37 (1H, m) | 3.34 (1H, m) | 3.77 (1H, m) |
| H4 | 4.42 (1H, m) | 3.60 (1H, m) | 4.40 (1H, m) | 3.20 (1H, m) |
| H5 | 3.80 (1H, m) | 3.71 (1H, dd, *J* = 8.4, 2.0 Hz) | 3.78 (1H, m) | 3.78 (1H, m) |

**Others:** Aromatic proton = 7.39 – 7.16 (40H, m), Phenyl CH_2_ = 4.81 – 4.73 (4H, m), 4.69 – 4.56 (9H, m), 4.52 (1H, m), 4.41 (1H, m), 4.35 (1H, d, *J* = 11.7 Hz), COOCH_3_ = 3.74 (3H, s), OCH_3_ = 3.47 (3H, s), 3.45 (3H, s), 3.39 (3H, s), 3.35 (3H, s), Boc =1.41 (9H, s).


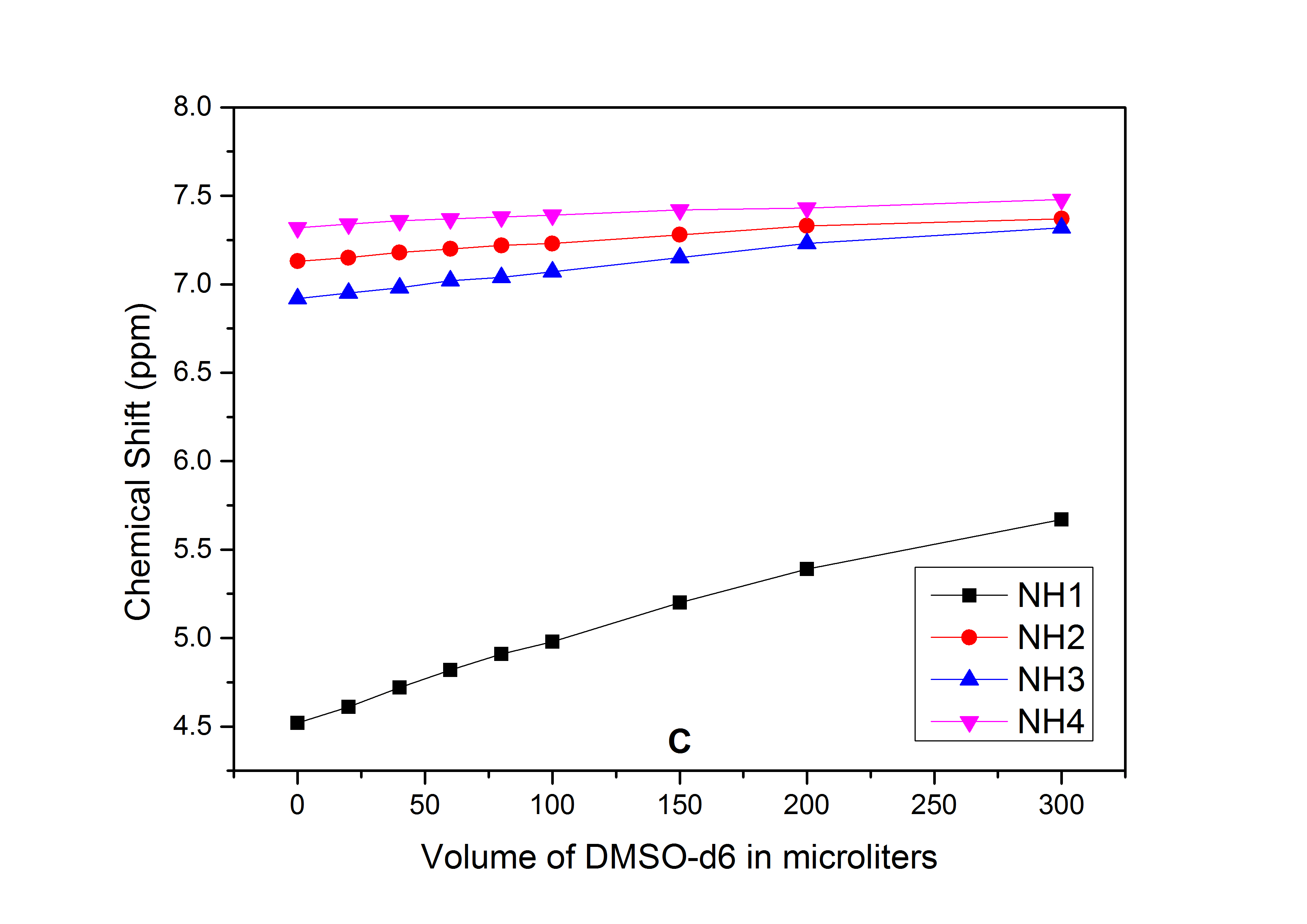


**Figure 3:** Solvent Titration plot of **23**

**^1^H NMR spectrum of 23 (700 MHz, CDCl_3_, 298K)**

**
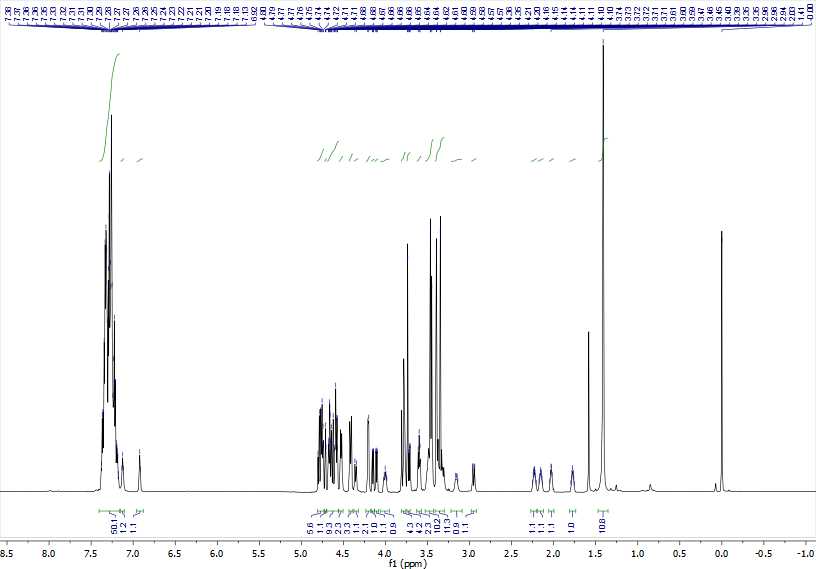
**

**TOCSY spectrum of 23 (700 MHz, CDCl_3_, 298K)**

**
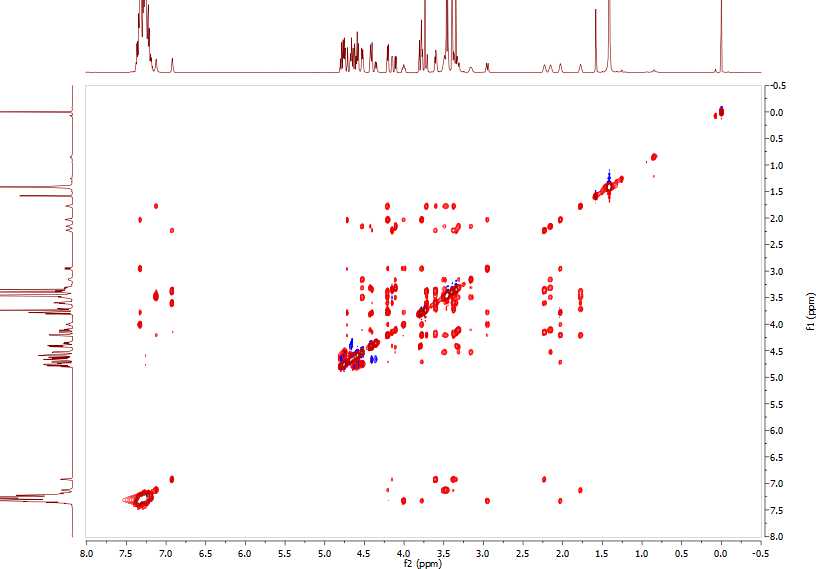
**

**ROESY spectrum of 23 (700 MHz, CDCl_3_, 298K)**

**
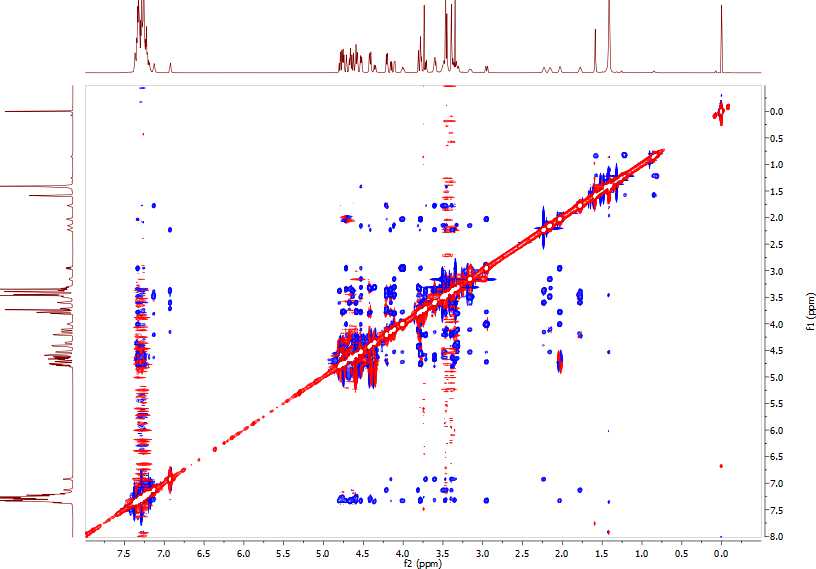
**

**4. NMR spectra**


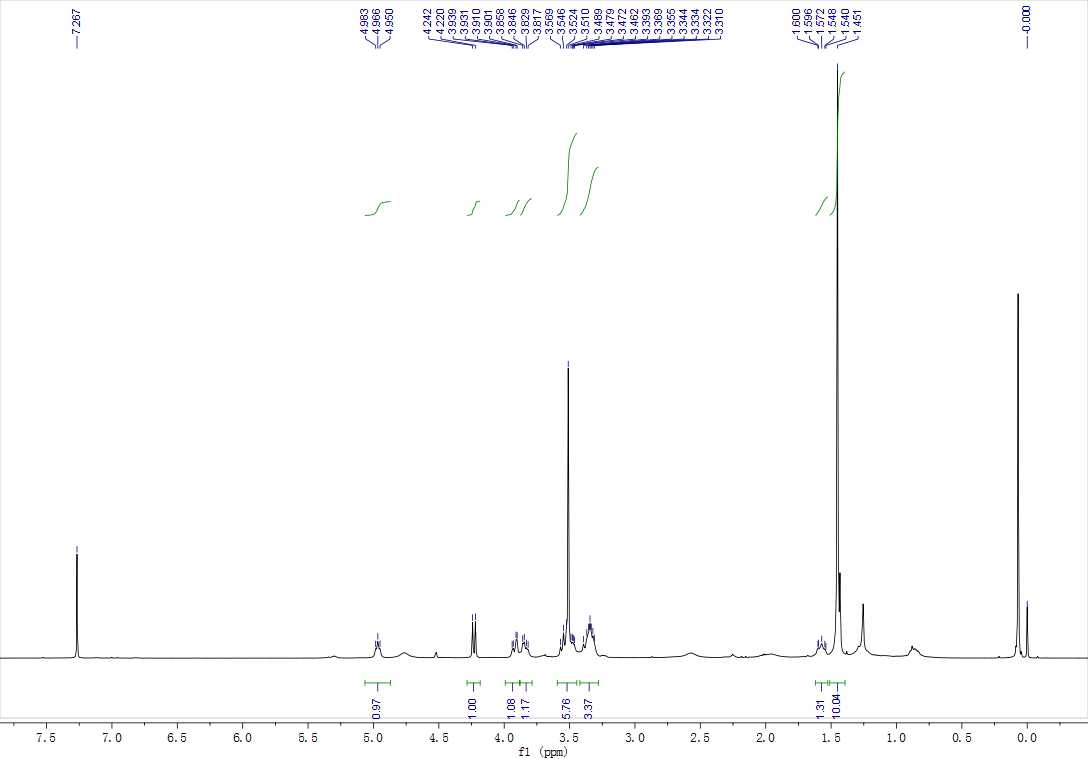


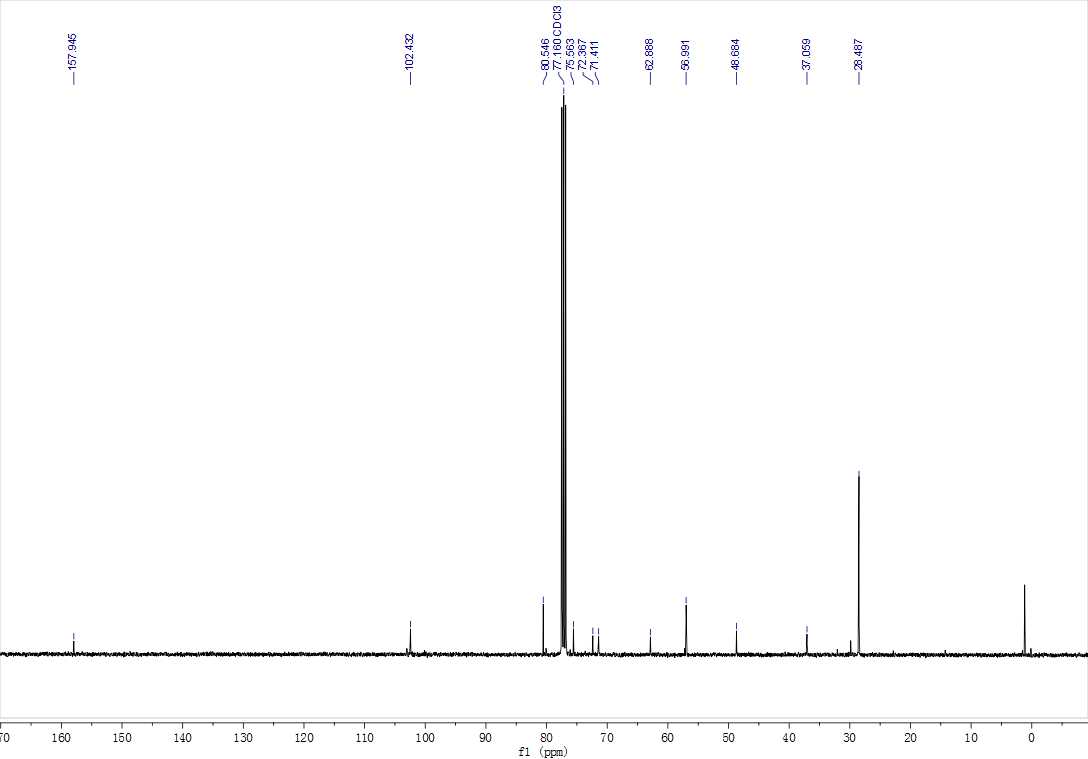


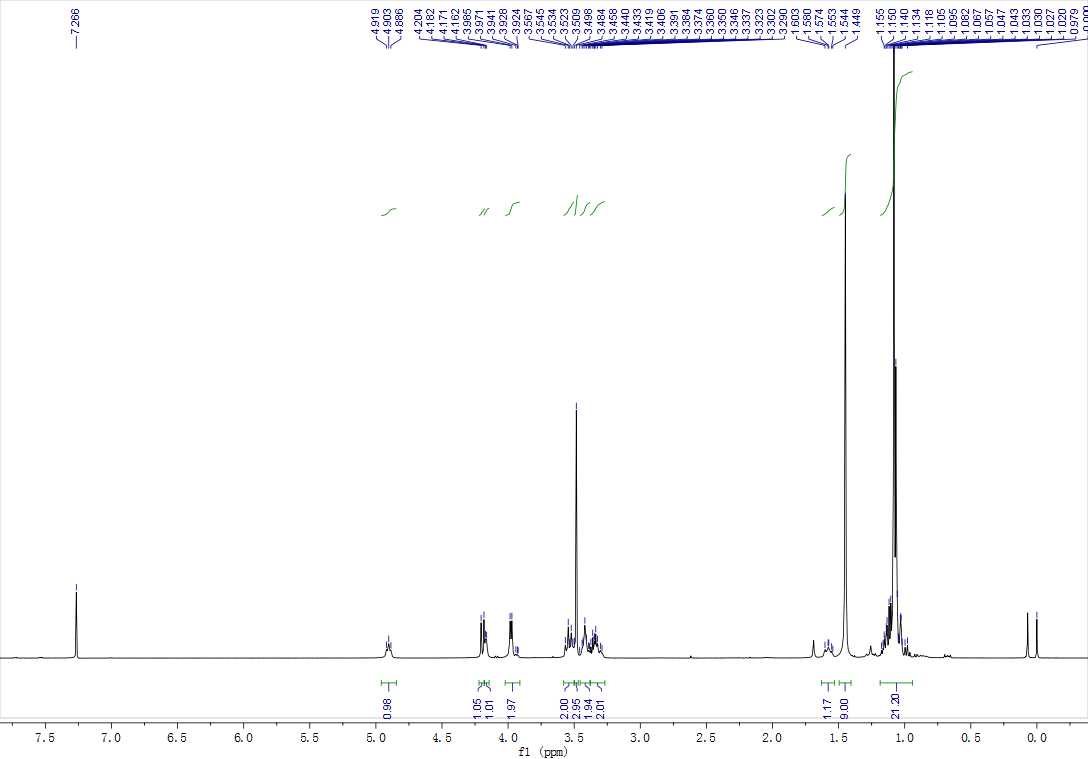


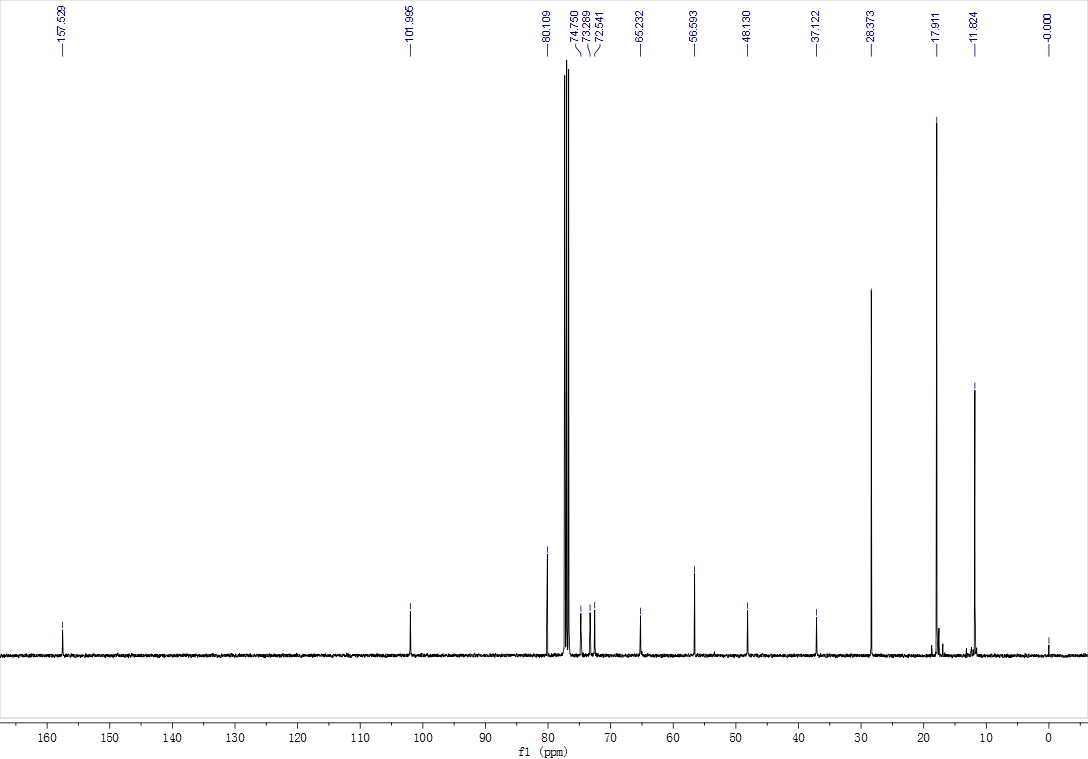


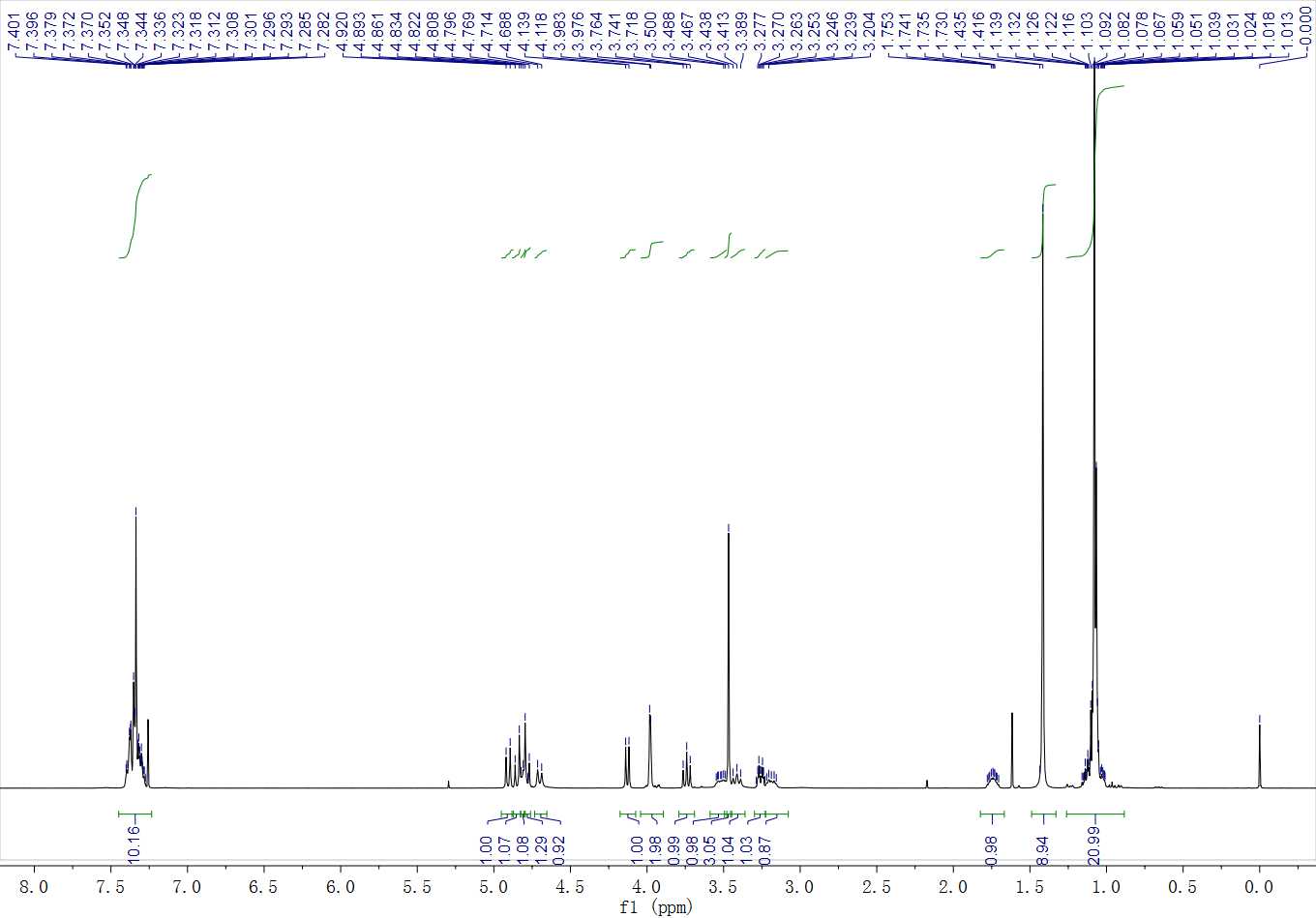


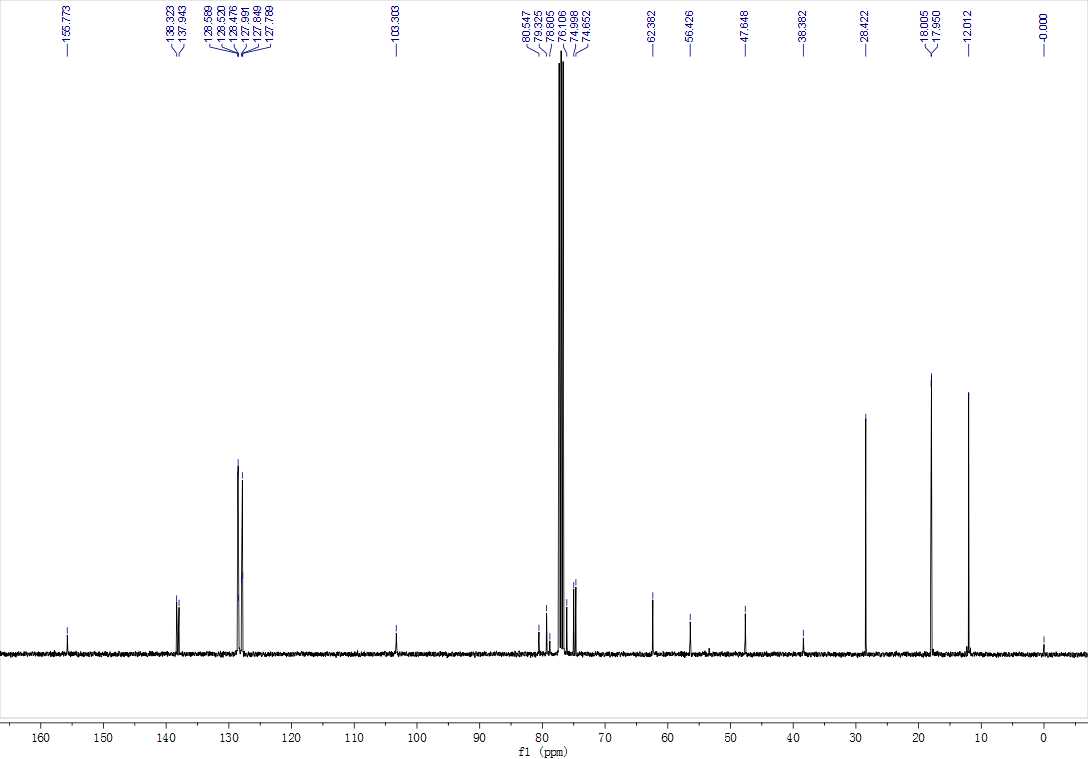


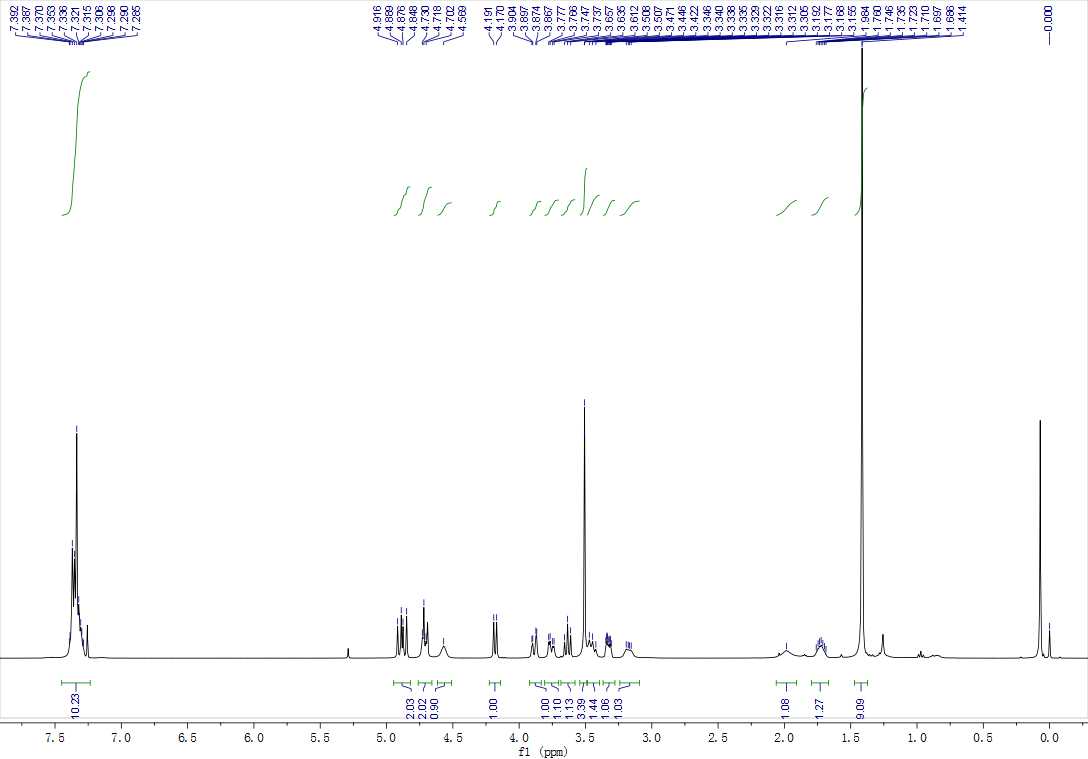


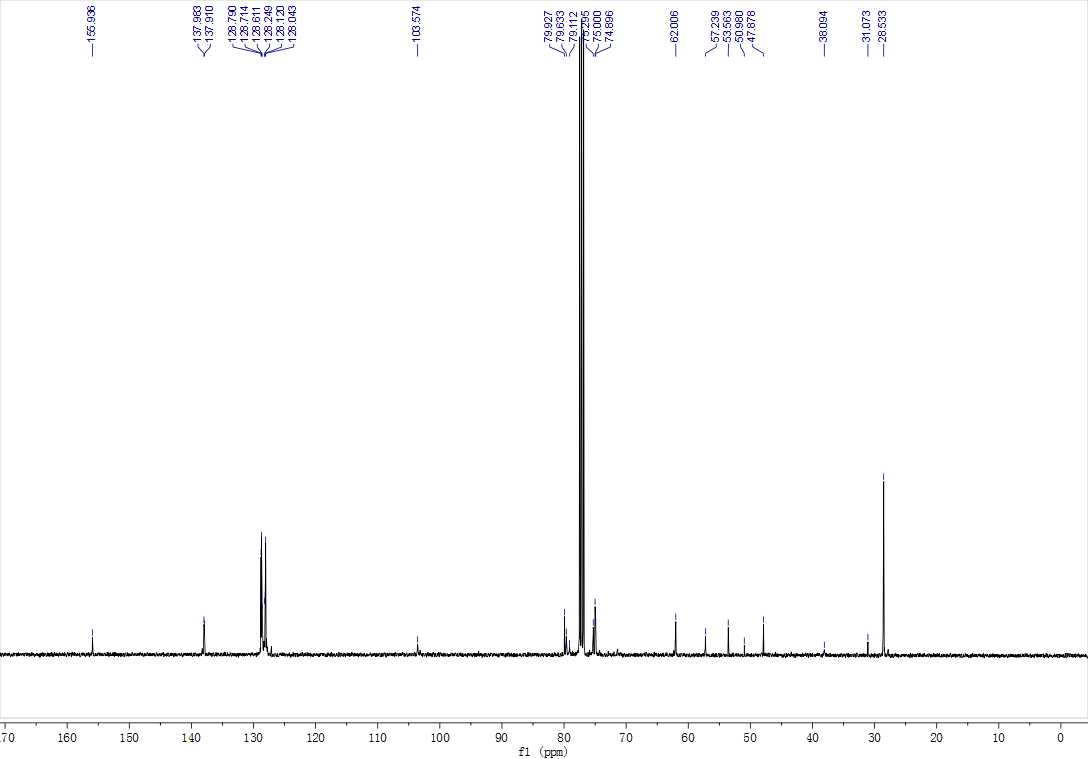


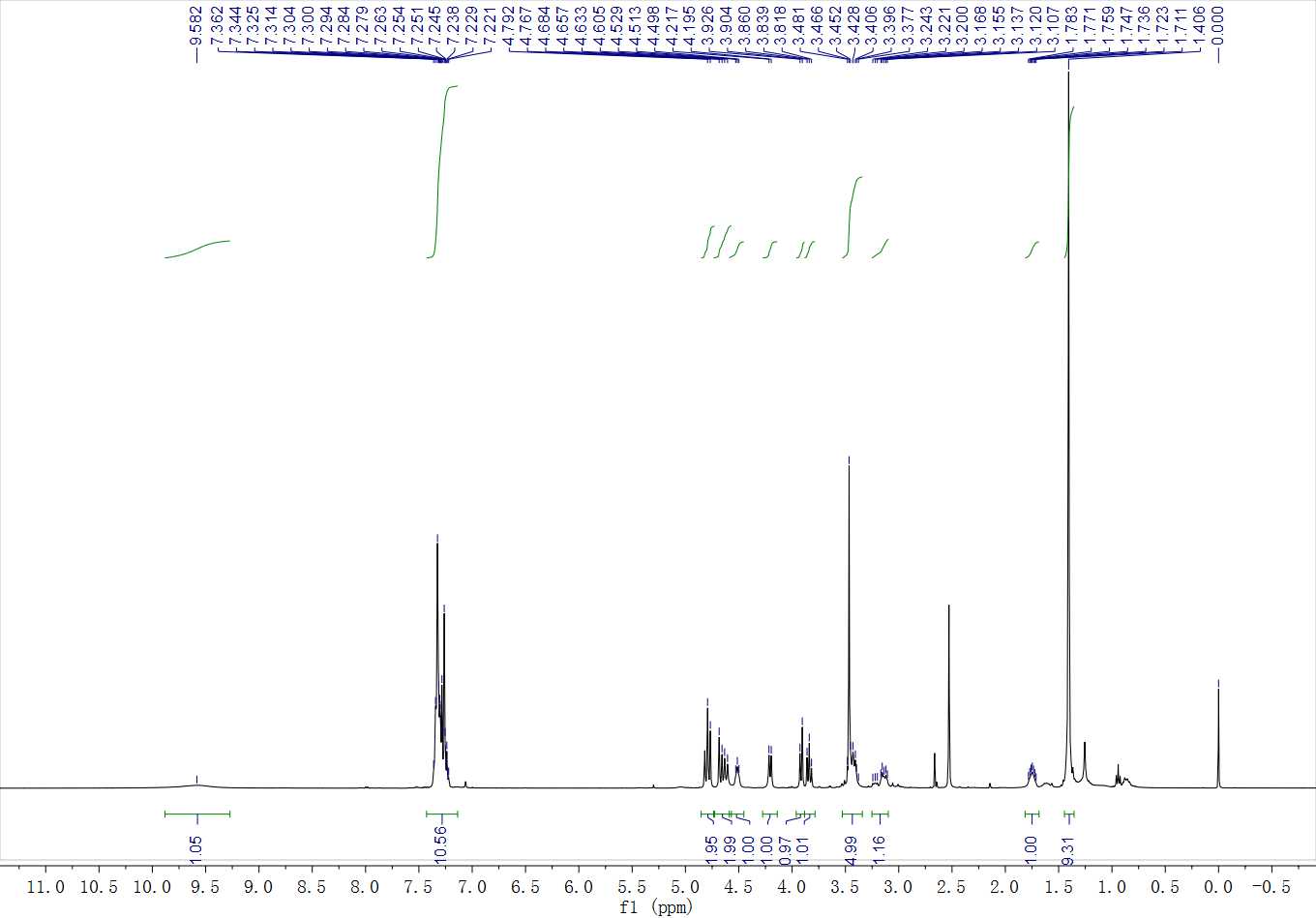


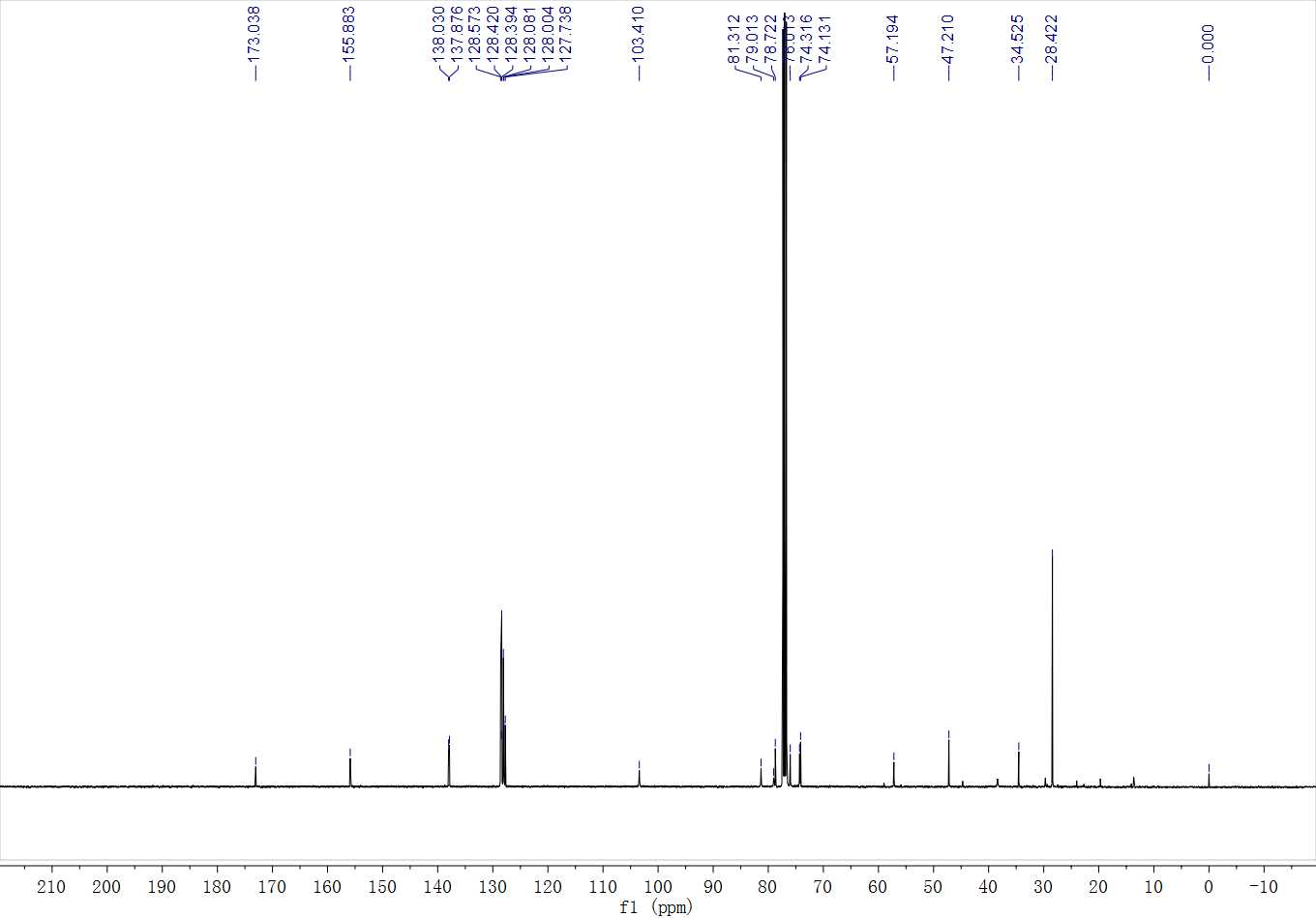


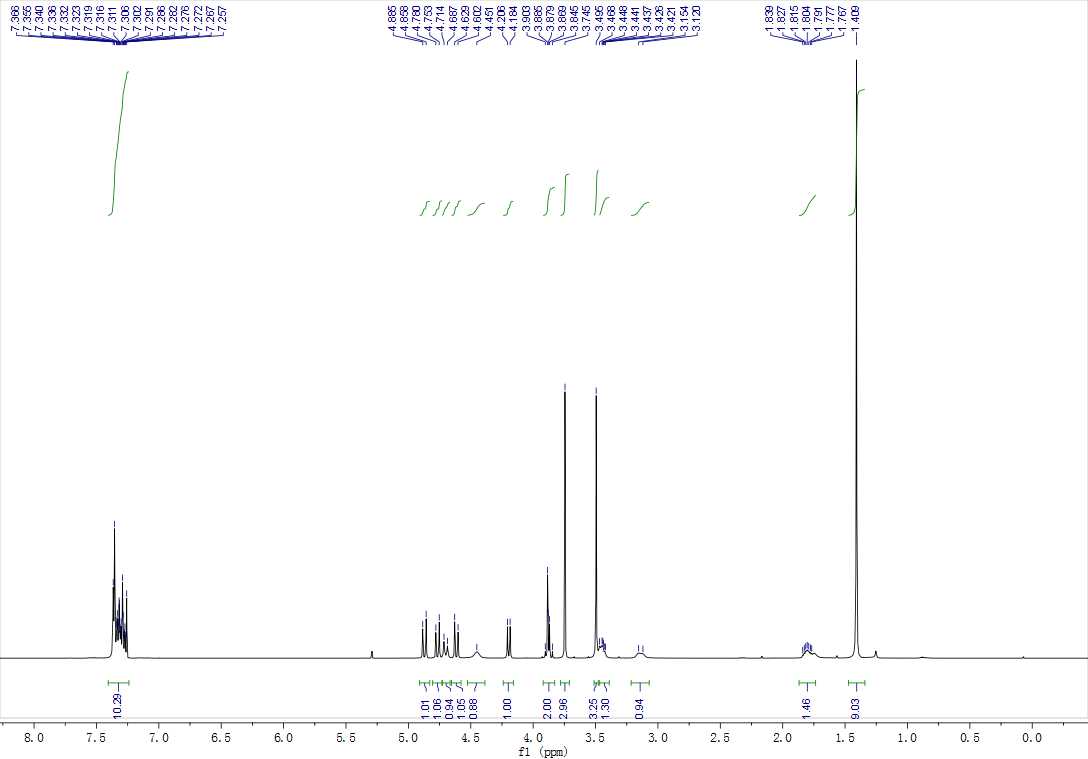


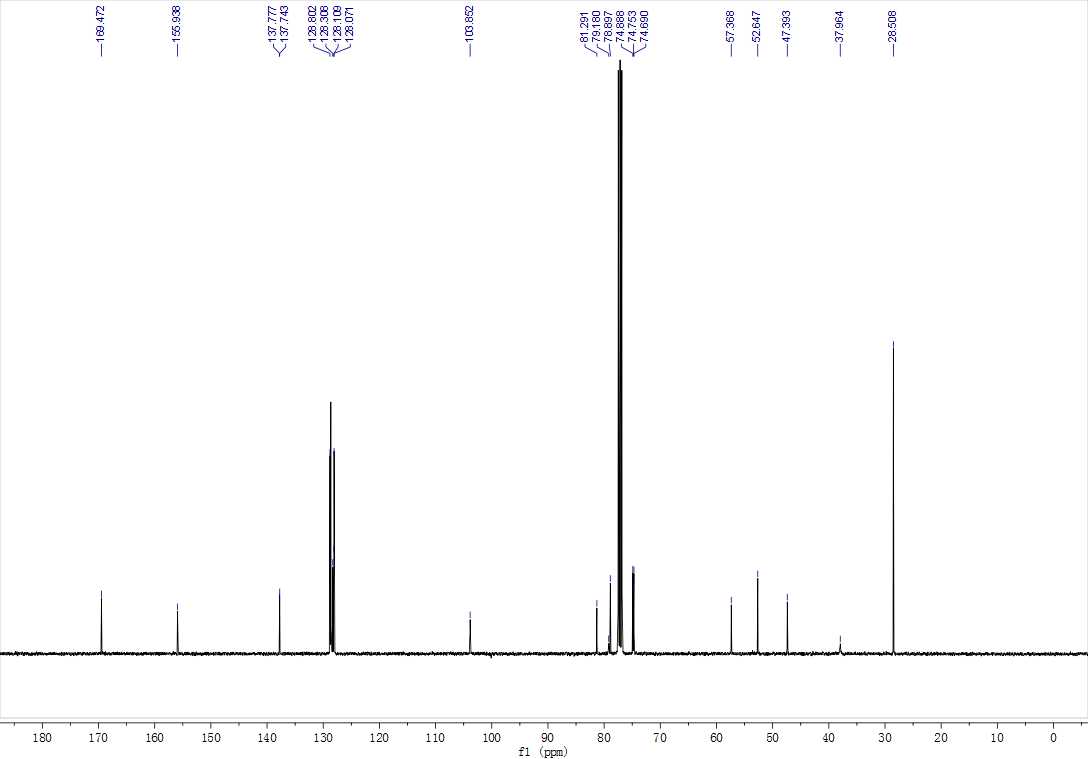


**
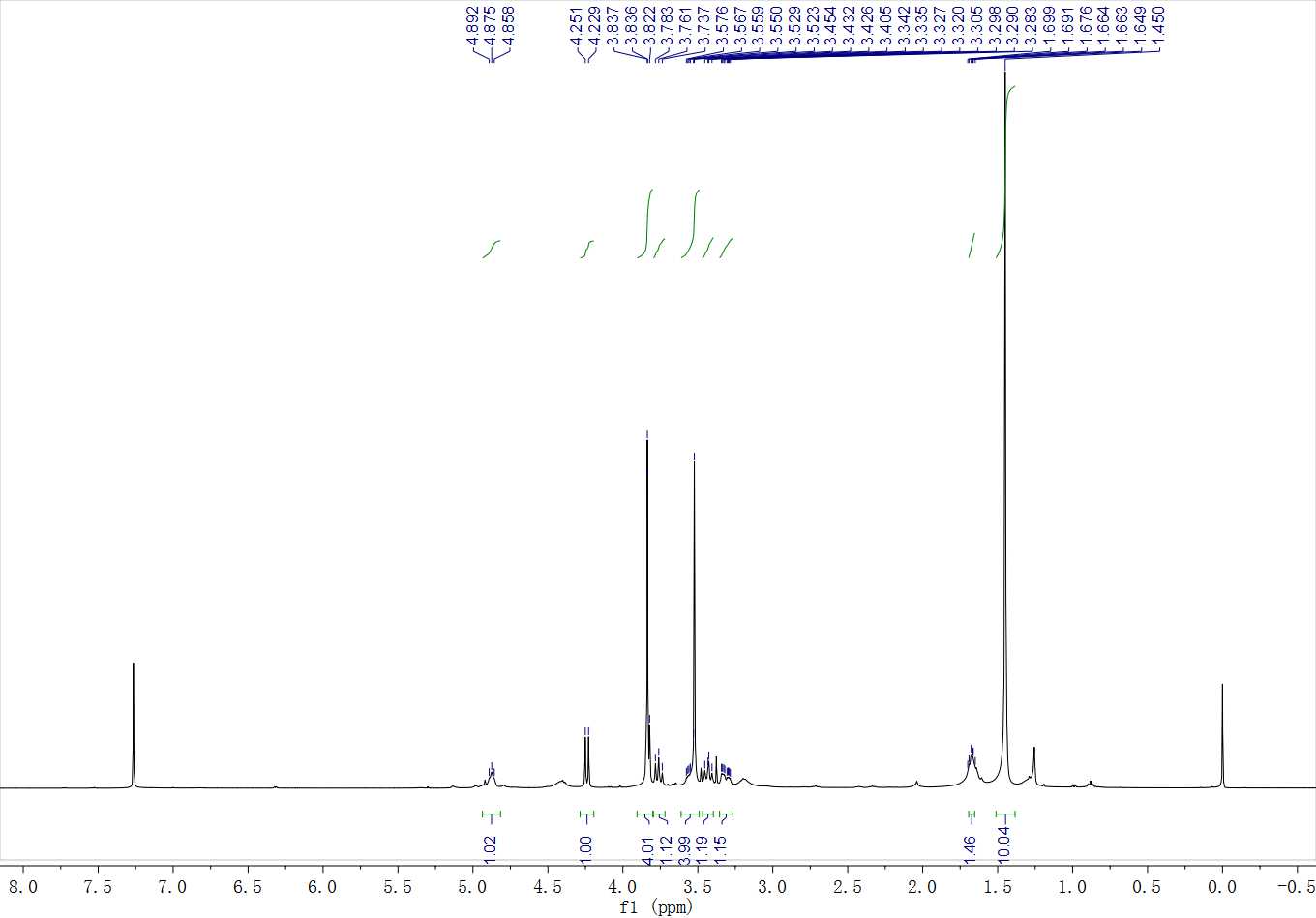
**

**
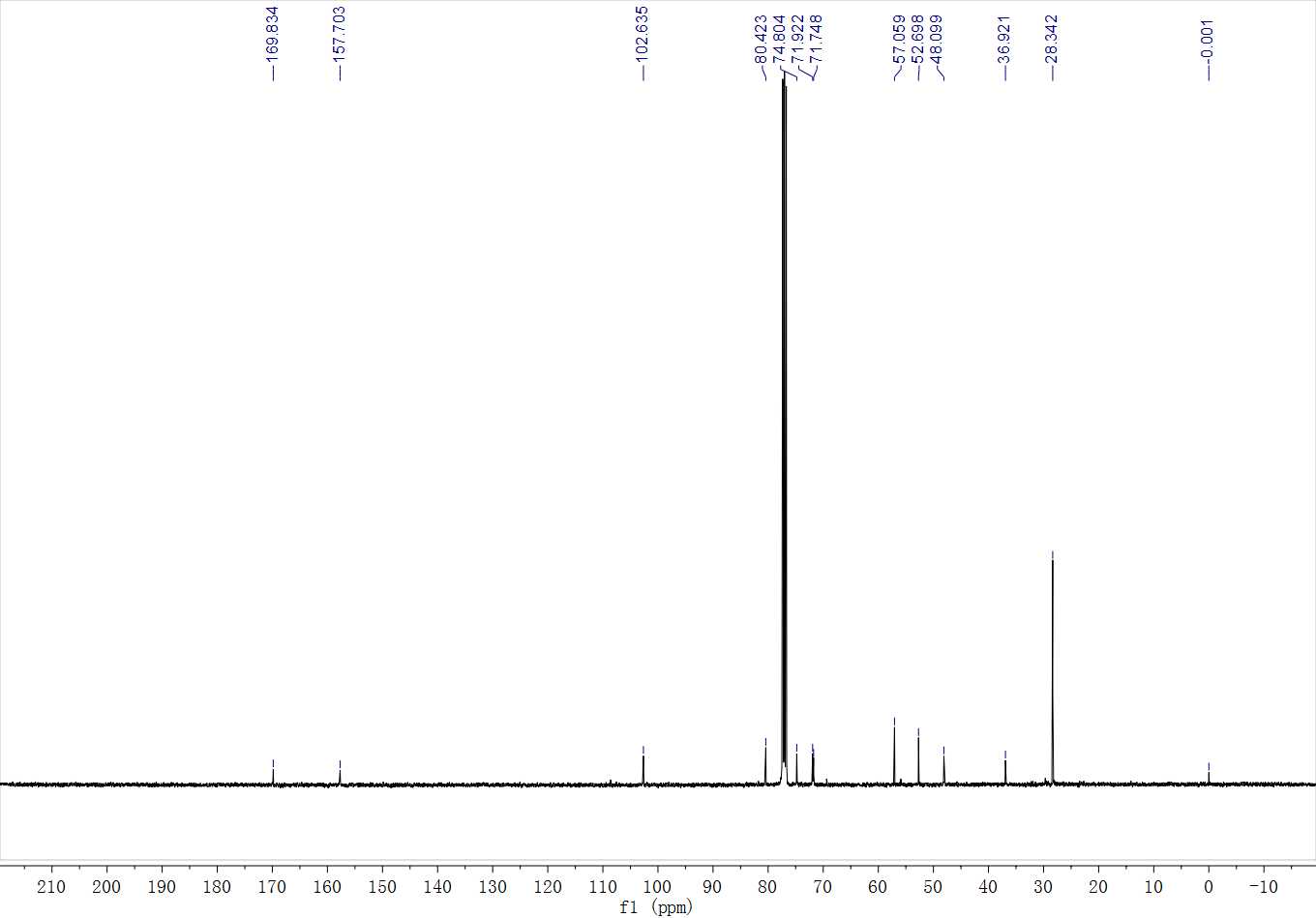
**


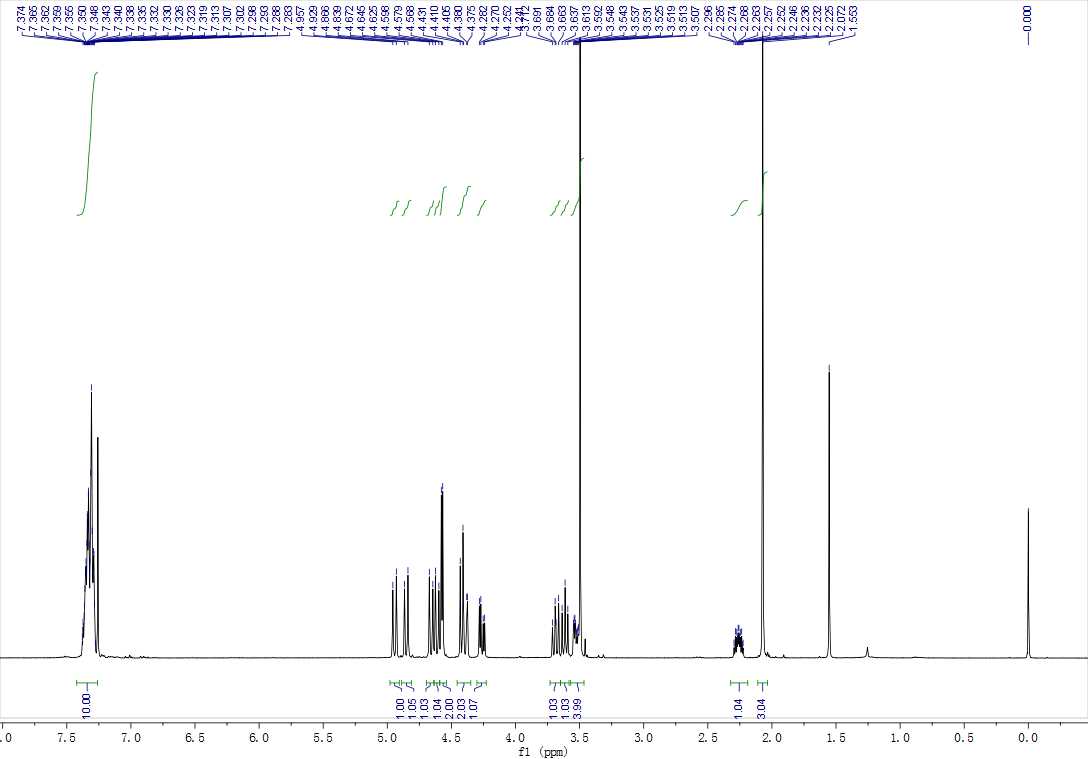


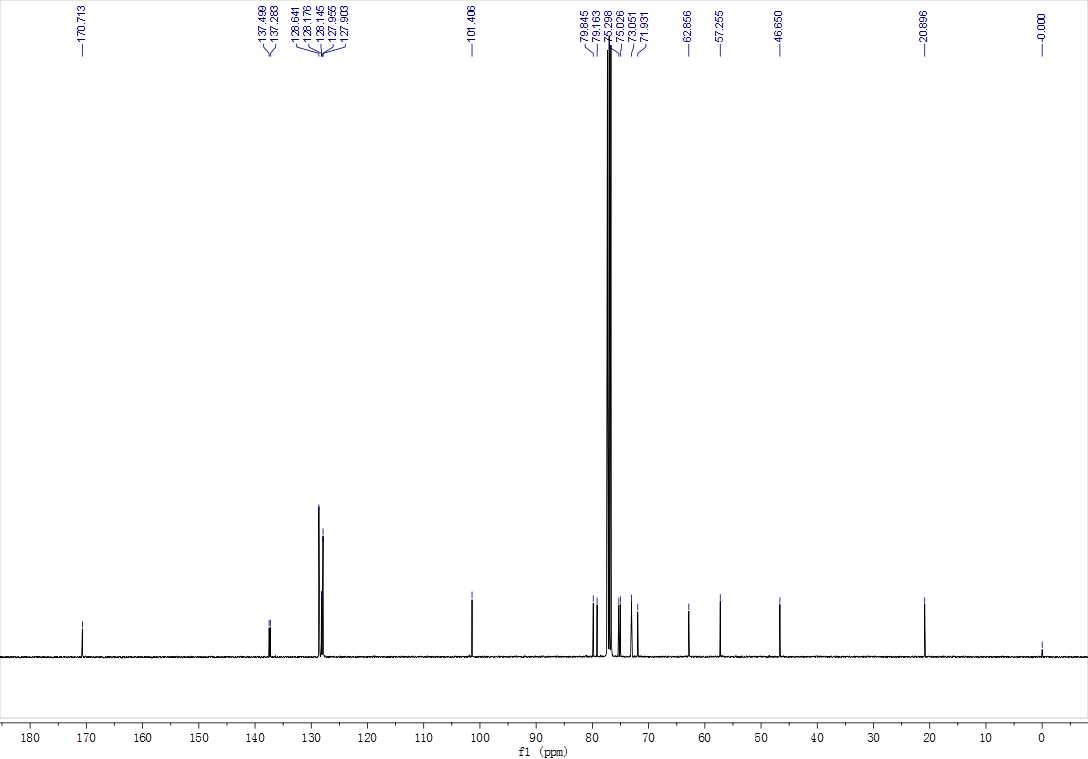


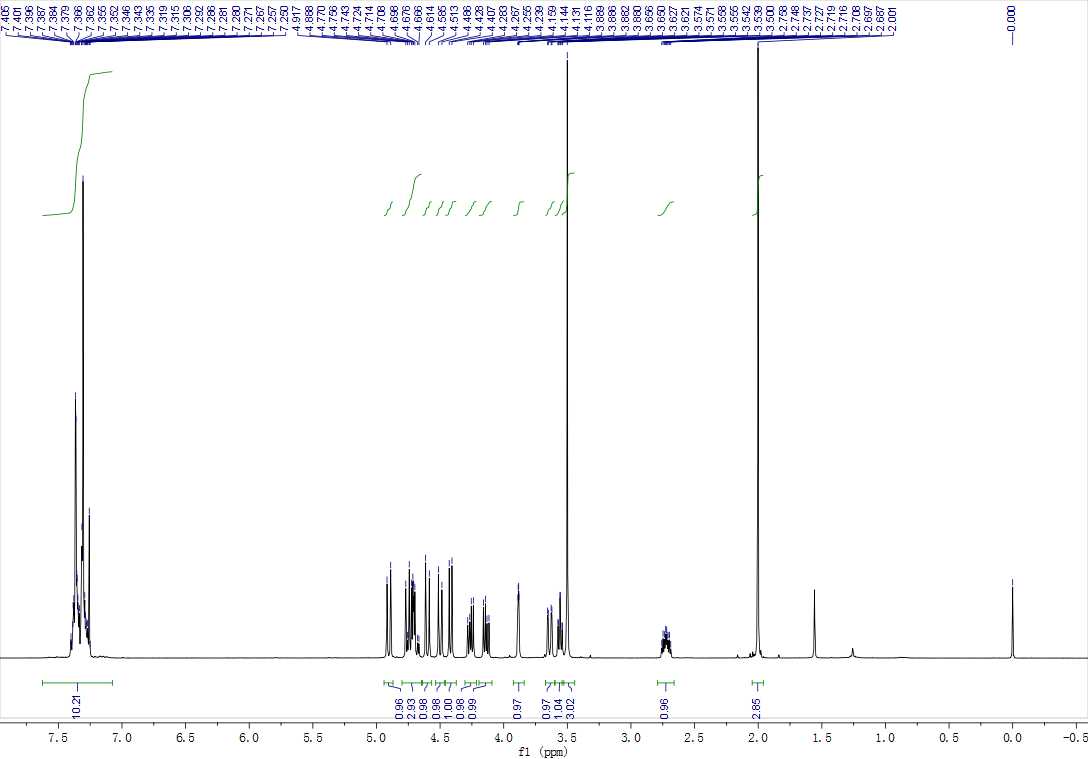


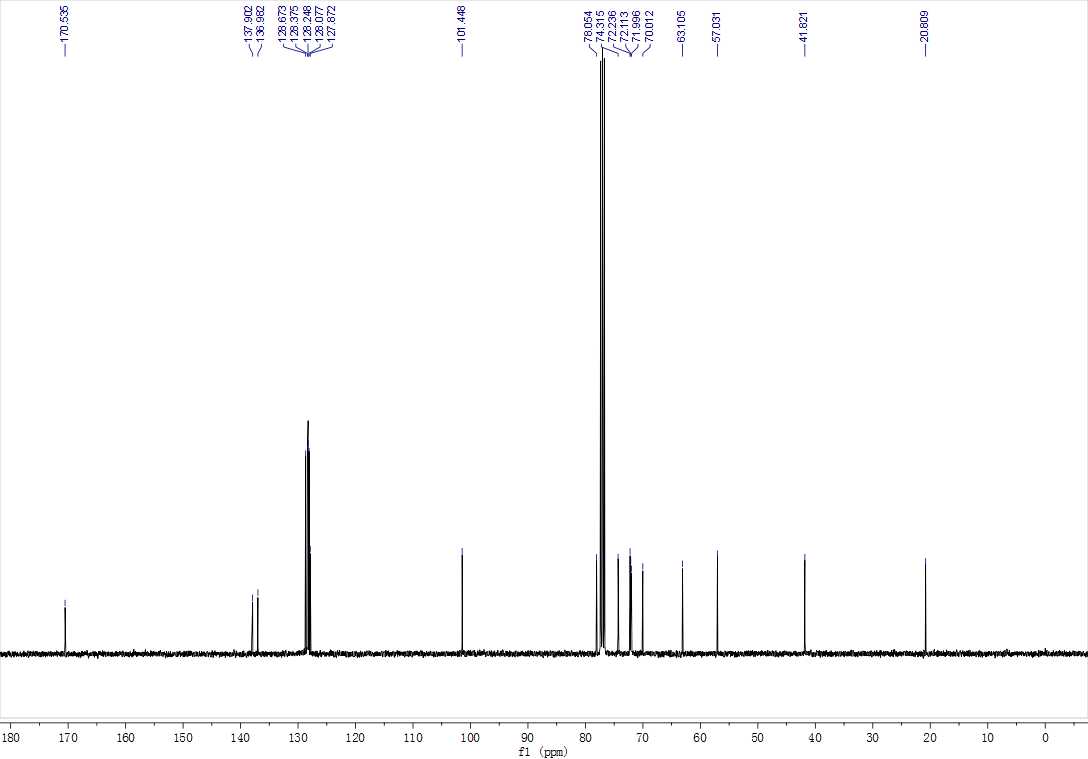


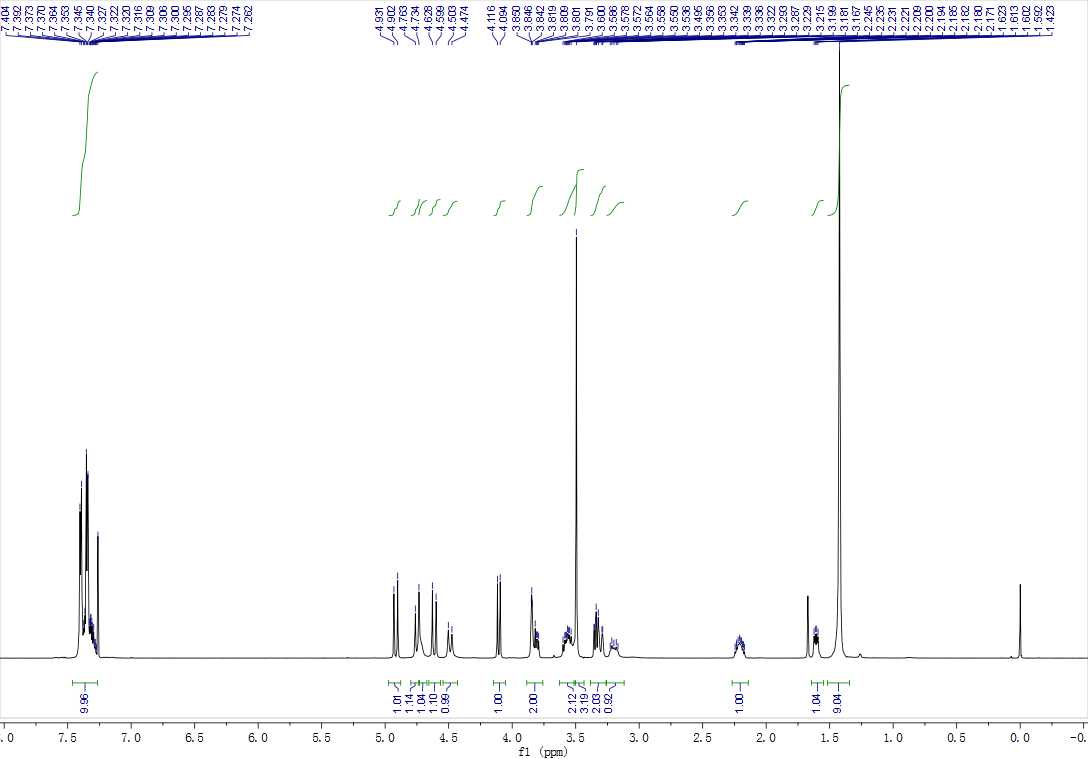


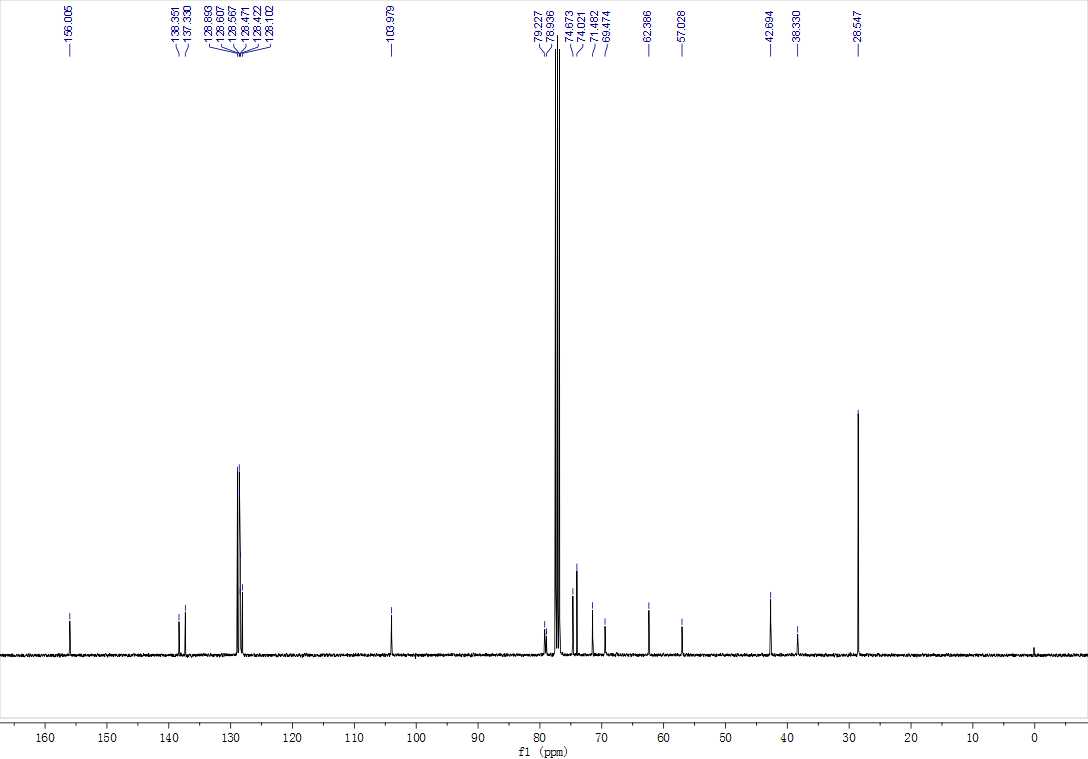


**
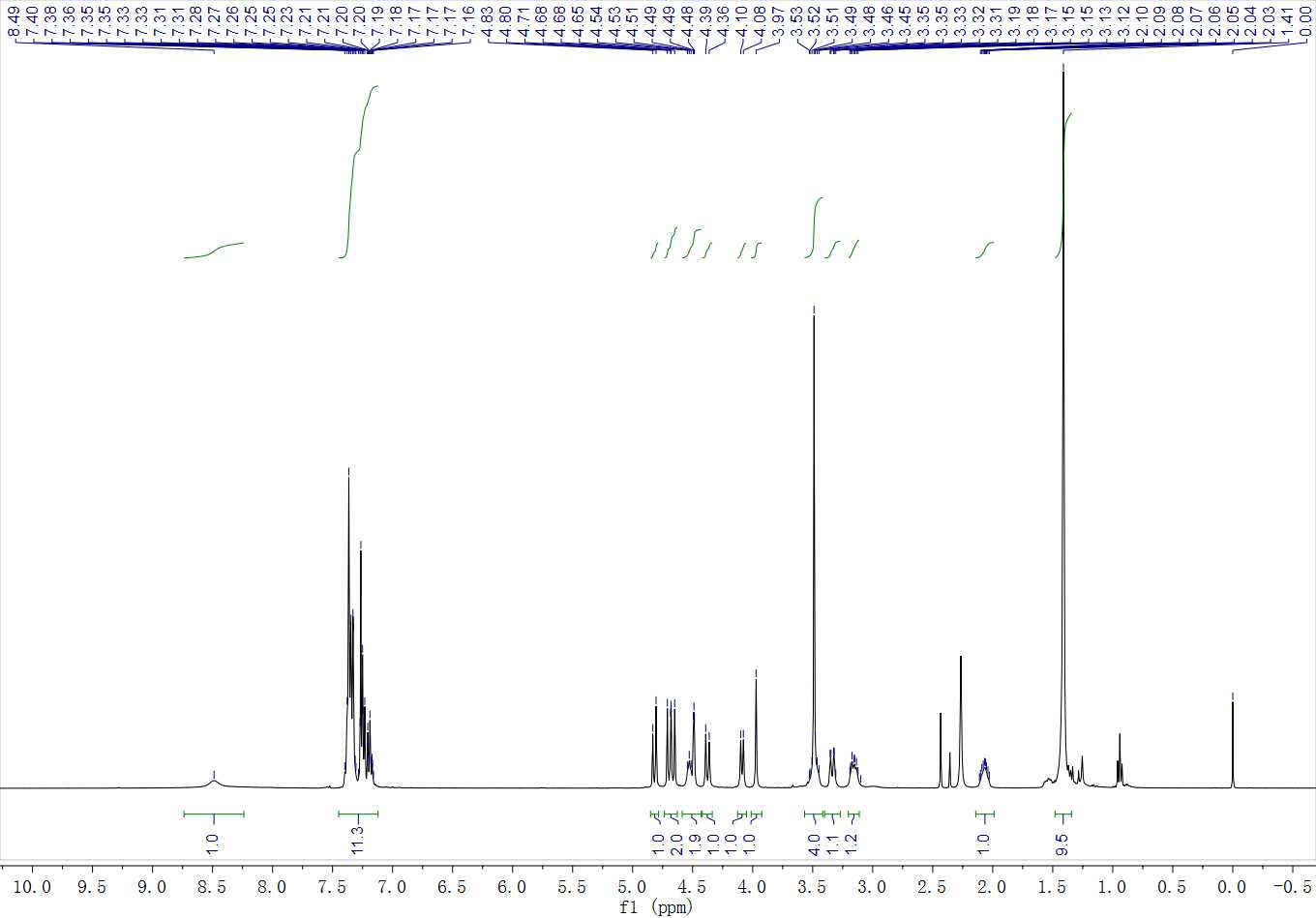
**

**
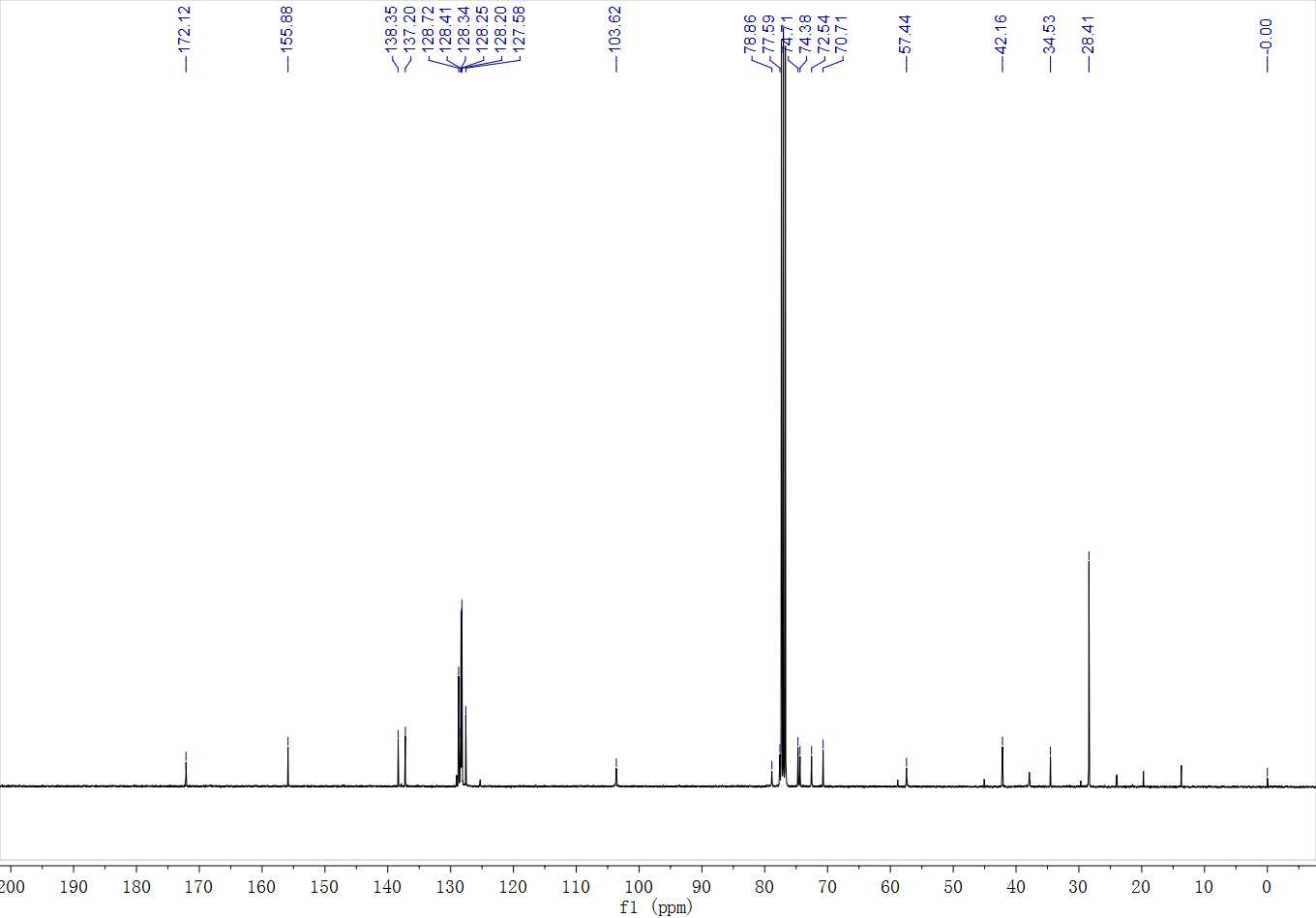
**


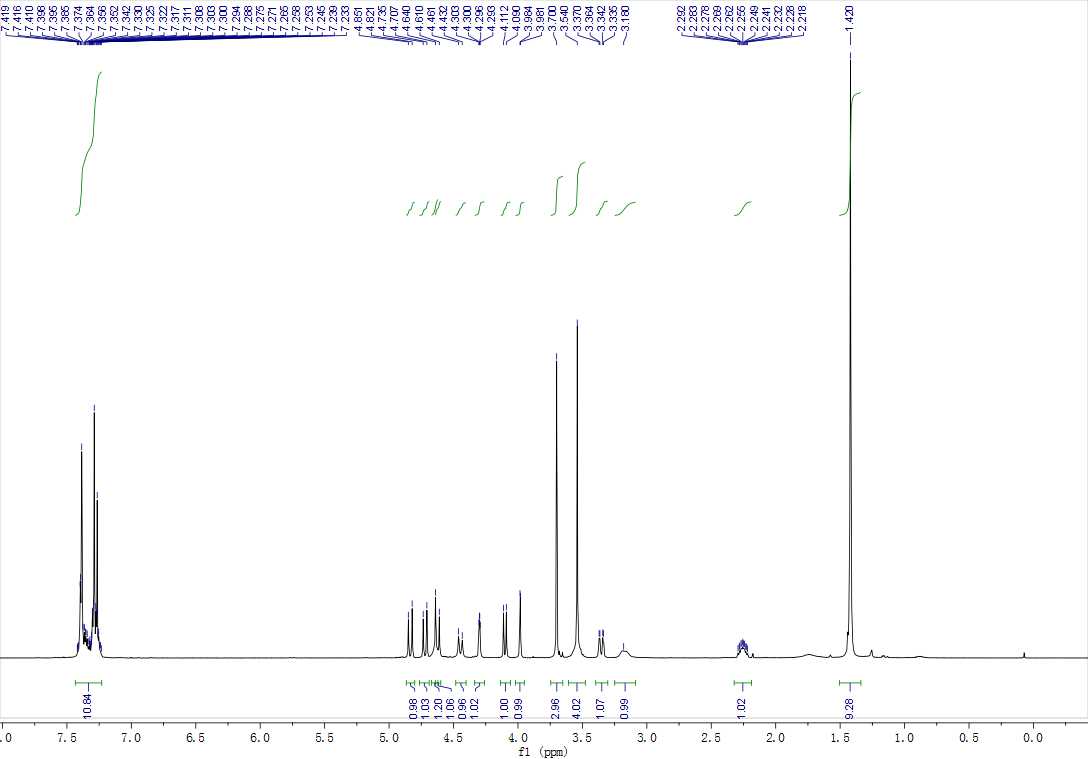


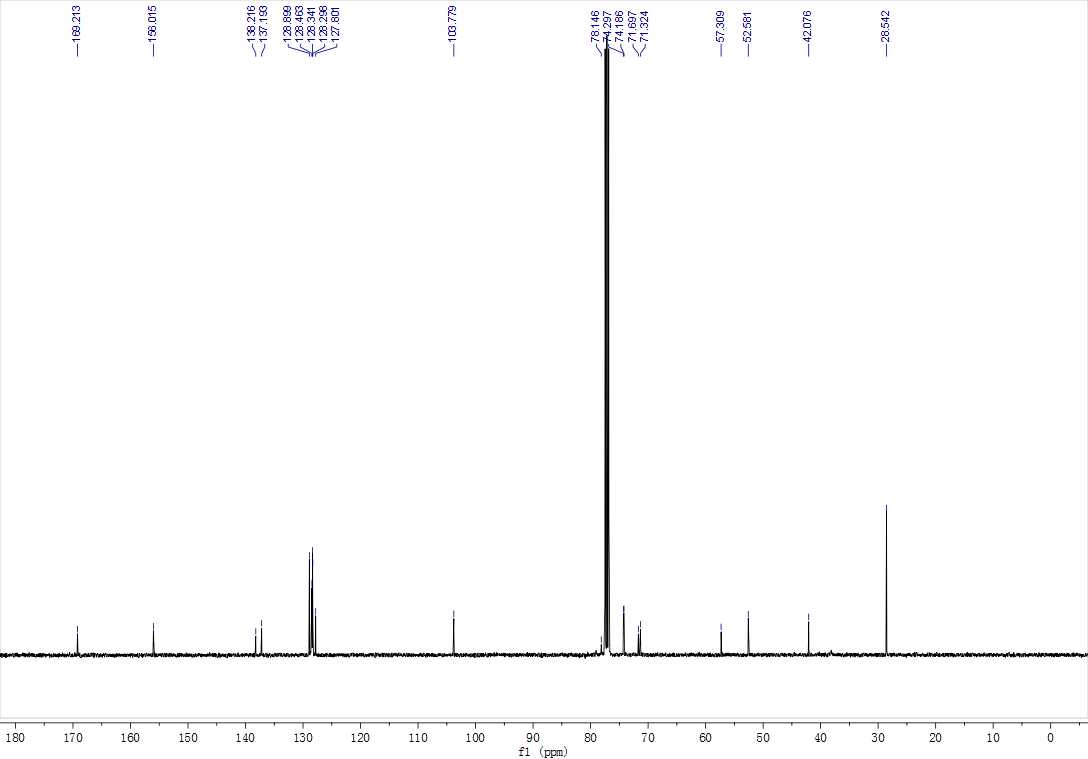


**
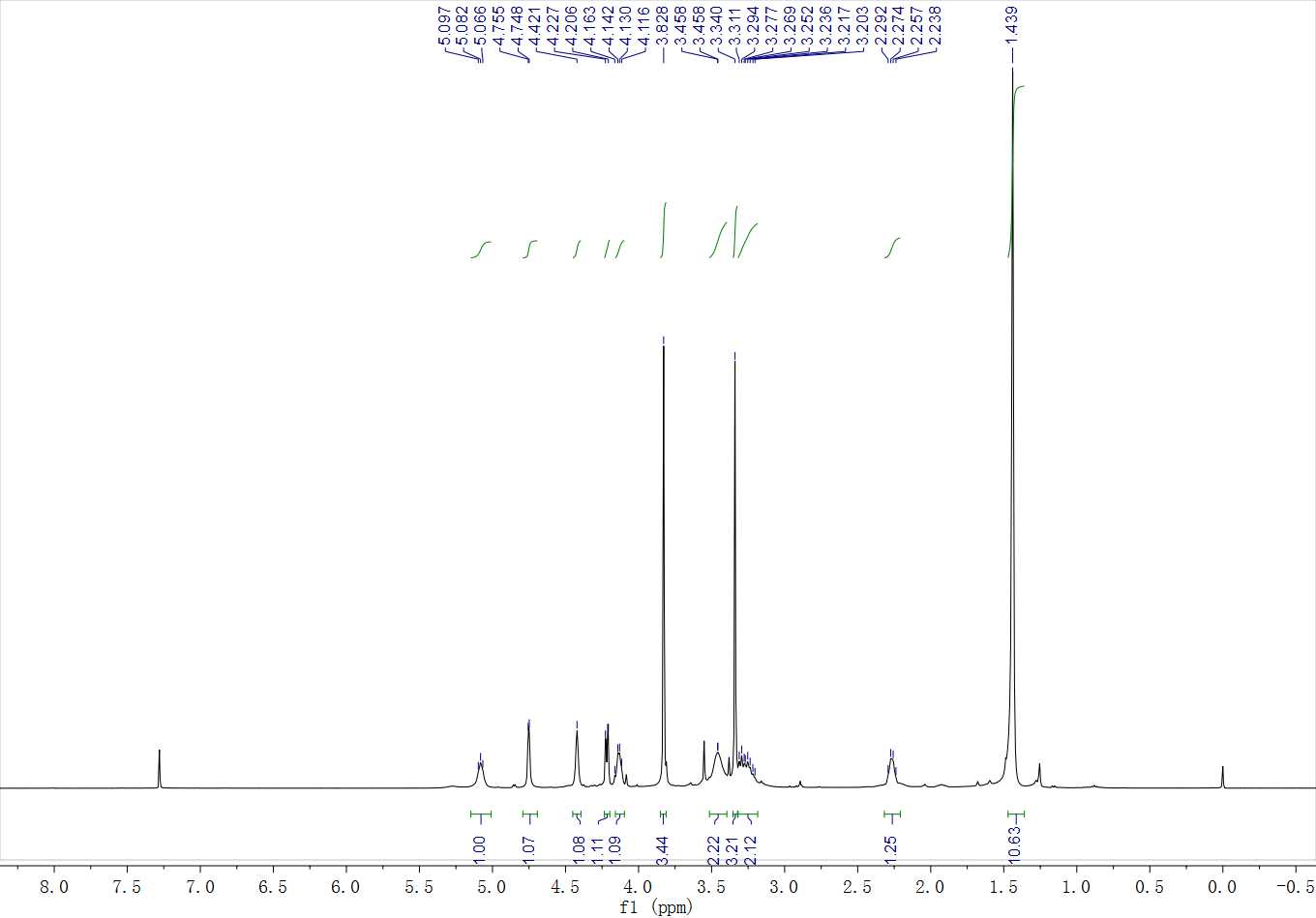
**

**
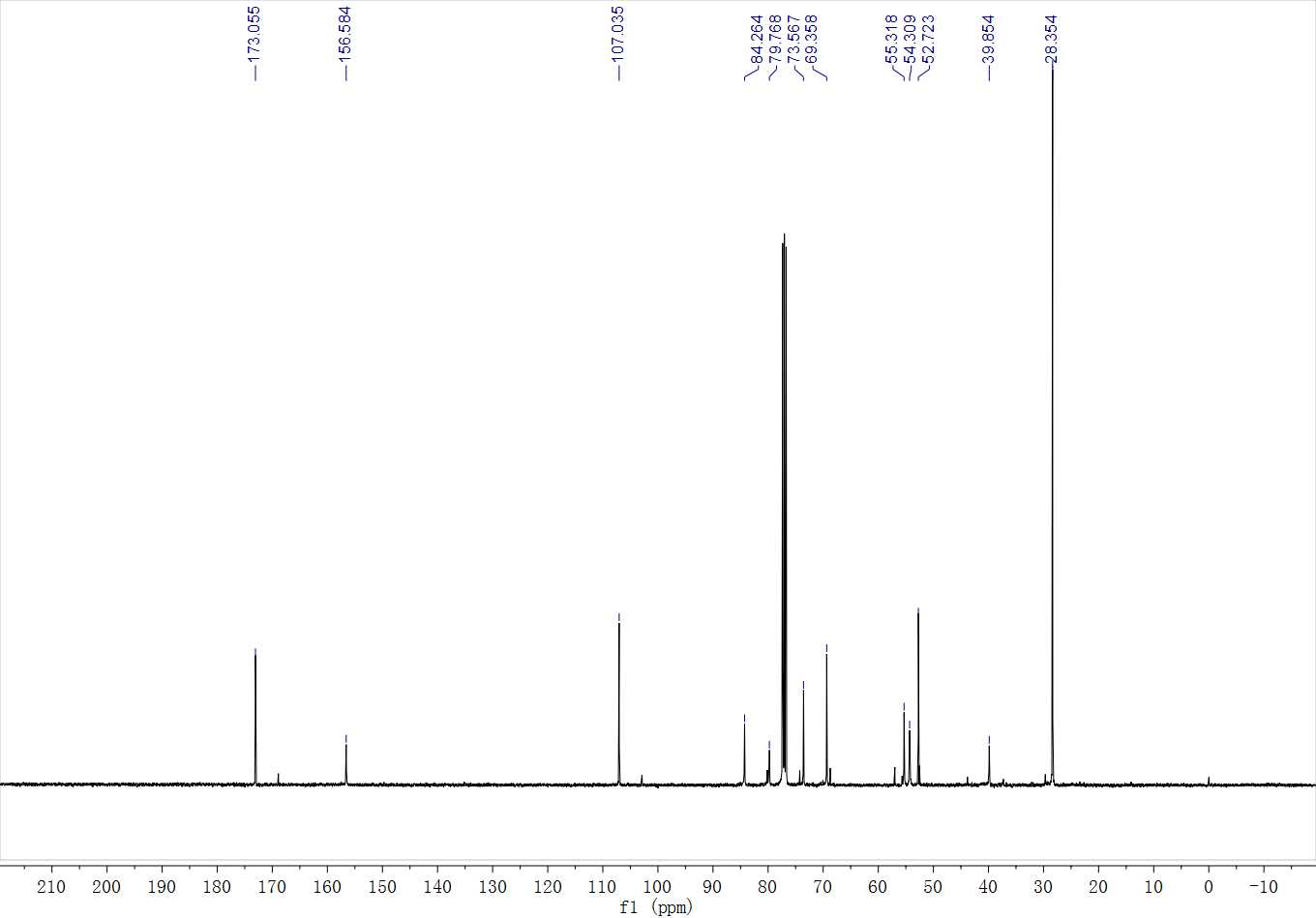
**

**
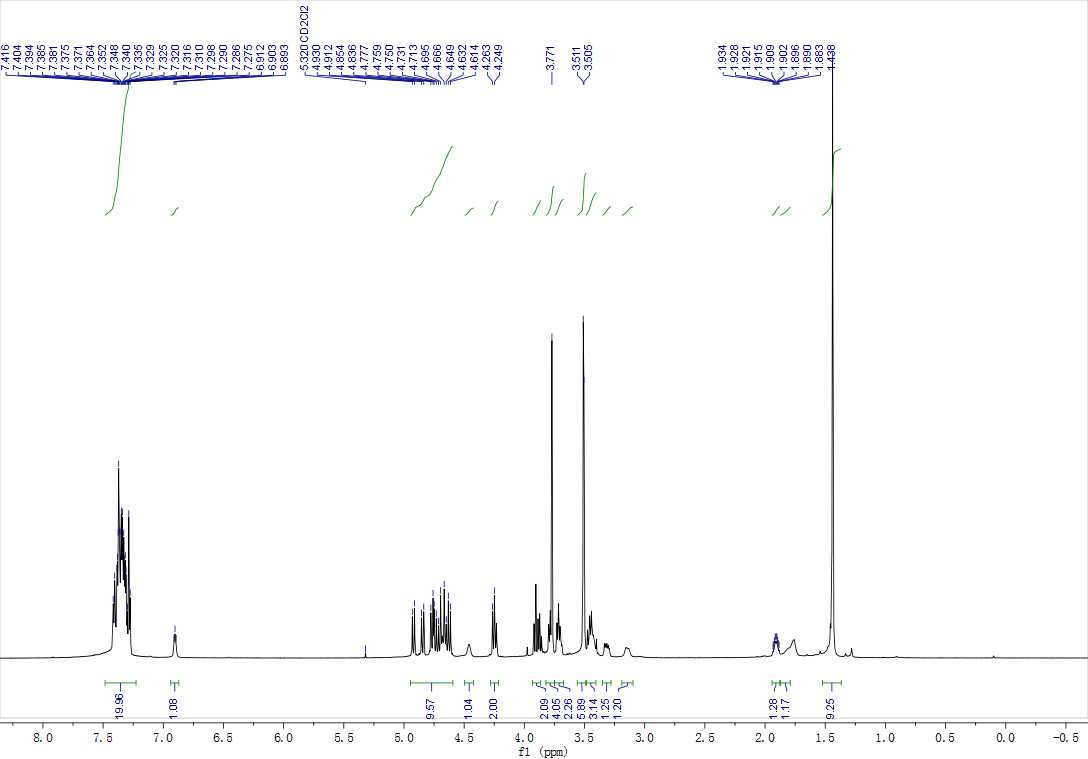
**

**
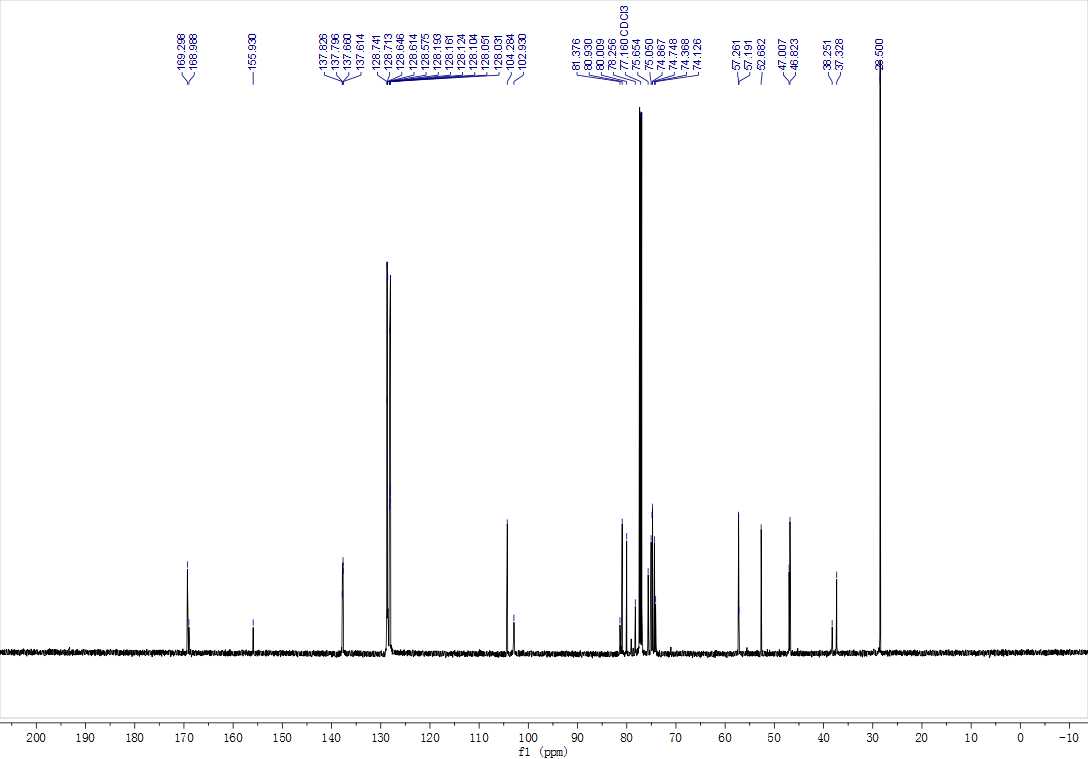
**

**
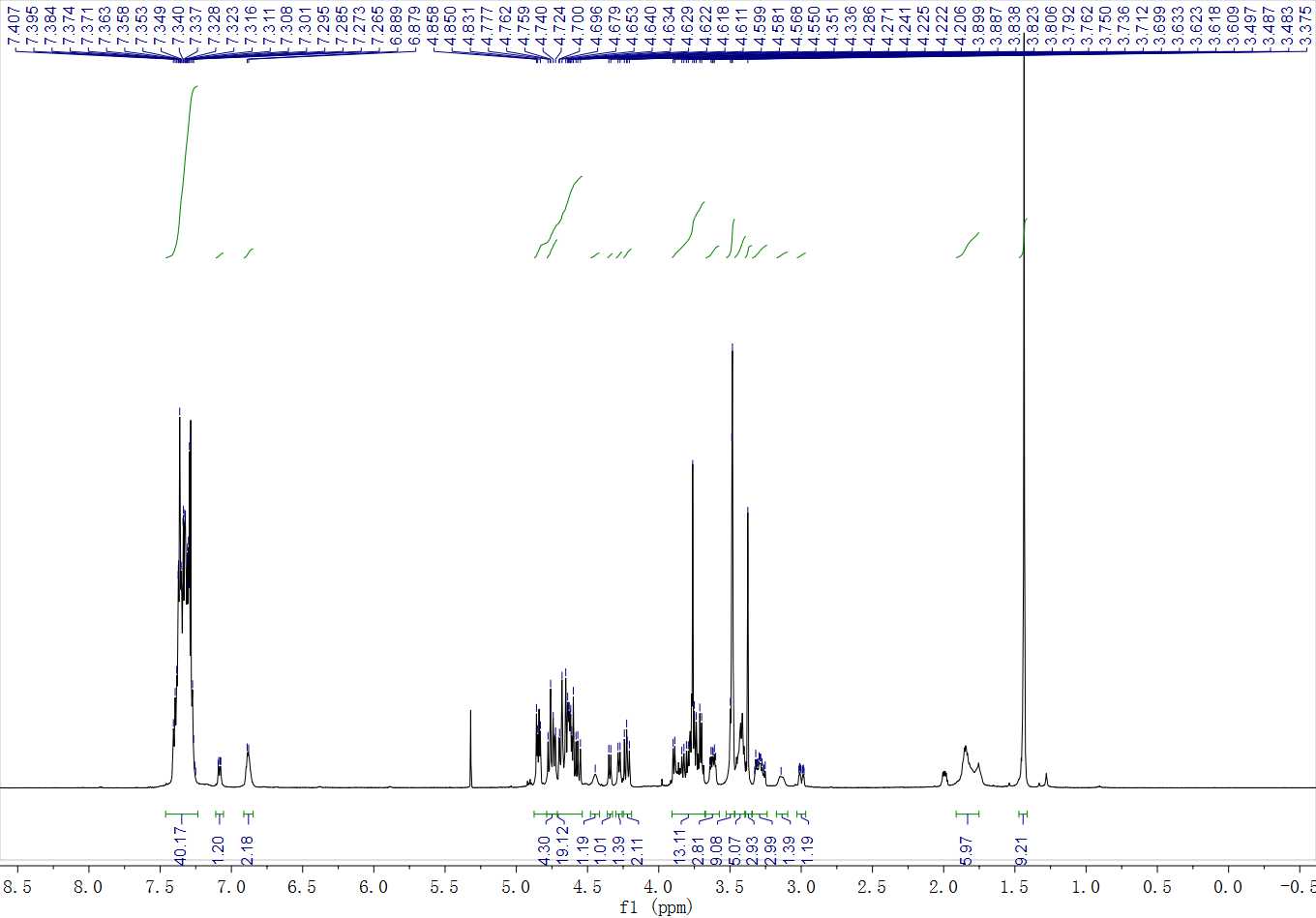
**

**
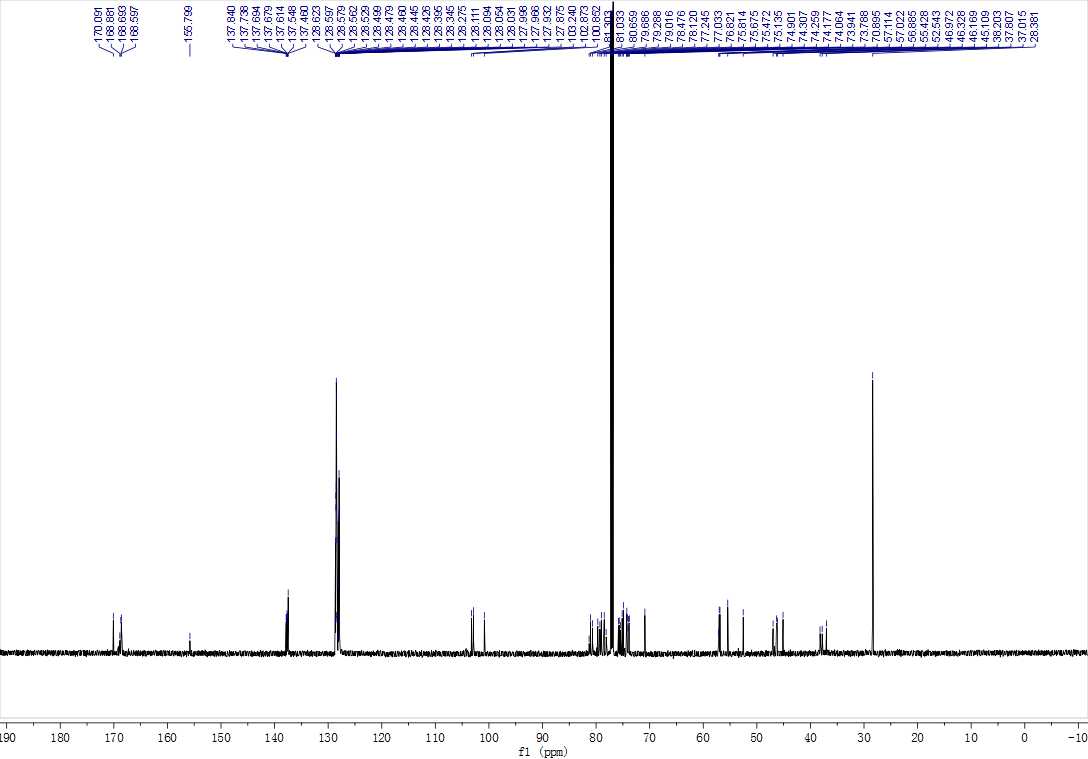
**

**
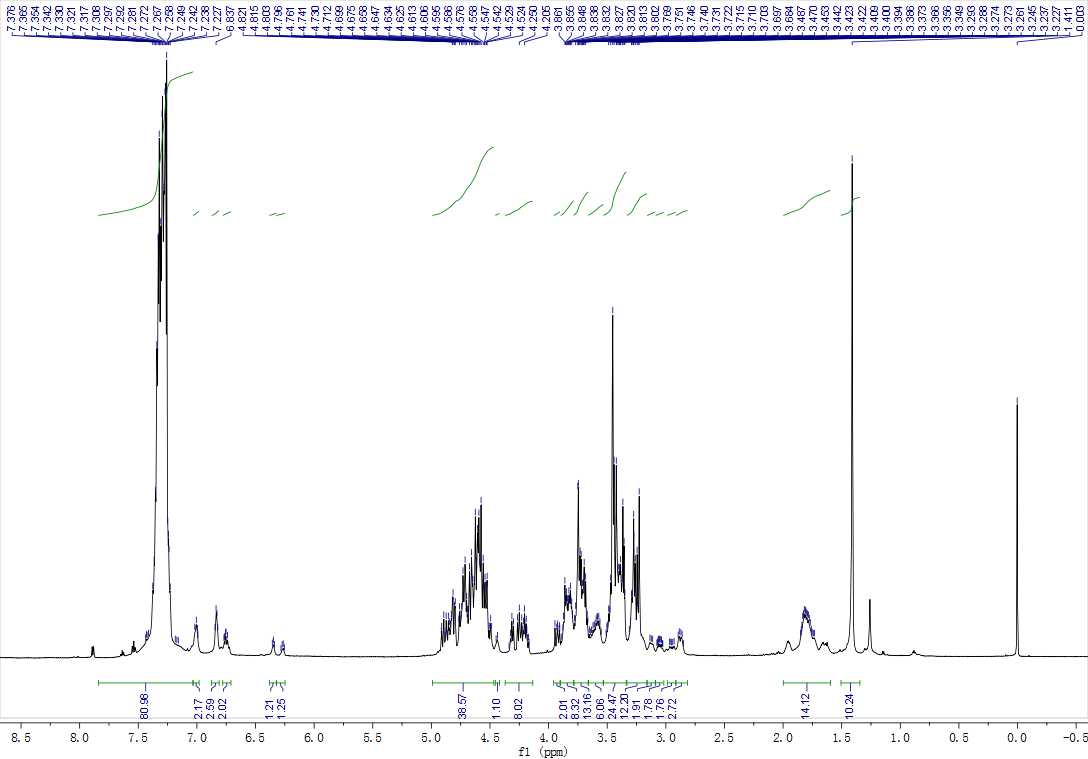
**

**
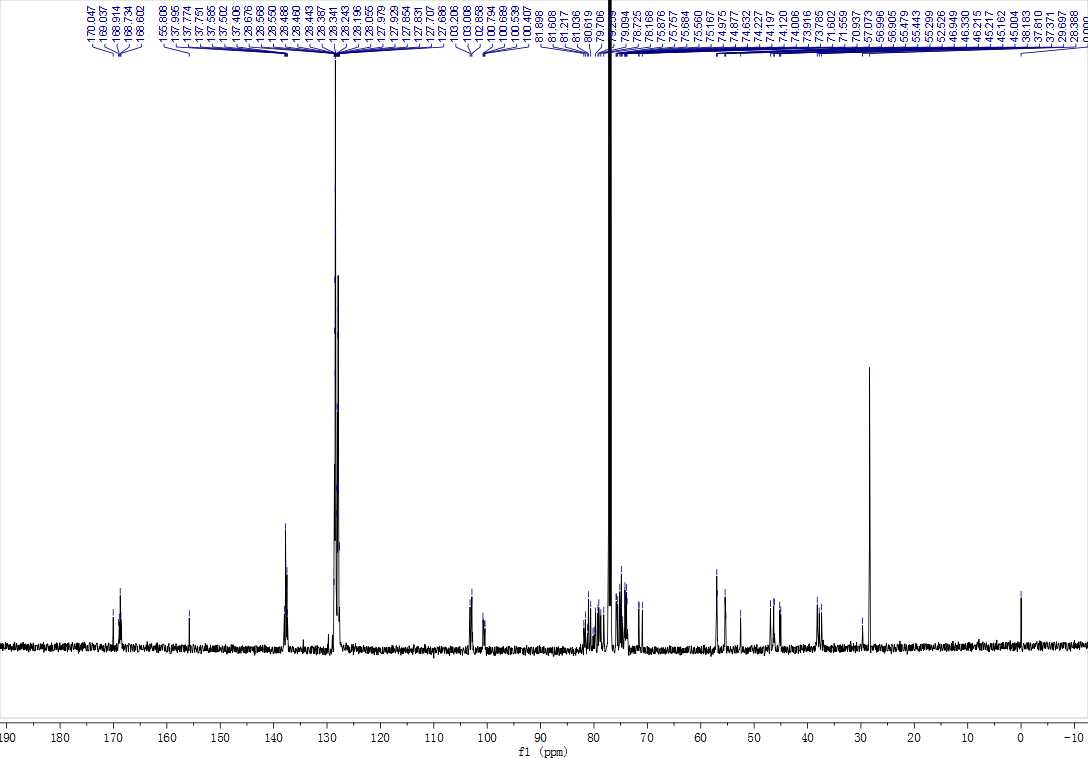
**

**
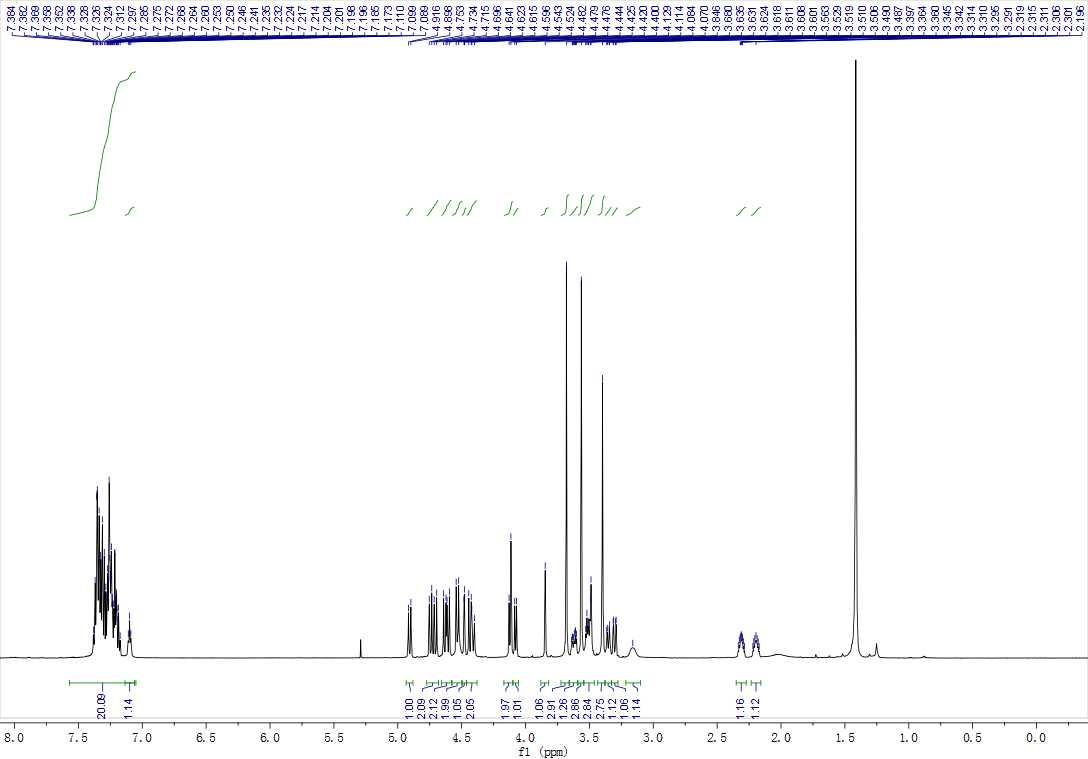
**

**
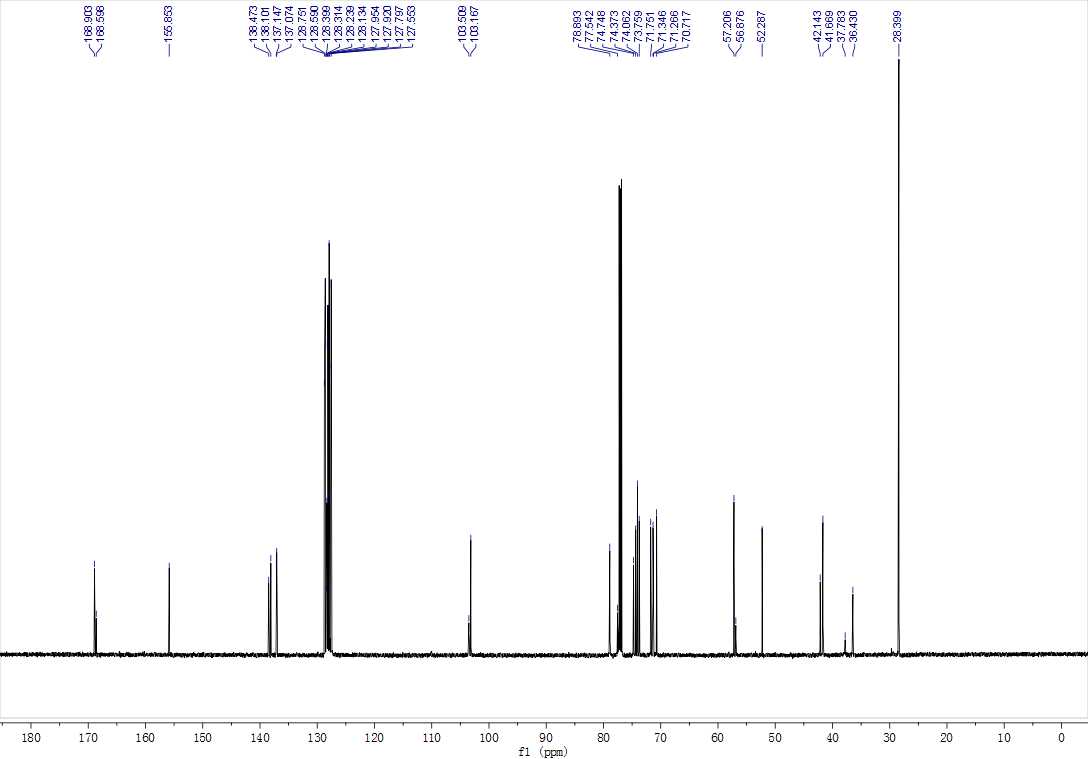
**

**
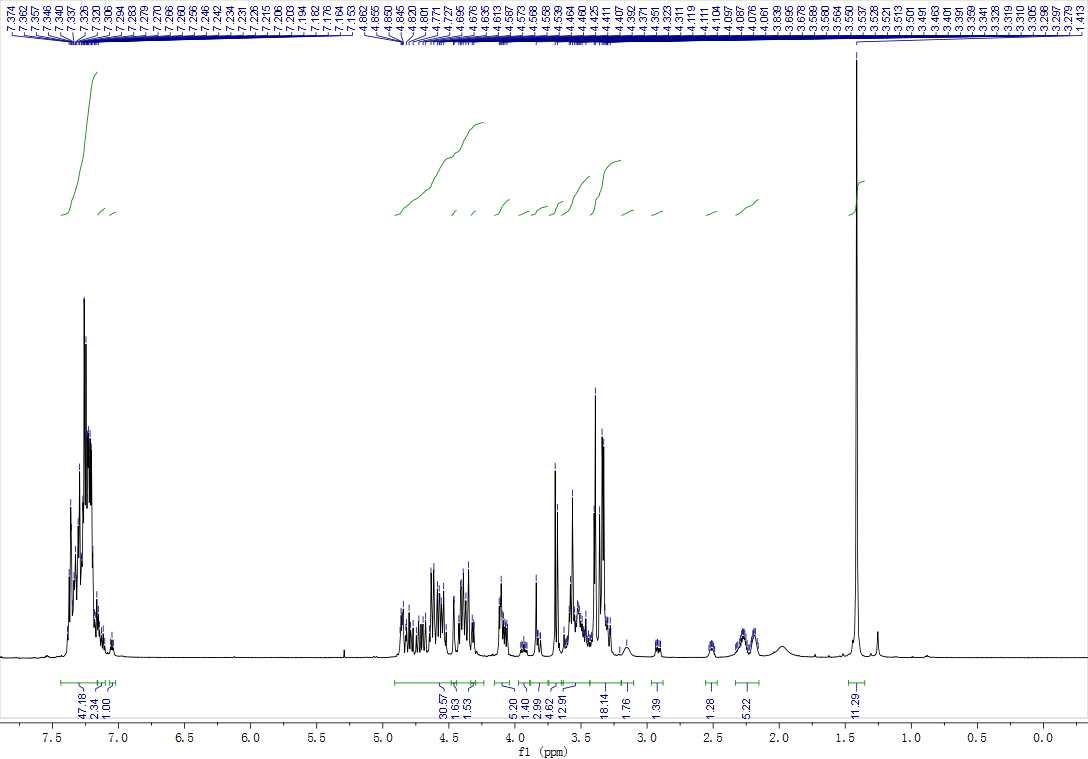
**

**
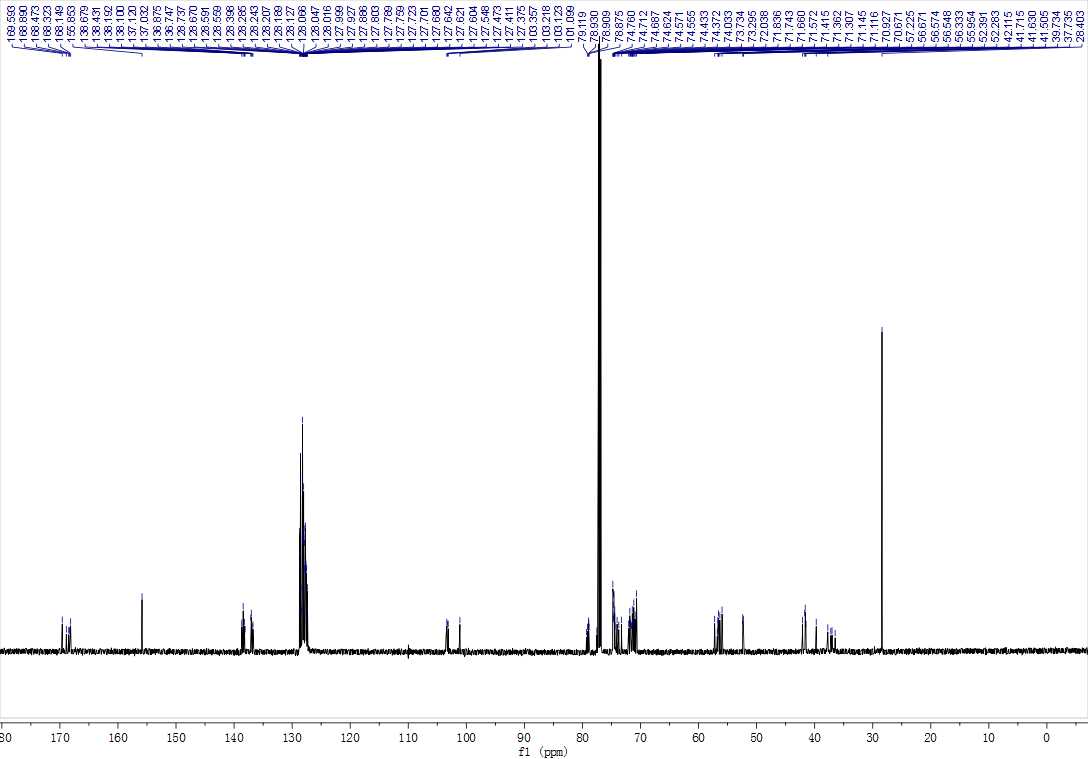
**

**
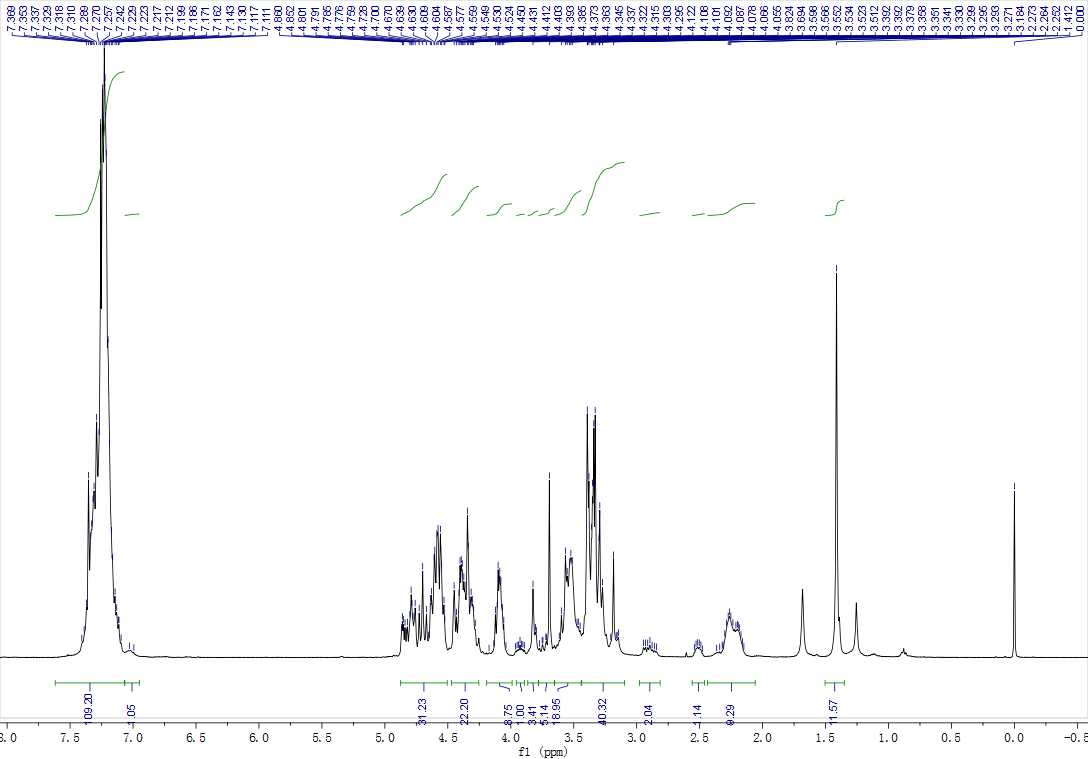
**

**
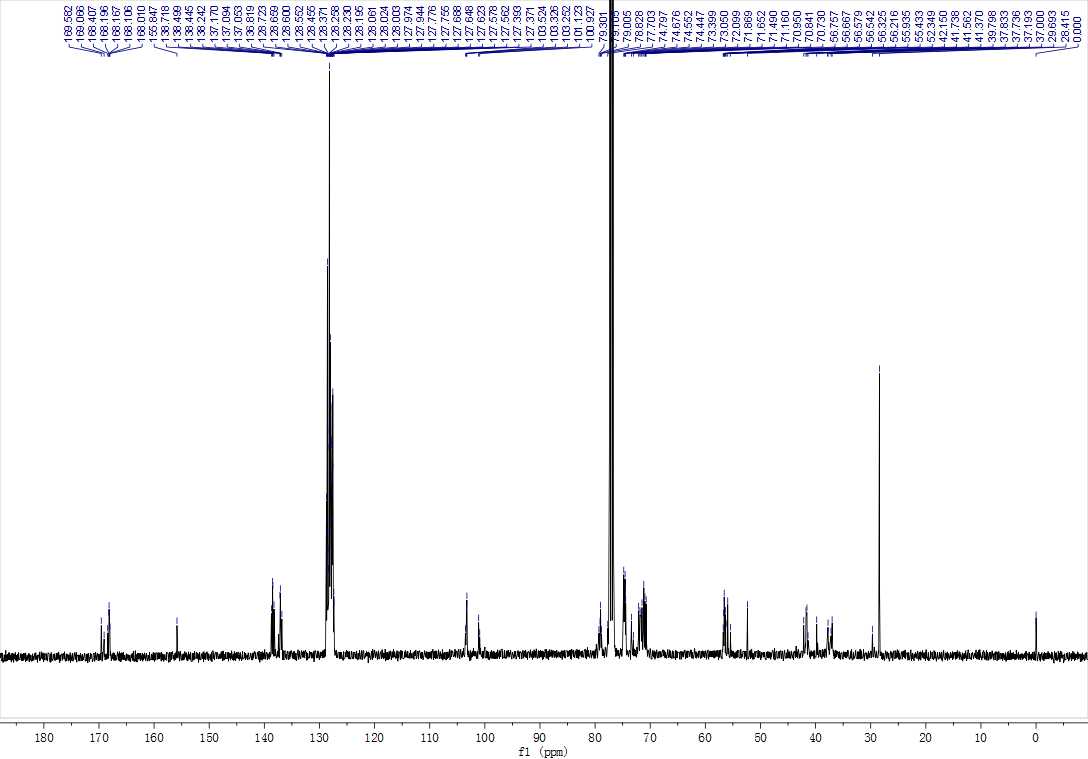
**

**
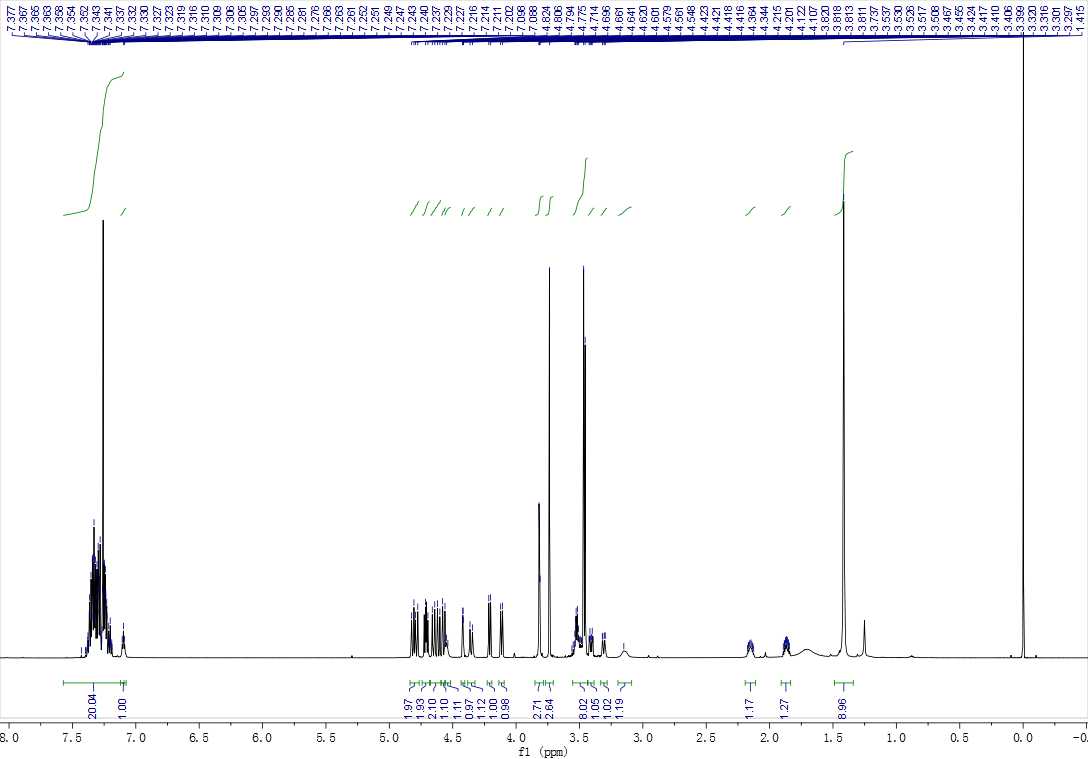
**

**
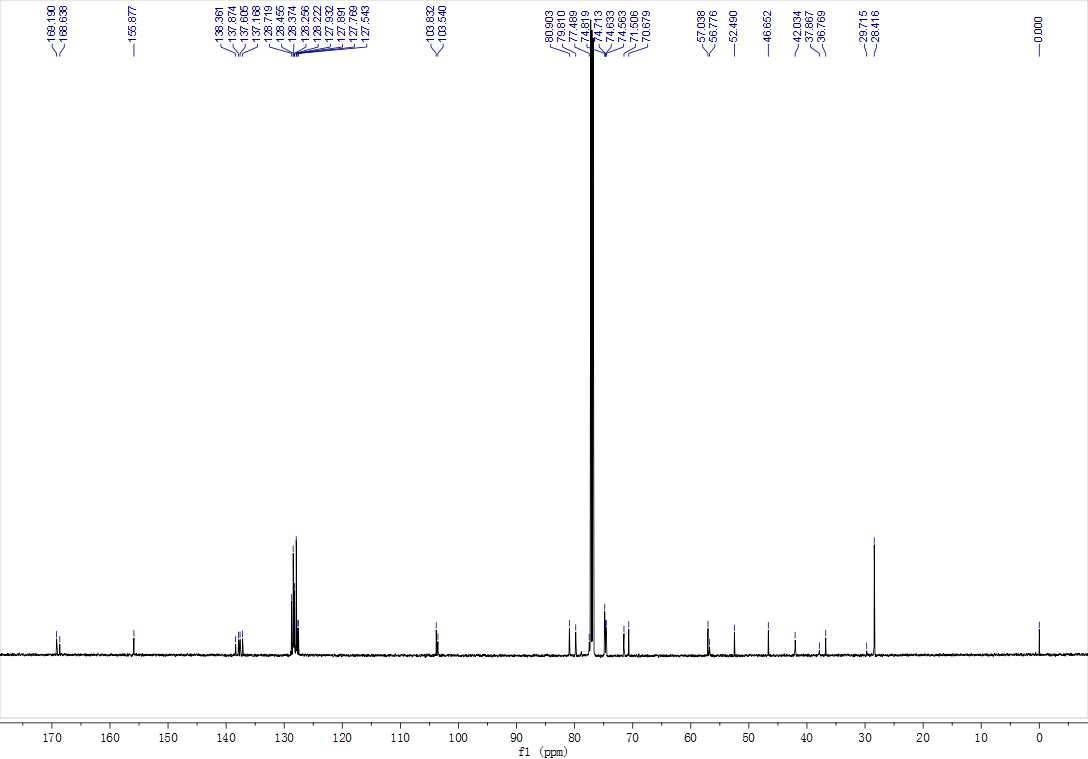
**

**
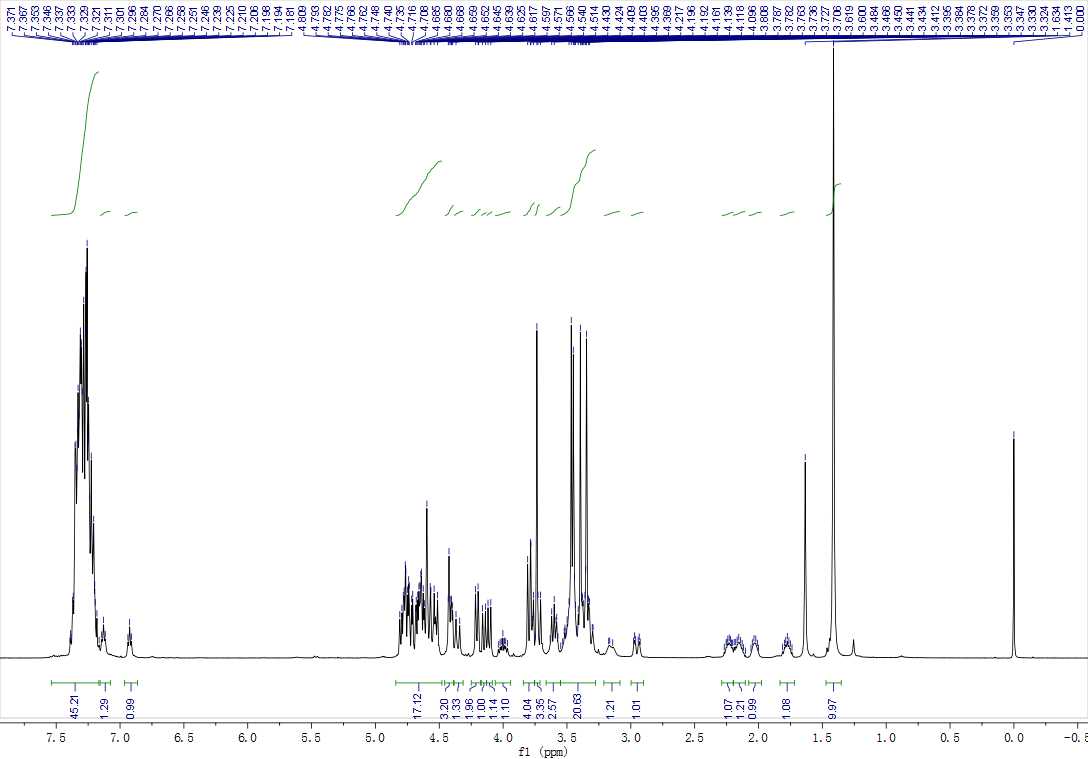
**

**
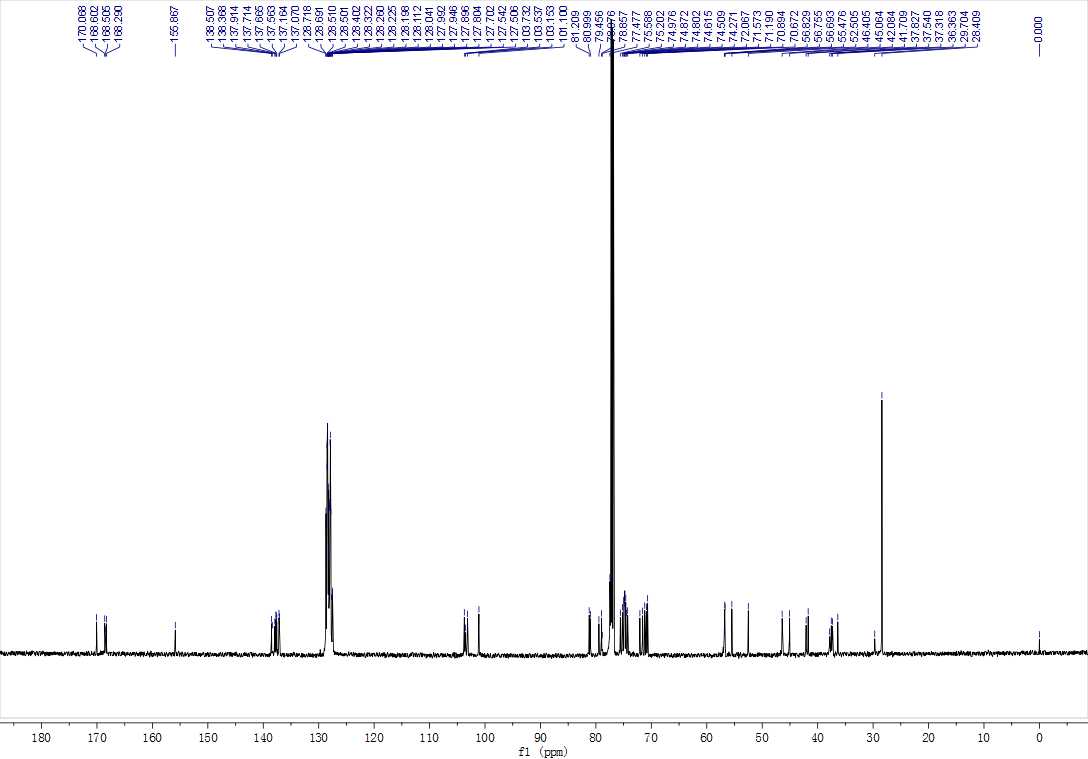
**

**
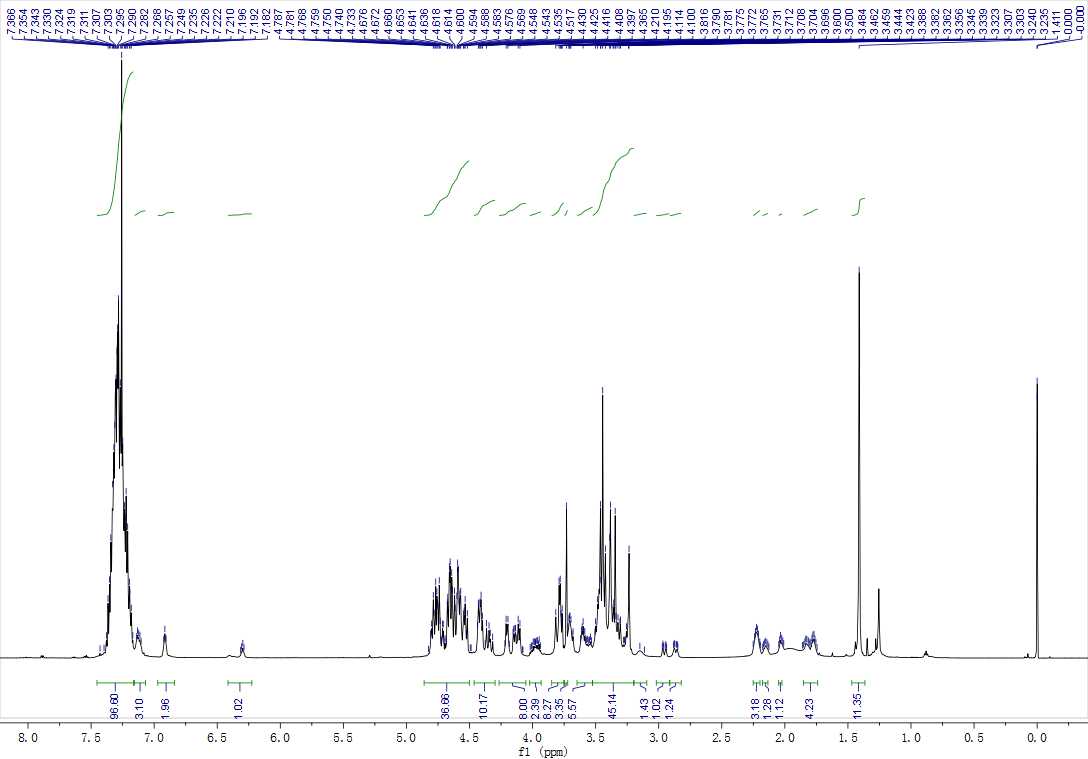
**

**
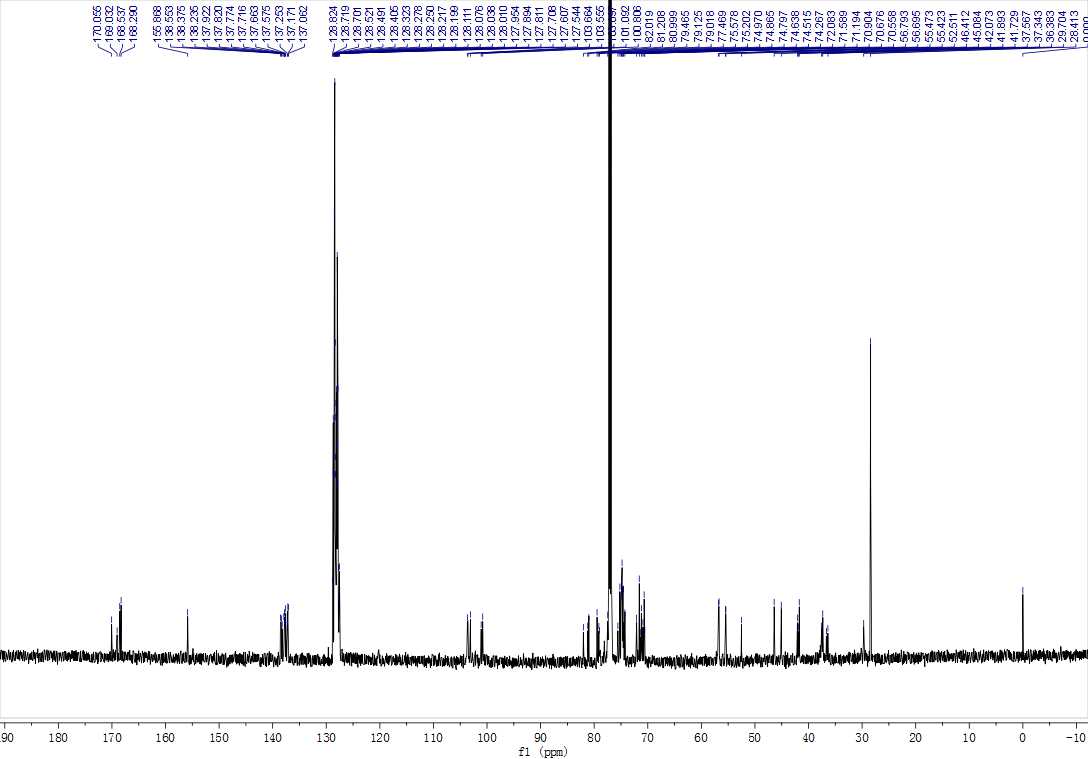
**

**4. MALDI-TOF mass spectra of new compounds**
